# Evaluating *Lachancea thermotolerans* for table olive fermentation: performance under pH and NaCl stress conditions

**DOI:** 10.3389/fmicb.2026.1846416

**Published:** 2026-05-22

**Authors:** Patricia Gil-Flores, David Penco-Parra, Joaquín Bautista-Gallego

**Affiliations:** Departamento de Ciencias Biomédicas (Área de Microbiología), Facultad de Ciencias, Universidad de Extremadura, Badajoz, Spain

**Keywords:** *Lachancea thermotolerans*, pH, predictive microbiology, sodium chloride, table olives, technological traits, yeasts

## Abstract

This study evaluates the biotechnological potential of *Lachancea thermotolerans*, a non-*Saccharomyces* yeast species commonly characterized by its ability to produce L-lactic acid, for its application in Spanish-style table olive fermentation. A total of sixty-six *L. thermotolerans* strains were assessed under varying pH and NaCl conditions, two key stress factors in table olive processing. Growth performance was determined through the analysis of growth curves and compared with those of *Wickerhamomyces anomalus* and *Candida boidinii*, two yeast species commonly isolated from table olive fermentations. To characterize strain behavior under stress conditions, growth parameters derived from the models were further analyzed using Principal Component Analysis and hierarchical clustering. These approaches enabled the classification of strains according to their tolerance to pH and salt stress, as well as the identification of groups with distinct growth kinetics. The results revealed significant variability among *L. thermotolerans* strains, highlighting a strain-dependent response to environmental stressors. A subset of strains exhibited high tolerance to both pH and NaCl conditions, demonstrating stable growth performance and suggesting a strong adaptive capacity. These findings support the potential application of selected *L. thermotolerans* strains as promising candidates for controlled table olive fermentation processes.

## 1 Introduction

Table olives are one of the most representative fermented vegetable products in Mediterranean area and have a high nutritional value, containing high amounts of fibers, amino acids, vitamins and antioxidant compounds. For this reason, these fermented vegetables can be considered as potential functional food (Lanza et al., 2021). Olives have been cultivated for thousands of years, reflecting their long-standing importance in Mediterranean diets and agro-food systems (Langgut and Garfinkel, 2022). Indeed, according to International Olive Council, the worldwide table olives production was over 3.3 million tons in 2024 [IOC (International Olive Council), 2026]. There are several processing styles: Spanish-style, Greek-style, Californian-style (Romero-Gil et al., 2013). In the Greek-style, olives are harvested at a ripe stage and then placed in brine to undergo fermentation, whereas the Californian-style involves chemical oxidation of the fruit to achieve a dark color. In contrast, in Spain, the most common methods for table olive production are directly brined, in which olives are placed directly into brine; and Spanish-style, in which the drupes are treated with a NaOH solution (2%–2.5%, w/v) to degrade oleuropein, the polyphenol responsible for the bitterness of raw fruits (Zhu et al., 2019). Then, olives are washed and placed into brine, where lactic acid bacteria, such as *Lactiplantibacillus plantarum* or *Lp. pentosus*, and yeasts, most commonly *Wickerhamomyces anomalus*, *Candida boidinii* perform the fermentation process (Romero-Gil et al., 2013). Among others, lactic acid bacteria play an important role in table olive production due to their capacity of acidification with lactic acid, which impact directly in safety, quality and flavor of the final product (Bautista-Gallego et al., 2020).

This fermentation process can occur spontaneously or be initialized with the use of starter cultures. Traditionally, these inoculums have been formed by lactic acid bacteria alone but recently the use of co-inoculation with lactic acid bacteria and yeast has been developed, contributing to prevent alterations and contribute to the organoleptic profiles of the final products (Lanza et al., 2021). Although yeast species have real potential in table olive fermentation, they can play a double role in production. On the one hand, yeast can improve and ameliorate biotechnological applications, quality and safety of the final product (Gil-Flores et al., 2025). In the other hand, yeast can produce spoilage or alteration of the final product due to the production of fermentation derived CO_2_, bad odor and flavors, and impacting in the softening and texture of the drupes if the production process is not adequately controlled (Bautista-Gallego et al., 2012).

Due to the Spanish-style processing method for table olives, microorganisms involved in fermentation, whether from starter cultures or spontaneous fermentation, must tolerate a broad range of pH conditions, from the alkaline environment generated during the NaOH debittering step to the acidic conditions that develop during fermentation. Therefore, rather than strictly acidophilic behavior, microbial adaptation to dynamic pH changes is required. Yeasts are known to maintain intracellular pH homeostasis (approximately 4.5–5.5), which is essential for cellular function (Segal-Kischinevzky et al., 2022). This adaptation involves mechanisms such as reduced membrane permeability to protons, maintenance of transmembrane proton gradients, and regulation of proton fluxes in response to external pH variations.

Halophilic or halotolerant yeasts are able to survive in environments with 2 to 20% NaCl (w/v) (Gunde-Cimerman et al., 2009). To maintain osmotic balance, these microorganisms regulate ion homeostasis through Na^+^/K^+^ transport systems and accumulate compatible solutes such as glycerol. Osmotic stress increases intracellular glycerol accumulation and membrane permeability, reducing biomass and increasing the energy expenditure required to maintain internal osmotic pressure (Segal-Kischinevzky et al., 2022).

Among the most detected yeast species in table olive fermentation, *W. anomalus* and *C. boidinii* have interesting technological characteristics. *W. anomalus* is a highly adaptable yeast frequently associated with table olive fermentations. Its metabolic versatility allows it to utilize a wide range of carbon sources and to adjust its metabolism according to environmental conditions, including oxygen availability. In addition, its tolerance to low pH and high salt concentrations favors its persistence in brined olive systems, making it a common species in spontaneous fermentations and a potential candidate for starter cultures (Basso et al., 2016; Gil-Flores et al., 2025). In addition, *C. boidinii* is another yeast commonly isolated from olive fermentations, where it contributes to the physicochemical and sensory characteristics of the final product. These species have been associated with enzymatic activities that influence aroma development and may also play a role in biofilm formation on olive surfaces. However, its presence in mixed fermentations can have both positive and negative technological implications, including potential effects on texture (Gil-Flores et al., 2025; Lanza et al., 2021; Zhu et al., 2019).

In addition, *L. thermotolerans* is a non-*Saccharomyces* yeast with notable biotechnological potential due to its common ability to acidify the medium by the production of L(+)-lactic acid, as well as to synthetize a range of metabolites that can improve the organoleptic profile of fermented products, including aroma compounds such as carbonyl compounds, organic acids, lactones, fumaric compounds and phenols (Benito et al., 2016). Due to the pH levels and sodium chloride concentrations in table olive production, *L. thermotolerans* strains intended for inoculation must be able to tolerate these stress conditions and exhibit behavior comparable to those yeasts commonly associated with industrial processes. In this context, the present study evaluates the growth performance of different *L. thermotolerans* strains under varying pH and NaCl conditions related to the initial stages of the fermentation, in comparison with *C. boidinii* and *W. anomalus*. The objectives of this work were: (i) to characterize the growth behavior of *L. thermotolerans* under different pH and salinity conditions, and to compare it with that of relevant yeast species from olive fermentations; and (ii) to identify strains with enhanced tolerance to these stress factors, with potential application as starter cultures.

## 2 Materials and methods

### 2.1 Yeast strains

A total of sixty-six *L. thermotolerans* strains belonging to the table olive microorganism collection of the University of Extremadura were used in the present study. All strains were isolated and identified between 2024 and 2025 from diverse natural sources and ecosystems (Supplementary Table S1). Single strains of *C. boidinii* and *W. anomalus*, typically associated with Spanish-style and brine-fermented green table olive fermentations, respectively, were used as controls in all subsequent experiments. Independently of the two experimental protocols used in this article, each strain was incubated at 1 mL of liquid Yeast Extract Peptone Dextrose (YEPD, 1% yeast extract, 2% peptone, and 2% glucose, w/v) at 30 °C during 2 days before the procedures, time enough to reach stationary point.

### 2.2 Inocula preparation

Inocula were prepared by inoculating a single colony of each strain into 10 mL of YEPD and incubated at 30 °C for 48 h. Then, each tube was centrifuged at 9000 g for 15 min, the pellets were washed with sterile saline solution (9 g/L), centrifuged and re-suspended again in 1 mL of a sterile phosphate buffered saline solution to obtain a concentration of about 7–7.2 log_10_ CFU/mL, which was confirmed by plating in YEPD agar plates. These microorganism suspensions were used to inoculate the different experiments by adding 3 μL of each yeast strains in 197 μL of culture media in round-bottom 96-well plates.

### 2.3 Optical density measurements and culture media

Growth curves for each yeast strain and treatment were construct using optical density (OD) measures (600 nm) taken by TECAN infinite 200Pro® (Tecan Group Ltd., Switzerland) multiplate reader at room temperature. The measures were obtained every 2 hours during 5 days. The inocula consistently remained above the detection limit of the automatized spectrophotometer, as determined by comparison with a previously established calibration curve. All experiments were tested in duplicates.

YEPD broth at different pH levels (6, 7, 8, 9, 10) were used for the study of the tolerance of each strain to the initial pH levels found in Spanish-style table olive fermentations. These culture media were adjusted with hydrochloric acid 5 N and sodium hydroxide at 5 N prior to autoclave sterilization, by using a pre calibrated Hanna edge pH meter. In the case of the salt tolerance assay, YEPD was supplemented with 0, 1, 2.5, 5, 7.5, 10, 15 and 20% (w/v) of NaCl prior to autoclave sterilization.

### 2.4 Modelling of tolerance and resistance to pH and salt

Growth curve data were normalized by subtracting the time-zero value from each data point. Additionally, data were normalized using the culture medium from the non-inoculated control curve with the same treatment. Each growth curve was built using OD previously mentioned, and non-lineal regression methods. For each strain duplicate in every experimental condition, Gompertz function was used to generate a statistical model. The Gompertz model was selected due to its suitability for describing microbial growth kinetics (Equation 1).


$$f(t)=A\;e^{-\exp(\frac{\mathrm{\mu e}}{A}(\lambda -t)+1)}$$ (1)

Where *f(t)* represents the optical density measure in each time of the growth curve; *A* represents the asymptote of the growth curve. It can be interpreted as final biomass reach by the yeast strain in a particular condition; *μ* refers the maximum growth rate, corresponding with the maximum slope of the curve during exponential phase; *λ* represent the duration of the lag phase, the time required for a microorganism for adaptation to the environment.

For this purpose, R function nlsLM() of *minpack.lm* package (Elzhov et al., 2022), which implements the Levenberg–Marquardt nonlinear least-squares algorithm, was used in R studio.

The goodness-of-fit for each Gompertz model was evaluated using the coefficient determination (R^2^) (Equation 2), calculated as:


$$R^{2}=1-\frac{\mathrm{RSS}}{\mathrm{TSS}}$$ (2)

Where RSS is the sum of squared residuals; and TSS is the total sum of squares of the observed optical density values. This pseudo-R^2^ provides a measure of how well each model explains the variation in the experimental growth curves.

For a better comparison between strain behaviors, the area under the curve (*AUC*), which can be interpreted as an estimation for total biomass accumulation over time was calculated based on the models previously described. *AUC*s were calculated using integrate() function of R package *stats* (Equation 3).


$$\mathrm{AUC}=\int_{0}^{t_{\max}}A\;e^{-\exp(\frac{\mathrm{\mu e}}{A}(\lambda -t)+1)}\;\mathrm{dt}$$ (3)

In cases where the Gompertz model was not able to provide an adequate fit to the experimental data, the *AUC* was estimated by point interpolations and integration using the function trapz() from *pracma* (Borchers, 2025) R package.

Making use of Lambert and Pearson method, non-inhibitory concentration (NIC) and minimum inhibitory concentration (MIC) values were obtained, using non-lineal regression models obtained by the processing of OD data of salt condition tested (Lambert and Pearson, 2000) (Equations 4−6). Fractional *AUC* and pH levels were fitted to an inverse Gompertz equation by nlsLM() function from *minpack.lm* package (Equation 7).


$$f(x)=A+C\cdot e^{-e^{B(x-M)}}$$ (4)

$$\mathrm{MIC}=M+\frac{1}{|B|}$$ (5)

$$\mathrm{NIC}=M-\frac{1.718}{|B|}$$ (6)

$$\mathrm{fAU}C_{i}=\frac{{\mathrm{AUC}}_{i}}{{\mathrm{AUC}}_{\text{control}}}$$ (7)

Where *f(x)* represents relative diminution of fractional area under the curve; *x* represents inhibitor concentration (salt content); *A* represents the minimum response value; *C* represents the distance between the upper and lower asymptotes; *M* represents the inflection point of the response curve; *B* represents the slope of the exponential diminution of inhibitor response; *MIC* is the Minimum Inhibitory Concentration; *NIC* is the Non-Inhibitory Concentration; *fAUC_i_* is the fractional area under the curve for condition *I*; *AUC_i_* is the area under the curve for condition *I; and AUC_control_* is the area under the curve for no inhibitor (NaCl) condition.

All parameters of the models were obtained by non-lineal regression methods minimizing the square of the differences between experimental data.

### 2.5 Clustering and PCA stratification

A new dataset was performed by joining Gompertz parameters (*A*, *μ* and *λ*) and *AUC*s of all tested yeast strains in every pH and salt conditions. Models were fitted independently for each replicate, and parameter estimates were averaged for further analysis. Data were standardized using z scale (average = 0, standard deviation = 1). PCA was performed using function PCA () from *FactoMineR* (Le et al., 2008) package. PCA was used to reduce dimensionality and identify patterns in strain behavior across environmental conditions. Hierarchical clustering of PCA results was performed using HCPC() function from *FactoMineR* package, and the number of optimal clusters was calculated automatically based on internal criteria. Visualization of PCA results including score plot and correlation circle were performed using the *factoextra* (Kassambara and Mundt, 2026) R package and hierarchical heatmaps to obtain a better description of strain stratification and global kinetics profiles. A heat map was also generated to facilitate the interpretation of clustering results using the Heatmap() function from the *ComplexHeatmap package* (Gu et al., 2016). Variable contributions to the principal components were visualized using bar plot generated with the *ggplot2* package.

### 2.6 Statistical analysis

Shapiro–Wilk and Levene’s test were performed to confirm normality assumption. Kruskal-Walis was performed to compare Gompertz parameters between different experimental conditions of pH and NaCl percent. Post-hoc Dunn test and Bonferroni test were then applicated to highlight individual differences between conditions. The *rstatix* package was used to perform statistical analyses, including shapiro_test() for normality assessment, levene_test() for homogeneity of variances, kruskal_test() for non-parametric analysis, and dunn_test() for post-hoc multiple comparisons with Bonferroni adjustment.

## 3 Results

In this work, a total of 1,494 OD curves were obtained in synthetic laboratory media to estimate the individual effects of salt and pH on the growth of 66 selected *L. thermotolerans* strains. *C. boidinii* and *W. anomalus* strains were also growth in same conditions and selected for their high survival and dominance in industrial fermentation processes. Thus, 680 and 814 models were fitted for pH and salt experiments, respectively.

### 3.1 Growth behavior of assayed strains in different NaCl and pH levels

**Figure 1:**
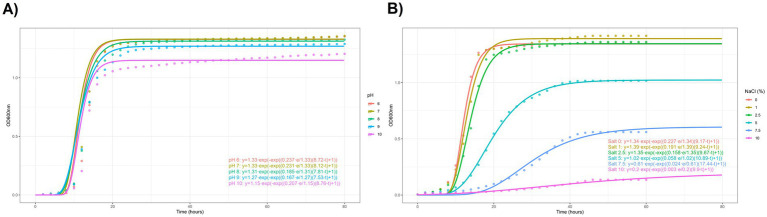
**(A)** Descriptive analysis of experimental conditions with pH. Representative model of Gompertz non-linear regression method of BMA122. Each equation present in the figure corresponds to an individual pH level of the same strain. **(B)** Descriptive analysis of salt experimental conditions. Representative model of Gompertz non-linear regression method of BMA 122. Each equation present in the figure corresponds to an individual salt level of the same strain.

The curves were fitted to a Gompertz model for a better comparison of the growth behavior at different pH and salt conditions (Figures 1A,B). *C. boidinii* and *W. anomalus* was also fitted using the same method for further comparison. All *L. thermotolerans* strains shawn similar behavior and fitted correctly to Gompertz non-lineal regression models. Only 15 and 20% (w/v) of all strains, along with *C. boidinii* at 10% w/v of salt content conditions could not be fitted due to the lack of growth.

#### 3.1.1 Growth behavior of assayed strains in different pH levels

For the analysis of the effect of pH in growth behavior of *L. thermotolerans* strains, five different media, at pH 6, 7, 8, 9, and 10 were inoculate with each studied strain. The Gompertz model provided a robust fit for all strains growth curves in every pH level, with *R^2^* values ranging from 0.949 to 0.999 (Supplementary Table S2). These results confirm the high accuracy of the non-linear regression in describing the microbial growth kinetics across all tested pH levels.

**Figure 2:**
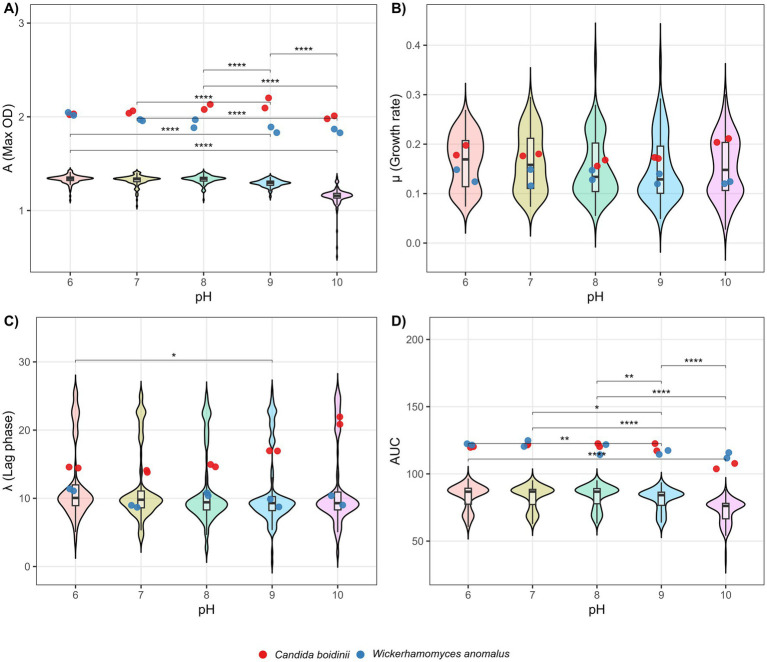
Violin plots of Gompertz parameters at different pH levels. **(A)** *A* parameter comparison. **(B)** *μ* parameter comparison. **(C)** *λ* parameter comparison. **(D)** *AUC* comparison. Statistical significance between experimental groups was obtained using Dunn’s post-hoc test and Bonferroni correction. Significance levels are indicated as follows: *p* ≤ 0.05 (*), *p* ≤ 0.01 (**), *p* ≤ 0.001 (***), and *p* ≤ 0.0001 (****).

Statistical tests demonstrate significant differences between Gompertz parameters of *L. thermotolerans* strain’s growth curves depending on pH condition, being *A* (asymptote) the most affected parameter by pH (Figure 2A). In this context, differences between parameters *A* at pH 6, 7, and 8 did not exhibit significant difference, being relatively stable at this range. In the other hand, pHs upper than 8 reveal a progressive and significant diminution of *A* (Figure 2A). Furthermore, the *AUC* exhibited the same behavior as *A*, being a constant value at pH 6, 7, and 8. In contrast, *AUC* at pH 10 have significant differences with all other values, suggesting that a diminution in *AUC* start at a pH level between 8 and 10 (Figure 2D). In the other hand, *μ* and *λ* exhibit a more constant behavior, only appearing slight significant difference between pH 6 and 9 lag phase duration (Figures 2B,C). These statistical results are shown in Supplementary Table S3.

#### 3.1.2 Growth behavior of assayed strains in different salt concentrations

**Figure 3:**
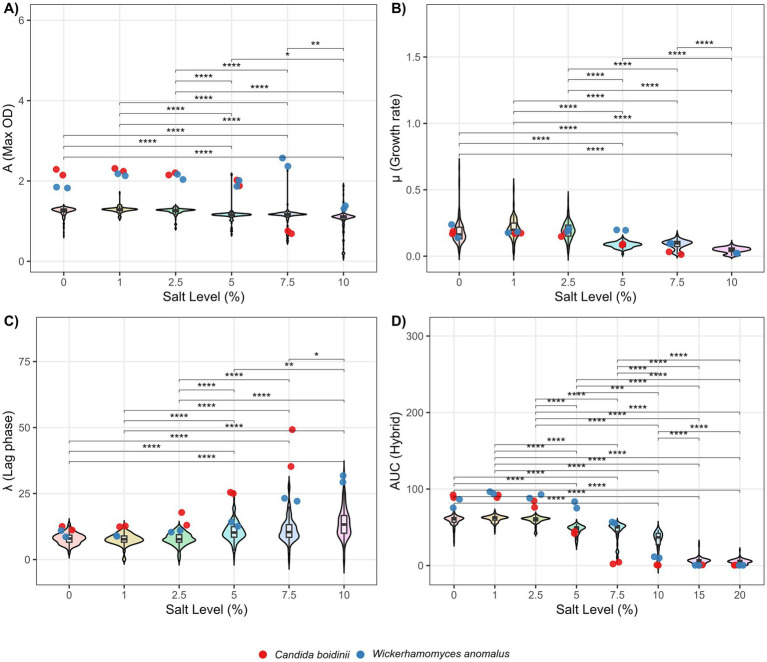
Violin plots of Gompertz parameters at different salt percent levels. **(A)** *A* parameter comparison. **(B)** *μ* parameter comparison. **(C)** *λ* parameter comparison. **(D)** *AUC* comparison. Statistical significance between experimental groups was obtained using Dunn’s post-hoc test and Bonferroni correction. Significance levels are indicated as follows: *p* ≤ 0.05 (*), *p* ≤ 0.01 (**), *p* ≤ 0.001 (***), and *p* ≤ 0.0001 (****).

Salt conditions were tested by inoculating the same amount of yeast in culture Medias elaborated with 0%, 1%, 2.5%, 5%, 7.5%, 10%, 15%, and 20% (w/v) of NaCl. Gompertz models refer to salt level also exhibit good R^2^ values, ranging from 0.9060 to 0.9998 (Supplementary Table S4). All experimental conditions were able to be fitted by Gompertz function except 15 and 20% of NaCl (w/v) due to an insufficient growth of the majority yeast strains. For the same reason, *Candida boidinii* at 10% (w/v) of salt could not be fitted. Furthemore, statistical tests demonstrate a significant effect of NaCl in Gompertz parameters distributions. The obtained results suggest a higher inhibitory effect of salt content than pH (Figure 3). Asymptote stay apparently constant between 0 and 2.5% of salt percentage. In contrast, beginning on 5% of salt levels, the result exhibits a significant gradual diminution of *A*, revealing that moderate salt concentration limit maximum growth capacity of *L. thermotolerans* strains populations (Figure 3A). What concern to *μ*, there are significant differences between most condition groups, however it stay constant between 0 and 2.5% (w/v) of NaCl. At 5% level, *μ* decrease drastically, highlighting an evident inhibitory effect in cell division at moderate NaCl concentrations (Figure 3B). Relative to *λ*, no significant differences were found between 0 and 2.5% of NaCl. However, a greater effect was observed at higher concentrations, particularly pronounced between 7.5 and 10% (w/v). This behavior suggests an initial difficulty in adapting to these NaCl levels (Figure 3C). Concerning the *AUC*, it exhibits a constant and significant decrease, highlighting the decrease of biological fitness with salt percent elevation. Particularly, at 15 and 20% (w/v) of NaCl levels, most of *L. thermotolerans* and control strains tested in this work could not growth, along with *C. boidinii* at 10% (w/v) of NaCl content, reason why Gompertz regressions could failed and areas under the curve at this point was estimated via point interpolations and integration (Figure 3D). These statistical results are shown in Supplementary Table S5.

### 3.2 Tolerance to salt and pH

**Figure 4:**
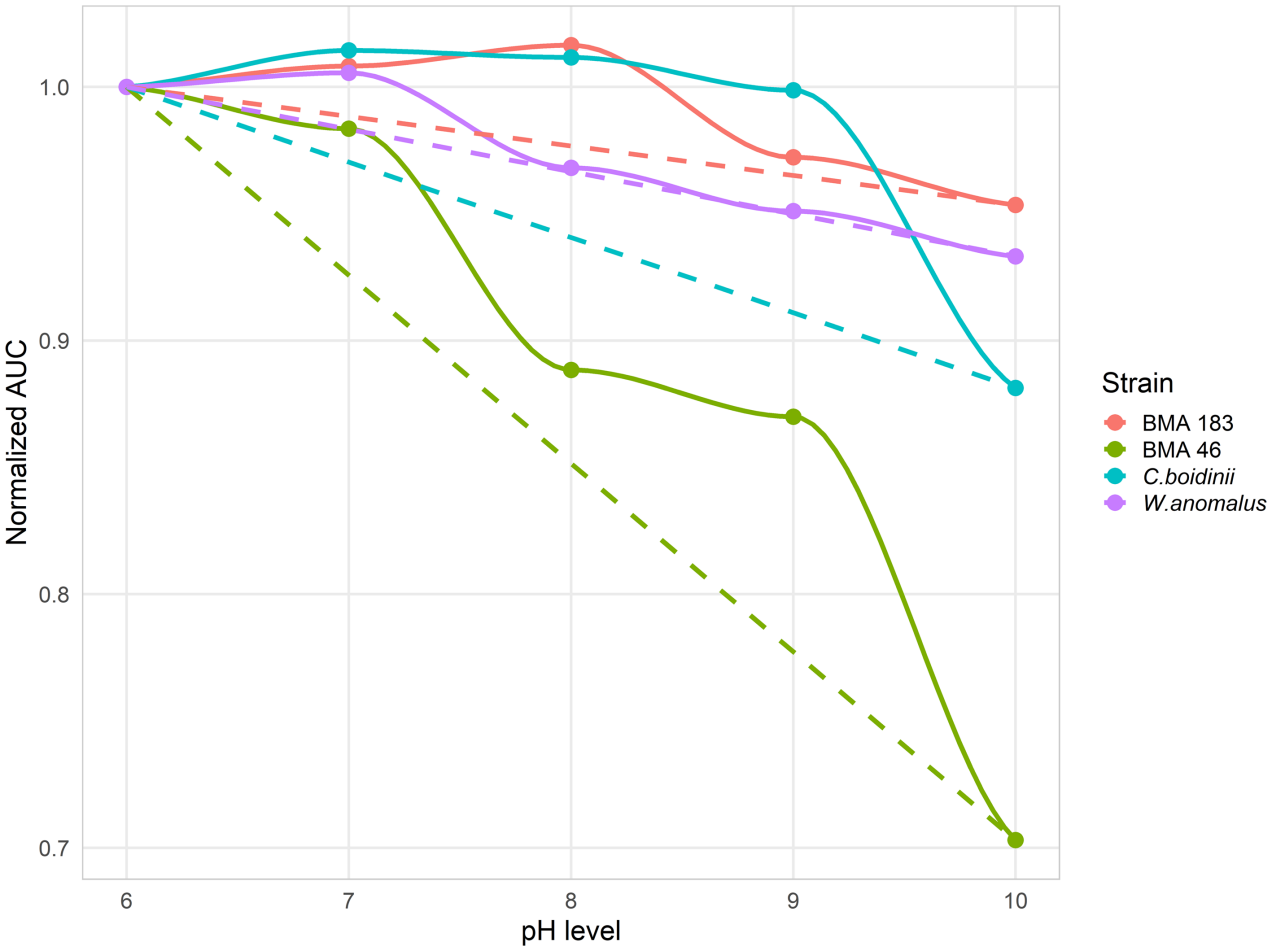
Normalized areas under the curve vs. pH level of BMA 183 and BMA 46, the *L. thermotolerans* strains with the best and worst performance under the tested pH conditions respectively, and *Wickerhamomyces anomalus* and *Candida boidinii*, the controls used for comparative analysis. The discontinuous line represents the diminution percentage of *AUC*s between pH 6 and pH 10.

Overall, pH response showed a stable behavior across the tested conditions. Since pH did not drastically reduce yeast survival, inverse non-linear Gompertz regression models could not be fitted, and NIC and MIC values could not be calculated. In this case, tolerance was compared using the percentage of decrease in the normalized *AUC* between pH 6 and pH 10, corresponding to the lowest and highest experimental conditions tested. Thus, the pHs results revealed that BMA 183 exhibited very similar behavior to both *C. boidinii* and *W. anomalus*, the yeasts used as controls, with only a 4.7% decrease in biological fitness between conditions. On the other hand, the least promising strain was BMA 46, showing a 29.7% reduction in *AUC* between conditions (Figure 4). The rest of the strains displayed intermediate tolerance levels. Overall, the results indicate differences in optimal pH values, ranging from pH 6 to pH 8, under the studied conditions, suggesting that this parameter is strain-dependent.

**Figure 5:**
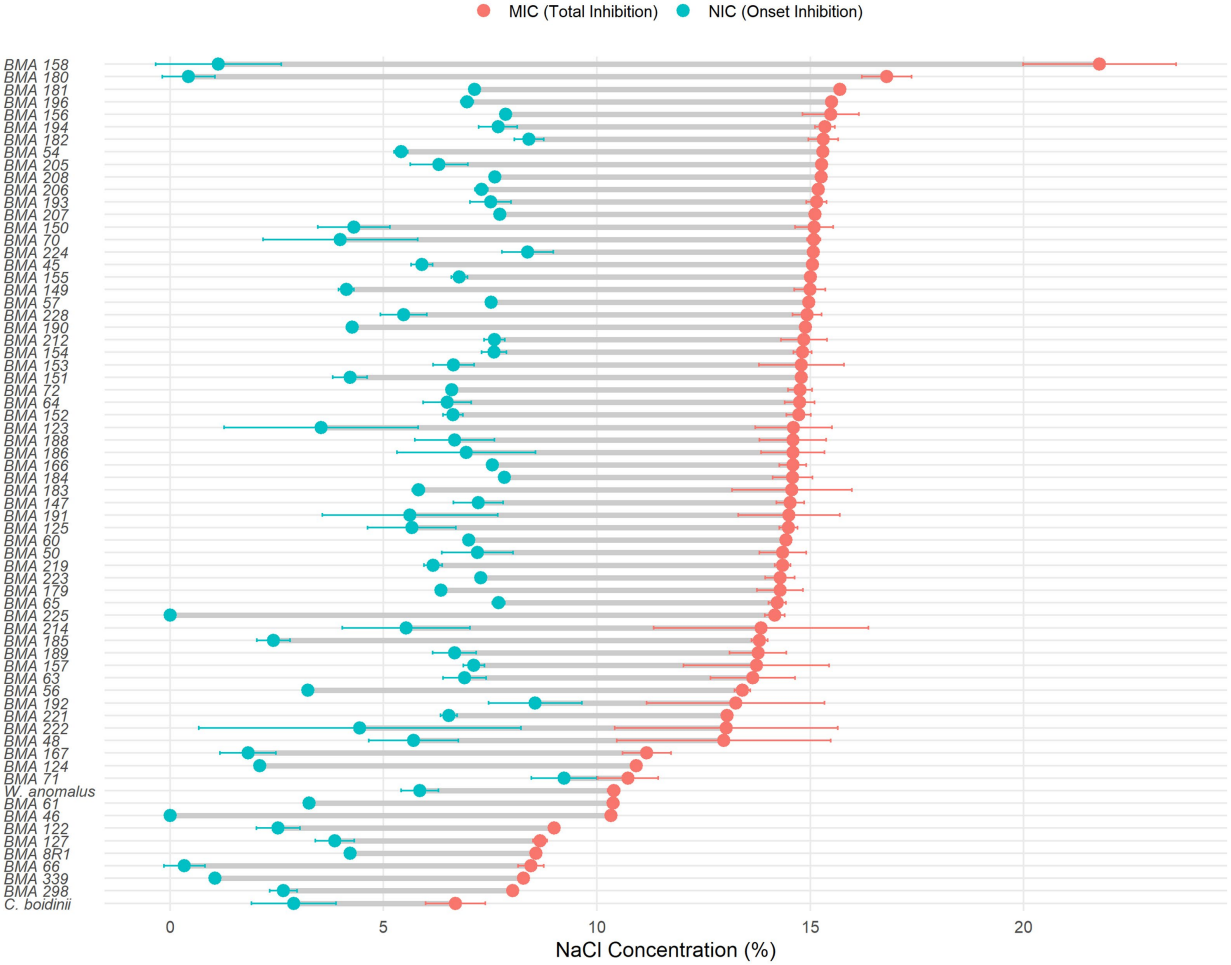
Salt tolerance thresholds of yeast strains based on NIC and MIC values. The horizontal dot plot represents the NIC (blue circles) and the MIC (red circles) values for each strain across a NaCl concentration gradient (0–25% w/v). Strains are ordered by their maximum salt tolerance (MIC). Error bars indicate the 95% confidence intervals for each threshold. Reference strains *Wickerhamomyces anomalus* and *Candida boidinii* are included for comparative purposes.

On the contrary, inverse Gompertz regressions were performed for this purpose with the average *AUC*s of salt growth curves of yeast duplicated. Regarding salt tolerance, NIC and MIC values were calculated based on the fitted models. Negative theoretical NIC values were set to 0 to allow for biological interpretation. The results revealed variability in strain behavior, suggesting strain-dependent salt tolerance. In many cases, MIC values exceeded a salt content of 10% (w/v), highlighting the resilience of *L. thermotolerans* strains (Figure 5). In contrast to *L. thermotolerans*, *C. boidinii* and *W. anomalus* exhibited lower MIC and NIC values, indicating a higher impact of salt concentration on their growth behavior. Inverse Gompertz regression models parametres are summerisez in Supplementary Table S6.

### 3.3 Principal components analysis and hierarchical clustering of Gompertz parameters and areas under the curves

**Figure 6:**
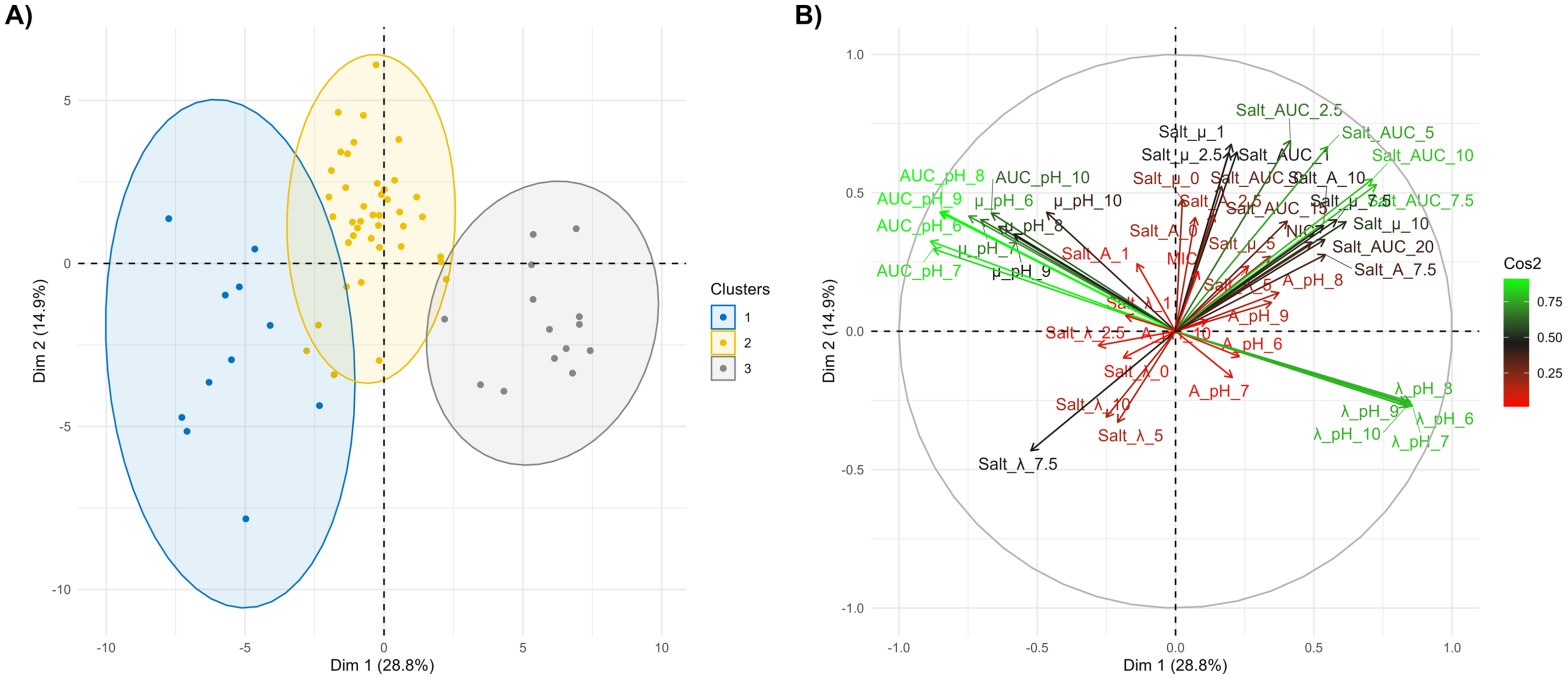
PCA plots of yeast strains based on Gompertz growth and *AUC*s. **(A)** PCA score plot. Strains are projected onto the first two principal components. Colors refer to phenotypic groups identified by hierarchical clustering on principal components (HCPC). Ellipses represent the 95% confidence intervals for each cluster. **(B)** PCA correlation circle of Gompertz growth parameters and *AUC*. Vectors represent loadings of the 53 variables on the first two principal components. The length of the vector indicates the strength of the correlation between the parameters (*A*, *μ,* *λ*, *AUC, MIC* and *λ* *NIC*) and dimensions. Vectors in the same direction indicate positive correlation, and their proximity to the unit circle represents the quality and weight of the variable.

Once the parameters derived from the Gompertz models described in the previous sections were obtained, they were subjected to Principal Component Analysis (PCA) and Hierarchical Clustering (Figure 6A). This approach was applied to assess the presence of groups with key characteristics related to growth kinetics. The first two principal components explained 28.8 and 14.9% of the total variability, respectively (Figure 6A). The aim of this analysis was to classify *L. thermotolerans* strains based on their behavior under stress conditions and to identify the most resistant group to pH and salt concentration.

The first principal component (28.8%) was strongly influenced by *AUC* and *λ* parameters from pH models. Specifically, *AUC_pH_6* (5.68%) and *AUC_pH_7* (5.55%) are the most influential variables, followed closely by adaptation times such as *λ_pH_7* (5.31%) and *λ_pH_6* (5.27%). These results suggest that the ability to reach high population densities and the speed of adaptation to neutral and slightly acidic pH conditions represent the main source of variation among groups (Figure 6B, Supplementary Figure S1).

In contrast, the second principal component (14.9%) was dominated by parameters related to salt stress and specific growth rates. In this dimension, *Salt_AUC_5* (6.22%) and *Salt_AUC_2.5* (6.64%) showed the highest contributions, together with *Salt_μ_1* (6.38%) and *Salt_μ_2.5* (5.84%). Variables related to extreme conditions or optimum growth, such as *Salt_μ_0* (0.004%) and MIC (0.048%), contributed negligibly to the overall variance. This fact indicated that yeast strain behavior remains relatively uniform under low-stress conditions and diverges only under moderate stress levels. Under these conditions, distinct clusters emerge, primarily driven by differences in growth efficiency under moderate salt concentrations and pH variation (Figure 6B).

**Figure 7:**
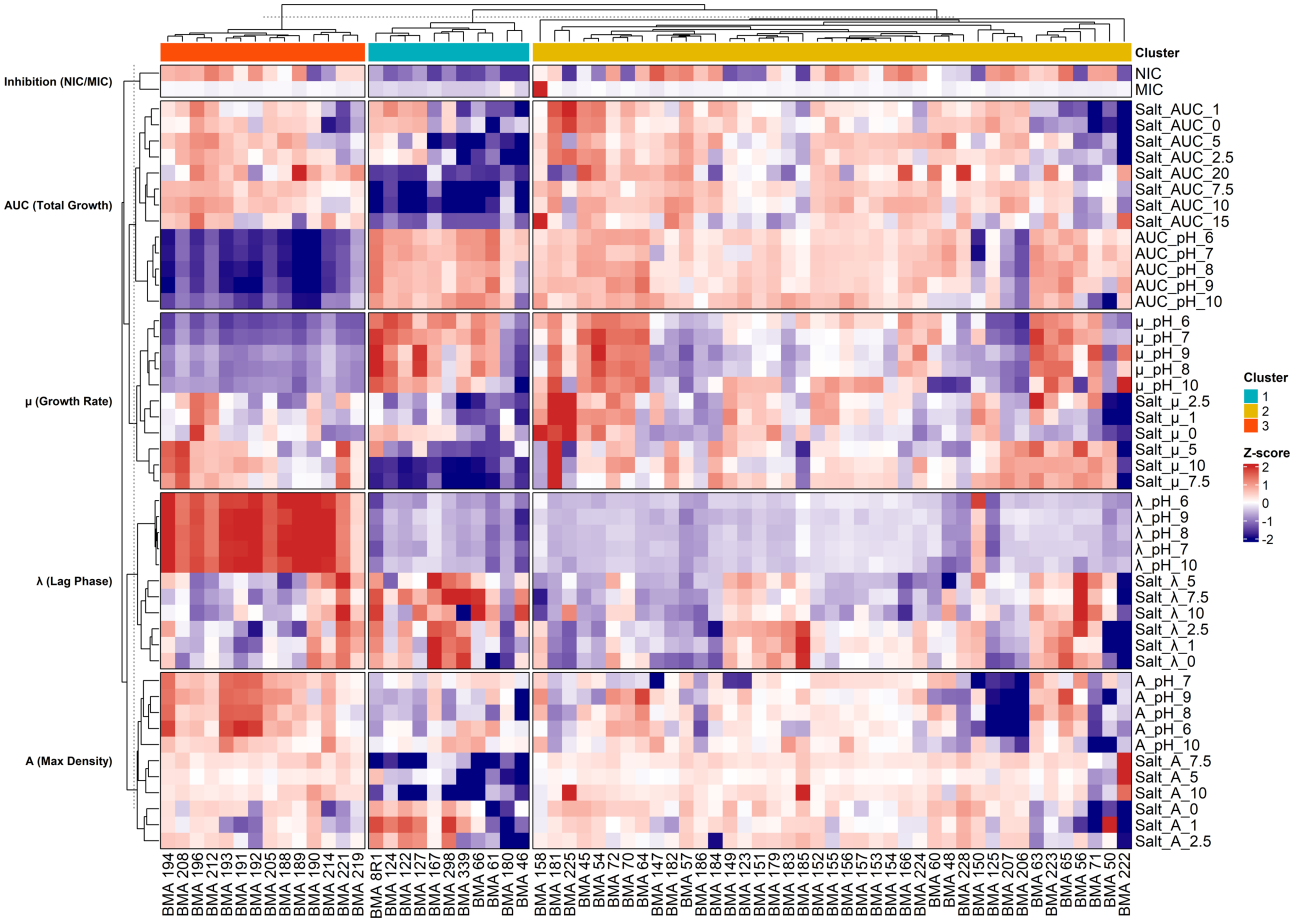
Heatmap of phenotypic profiles of *Lachancea thermotolerans* strains based on growth parameters. Data represent *Z*-score normalized values of Gompertz parameters (*A, μ, λ*), *AUC*, and inhibition indices (NIC/MIC). Rows are categorized by growth parameter type, while columns represent individual yeast strains grouped by phenotypic clusters (1–3) identified via HCPC. Dendrograms represent hierarchical clustering based on Euclidean distance and Ward’s method.

Hierarchical clustering has applied to the PCA results, enabling the identification of three different clusters (Figure 7). Cluster 1 comprised eleven *L. thermotolerans* strains characterized by low tolerance and reduced growth capacity, primarily reflected in decreased growth rates. In contrast, Cluster 3 included fourteen yeast strains that were highly affected by pH levels, as evidenced by their lower *AUC* values compared to the other clusters. In this group, the most affected Gompertz parameter appeared to be *λ*, which remained consistently high across most pH conditions. The marked extension of the lag phase in Cluster 3 under specific pH conditions suggests a higher energetic cost associated with cellular adaptation and pH homeostasis. This may represent a limiting factor in competitive fermentations where rapid onset of growth is essential to outcompete spoilage microorganisms. Lastly, Cluster 2, comprising forty-one yeasts strains, exhibited more balanced growth parameters across all salt and pH conditions, showing a high degree of phenotypic plasticity and metabolic robustness. The stability of growth dynamics under fluctuating pH and salinity conditions suggests an enhanced homeostatic capacity, a key trait for potential industrial starter cultures in unpredictable and variable fermentation environments.

## 4 Discussion

This study evaluates the potential application in table olive fermentation of a collection of *L. thermotolerans* strains, a yeast species of increasing biotechnological interest due to its well-documented capacity to produce L(+)-lactic acid (Benito et al., 2016; Snyder et al., 2021). L-lactic acid is a key organic acid naturally present in table olive as a result of lactic acid bacteria metabolism during fermentation (Fernandez et al., 1997; Hurtado et al., 2012). In Spanish-style table olive processing, the combined production of lactic acid by lactic acid bacteria and *L. thermotolerans* inocula could contribute to a faster decrease of the initially high pH generated during NaOH debittering. This hypothesis suggests the potential use of selected *L. thermotolerans* strains as a biological alternative to the addition of chemical acidifiers commonly employed in table olive industry, thereby reducing production costs and improving process sustainability (Gil-Flores et al., 2025). To assess this potential, it is essential to characterize the ability of this yeast species to grow under increasing NaCl concentrations and alkaline pH conditions, which are critical parameters in Spanish-style olive processing.

The results highlight the notable resistance of *L. thermotolerans* to both pH and salt concentrations, with a more pronounced resistance to pH than to NaCl. Growth remained relatively stable within the pH range between 6 and 8, as supported by the minimal variation in the parameters of the fitted Gompertz models. However, a significant decline was observed from pH 9 onwards, with the asymptote (*A*), representing the maximum biomass yield, being the most affected parameter. Consequently, the *AUC* was also reduced, given its dependence on *A* (Figure 3A). In contrast, *μ* and λ remained relatively constant across the tested pH range. These results suggest that *L. thermotolerans* is capable of surviving and maintaining metabolic activity during the early stages of Spanish-style table olive processing. Traditionally, starter cultures are inoculated after pH adjustment to approximately 7, following NaOH treatment, during which the medium may reach pH values close to 10 (Fernandez et al., 1997). This pH decrease can require several days (5–7 days), or alternatively, can be accelerated through the addition of chemical acidifiers. In this context the use of *L. thermotolerans* could represent a viable strategy to replace or reduce chemical acidification, as its tolerance to high pH conditions would enable earlier inoculation immediately after NaOH treatment. This approach could shorten fermentation times, enhance process efficiency, and contribute to more sustainable table olive production systems (Corsetti et al., 2012; Giavalisco et al., 2024; Heperkan, 2013).

Typically, high-salinity brines (10–12% w/v NaCl) are applied during the early stages of processing following successive washings aimed at removing residual NaOH. Over time, salt equilibrates between the brine and the olive tissue - whose epidermal integrity has been compromised by alkaline treatment-resulting in final concentrations of approximately 6%–7% (w/v) within 4–7 days (Fernandez et al., 1997). Regarding NaCl tolerance, *L. thermotolerans* is slightly more sensitive to salinity than to pH variations under the tested conditions. Maximum biomass production remained relatively stable at low NaCl concentrations (0%–2.5%, w/v) but exhibited a progressive and significant decline from 5% (w/v) NaCl onwards (Figure 3A). Similarly, *μ* showed significant differences between most conditions, although it remains constant within the 0%–2.5% (w/v) range. At 5% (w/v) NaCl, *μ* decreased markedly, indicating a clear inhibitory effect of moderate salinity on cellular division (Figure 3B). In contrast, *λ* did not display significant differences at low NaCl levels (0%–2.5%, w/v), but increased notably at higher levels, particularly between 7.5% and 10% (w/v), suggesting increased physiological stress and the need for cellular adaptation under these conditions (Figure 3C) (Romero-Gil et al., 2013). These variations in growth parameters directly impacted *AUC*, reflecting an overall reduction in growth performance as NaCl level increased. At high chloride salt concentrations (15 and 20%, w/v), salinity became the dominant limiting factor, completely inhibiting the growth of *L. thermotolerans*. Under these conditions, Gompertz models could not be reliably fitted due to the absence of measurable growth. Although NaCl concentration exerts a stronger inhibitory effect than pH, *L. thermotolerans* strains demonstrated the ability to tolerate moderate salt amounts (5%–10%, w/v). This tolerance range is particularly relevant for table olive fermentation, where salt levels once equilibrated typically range between 6% and 7% (w/v) (López-López et al., 2023), supporting the potential application of some strains belonging to this specie under industrial processing conditions

Following the characterization of the growth response of *L. thermotolerans* under individual salt and pH stress conditions, its tolerance was comparatively evaluated against *W. anomalus* and *C. boidinii*. This comparative approach provides a relevant ecological and technological framework to assess the competitiveness and potential functionality of *L. thermotolerans* within established fermentation consortia. Regarding the pH comparison, the *fAUC*, normalized to pH 6, were contrasted with *L. thermotolerans*, *W. anomalus*, and *C. boidinii*. These results revealed a high degree of intra-specie variability within *L. thermotolerans*. This result agrees with those obtained by Hranilovic et al. (2018) who demonstrated a high degree of intra-species phenotypic variability, underscoring the importance of strain selection for industrial application. Specifically, the most pH-tolerant strain (BMA 183) exhibited only a 4.7% reduction in fitness between pH 6 and pH 10, whereas the least tolerant strain (BMA 46) showed a substantially higher decrease (29.7%), indicating marked phenotypic heterogeneity. This observation is consistent with previous reports in non-*Saccharomyces* yeasts, where substantial intra-species variability in stress tolerance, metabolism, and ecological performance has been widely reported (Fleet, 2008). Furthermore, this analysis demonstrated that the optimal pH for *L. thermotolerans* growth is strain dependent, as evidenced by the distinct maxima in normalized *AUC* values. BMA 183 reached its optimum at pH 8, while BMA 46 exhibited maximal performance at pH 6 (Figure 4). Such variability has also been described in wine-related studies, where *L. thermotolerans* strains display different metabolic and physiological responses depending on environmental conditions, including pH and nutrient availability (Vicente et al., 2025). When compared to *W. anomalus*, which showed 6.7% reduction in fitness between pH 6 and 10, *L. thermotolerans* displayed a similar behavior or even better tolerance to alkaline conditions. This is particularly relevant given that *W. anomalus* is widely recognized for its robustness and adaptability in fermented food ecosystems, including table olives (Garijo et al., 2009; Walker, 2011). In contrast, *C. boidinii* exhibited even lower performance, with its *fAUC* decreasing by 11.9% between the same pH levels, suggesting a lower tolerance to alkaline stress. Notably, this decrease in *C. boidinii* performance was particularly pronounced between pH 9 and 10, indicating a narrower physiological tolerance range compared to both *L. thermotolerans* and *W. anomalus*. These findings are consistent with previous ecological studies, indicating that *C. boidinii*, although commonly present during olive fermentation, tends to dominate under more moderate conditions and may be less competitive under extreme environmental stress (Wang et al., 2012). Overall, the comparative analysis suggests that *L. thermotolerans* exhibit a level of pH tolerance comparable to, or in some cases exceeding, key yeast species traditionally associated with table olive fermentation. This resilience, combined with its potential capacity for lactic acid production, reinforces its suitability as a multifunctional starter culture capable of contributing both to acidification and microbial stability during the early stages of processing (Gil-Flores et al., 2025; Sánchez-Suárez et al., 2025). Importantly, the observed strain-dependent variability underscores the need for targeted selection and screening programs to identify the most suitable candidates for industrial application.

With respect to NaCl tolerance, MIC and NIC values were estimated from the previously fitted non-linear regression models providing quantitative descriptors of yeast performance under NaCl stress (Lambert and Pearson, 2000; Romero-Gil et al., 2013). In this context, for most *L. thermotolerans* strains, MIC values ranged between 10 and 15% (w/v) of NaCl, confirming a generally high tolerance to saline environments. However, NIC values exhibited considerable variability among strains, further supporting the existence of significant intra-species heterogeneity. Notably, several high-performing strains, such as BMA158 and BMA180, displayed exceptionally elevated theoretical MIC values, which clearly exceed the salt concentrations typically reached in equilibrated table olive brines (≈6%–7% w/v) (Fernandez et al., 1997; Bautista-Gallego et al., 2013). These results indicate that selected *L. thermotolerans* strains possess a substantial safety margin for growth under industrial conditions, reinforcing their suitability for application in high-salinity fermentations. *W. anomalus* exhibited a high MIC (10.4%) value, showing a high salt tolerance although still lower than those observed for the best-performing *L. thermotolerans* strains. This suggests that, while *W. anomalus* is considered a robust and adaptable species in olive fermentations, certain *L. thermotolerans* strains may surpass it in terms of extreme halotolerance. In contrast, *C. boidinii* showed a significantly lower MIC value (6.69%, w/v), mirroring the behavior of the most salt-sensitive *L. thermotolerans* strains. This reduced tolerance is consistent with earlier findings by Romero-Gil et al. (2013), who reported NIC and MIC values of 57.7 g/L and a MIC of 98.0 g/L, respectively, for *C. boidinii*. Such values confirm its comparatively limited fitness in high-salinity environments and help explain its ecological prevalence in later fermentation stages, when salt concentrations have already equilibrated and environmental stress is reduced (Arroyo-López et al., 2012a,b; Romero-Gil et al., 2013). Although only a single strain of *W. anomalus* and *C. boidinii* was evaluated, and intraspecific variability may lead to strain-dependent differences, both strains are representative of those typically encountered in industrial fermentation processes. Importantly, the wide range of MIC and NIC values observed among *L. thermotolerans* strains underscores the critical importance of strain-level selection, as only a subset of isolates exhibits the level of robustness required to withstand the most extreme processing conditions. Future studies should therefore focus on linking these phenotypic traits with genomic and metabolic markers to facilitate the targeted development of optimized starter cultures.

Following the characterization of *L. thermotolerans* growth under individual stress conditions, the next step of this study was to stratify the strains and identify those exhibiting robust tolerance to both pH and salinity stressors by using multivariate statistical approaches, including PCA and Hierarchical Clustering (Jolliffe and Cadima, 2016). This approach allowed the identification of the main physiological drivers underlying intra-species variability in *L. thermotolerans*. Notably, pH tolerance (captured by PC1) and salinity response (captured by PC2) behaved as largely independent axes of variation, suggesting that the cellular mechanisms governing adaptation to alkaline stress and osmotic stress are at least partially decoupled. This observation is consistent with previous studies in yeasts, where pH homeostasis and osmoadaptation are regulated by distinct, although sometimes interconnected, molecular pathways (e.g., HOG pathway for osmotic stress and proton transport systems for pH regulation) (Hohmann, 2002; Orij et al., 2011). Cluster analysis further revealed distinct phenotypic groupings, with Cluster 2 emerging as the most robust group under combined stress conditions. Strains within this cluster exhibited high metabolic plasticity and stable growth performance across a wide range of environmental conditions, indicating strong homeostatic control mechanisms. Such traits are highly desirable in industrial fermentations, where fluctuations in pH, salinity, and nutrient availability are common (Fleet, 2008; Parapouli et al., 2020). Comparable phenotypic robustness has been described in other non-*Saccharomyces* yeasts selected for starter culture development, highlighting the importance of resilience over maximal growth performance alone (Bonatsou et al., 2017). In contrast, Cluster 3 was characterized by an extended *λ*, which represents a critical limitation in competitive fermentation environments. A prolonged lag phase may compromise the ability of a strain to rapidly colonize the substrate, thereby increasing the risk of dominance by spoilage or undesirable microorganisms (Arroyo-López et al., 2012a,b). This parameter is particularly relevant in table olive fermentation, where microbial succession dynamics are strongly influenced by early colonization events and environmental stressors.

Overall, this multivariate classification provides a robust framework for the selection of candidate starter cultures based on stress resilience rather than solely on growth rate or biomass yield. Such an approach aligns with current trends in food biotechnology, which emphasize the selection of multifunctional and stress-tolerant strains capable of ensuring both process reliability and product quality (Díaz-Muñoz and De Vuyst, 2022; Padilla et al., 2016). The physiological mechanisms underlying tolerance to hypersaline conditions have been extensively studied in model yeast species such as *Saccharomyces cerevisiae* and *Debaryomyces hansenii*. In these microorganisms, osmoadaptation is primarily mediated by activation of High Osmolarity Glycerol (HOG) signaling pathway, involving key components such as Sho1, Pbs2, Hog1, Ste11, Ssk1, Ssk2 and Ypd1. This pathway is triggered by loss of cellular turgor pressure and results in the phosphorylation and activation of the mitogen-activated protein kinase Hog1, ultimately leading to increased intracellular glycerol accumulation via Gpd1 activity (Hohmann, 2002; Tatebayashi et al., 2020). Additionally, oxidative stress responses are activated under high salinity, including the upregulation of antioxidant enzymes such as superoxide dismutase (SOD), catalase (CAT), glutathione peroxidase (GPX), peroxiredoxin (PRX), and glutathione S-transferase (GST), which mitigate the damaging effects of reactive oxygen species generated under stress conditions (Segal-Kischinevzky et al., 2022). Although these mechanisms are well characterized by model yeasts, their presence and regulation in *L. thermotolerans* remain largely unexplored, representing an important area for future research. Given the strong salinity and pH tolerance observed in this study, it is plausible that similar osmoadaptive and stress-response pathways are conserved or functionally analogous in *L. thermotolerans*, although potentially with species-specific regulatory features (Hranilovic et al., 2018; Zhou et al., 2019).

In addition to stress tolerance, the ability of *L. thermotolerans* to produce L-lactic acid has been demonstrated to depend on the regulation of genes involved in the lactate dehydrogenase pathway, controlled by specific transcription factors (Nakatani et al., 2025). However, the extent to which the strains selected in this study can maintain high lactic acid production under the complex and stressful conditions of table olive fermentation remains to be determined. Future studies should therefore evaluate L-lactic acid yields in media that closely mimic the olive matrix, including the presence of inhibitory compounds such as polyphenols (e.g., oleuropein), which are known to exert antimicrobial effects and influence microbial metabolism (Canal et al., 2019; Medina-Pradas and Arroyo-López, 2015). In addition, enzymatic activities should be evaluated, with particular attention to pectolytic activity due to its relevance in pulp softening and potential spoilage during the early stages of fermentation. Additionally, other enzymatic activities, such as lipase and esterase, could be assessed in relation to the release of free fatty acids and ester formation, in combination with sensory analysis. Furthermore, validation at pilot scale will be essential to confirm the technological applicability of the selected strains. Controlled fermentation trials using selected *L. thermotolerans* inocula should be conducted to assess not only fermentation kinetics and acidification capacity but also their impact on the sensory profile of the final product. Sensory evaluation panels will be necessary to ensure that desirable organoleptic attributes -such as flavor, aroma, and texture- are preserved or enhanced, and that no undesirable secondary metabolites are produced (Giavalisco et al., 2024; Hurtado et al., 2012; Tufariello et al., 2019).

## 5 Conclusion

In summary, this study provides a systematic evaluation of the growth behavior of *L. thermotolerans* under pH and salinity conditions relevant to Spanish-style table olive processing, demonstrating that its tolerance ranges are compatible with those encountered during industrial fermentation. Moreover, its performance was comparable to that of *W. anomalus* and *C. boidinii*, two yeast species commonly associated with this process. Importantly, these findings highlight the potential biotechnological relevance of selected *L. thermotolerans* strains as starter cultures, based primarily on their tolerance to pH and NaCl stress, although more studies should be carried out in fermentation trials to assess their performance under industrial conditions.

Through a multivariate stratification approach, forty-one strains were identified as promising candidates based on their combined tolerance to salinity and pH stressors. This selection provides a solid foundation for further functional and technological characterization, ultimately paving the way for the development of innovative and improved fermentation strategies in the table olive industry. Such approaches may contribute to current trends toward reducing chemical inputs and improving process control through the use of tailored microbial starters.

## Data Availability

The original contributions presented in the study are included in the article/Supplementary material, further inquiries can be directed to the corresponding author.
