# Supplementary material for: Evaluating Lachancea thermotolerans for table olive fermentation: performance under pH and NaCl stress conditions
Source: Front Microbiol. 2026 May 22;17:1846416. doi: 10.3389/fmicb.2026.1846416 (PMC13236869; doi:10.3389/fmicb.2026.1846416)
Supplement: Supplementary file 1 [file Supplementary_file_1.docx]

Supplementary Material

SUPPLEMENTARY FIGURES


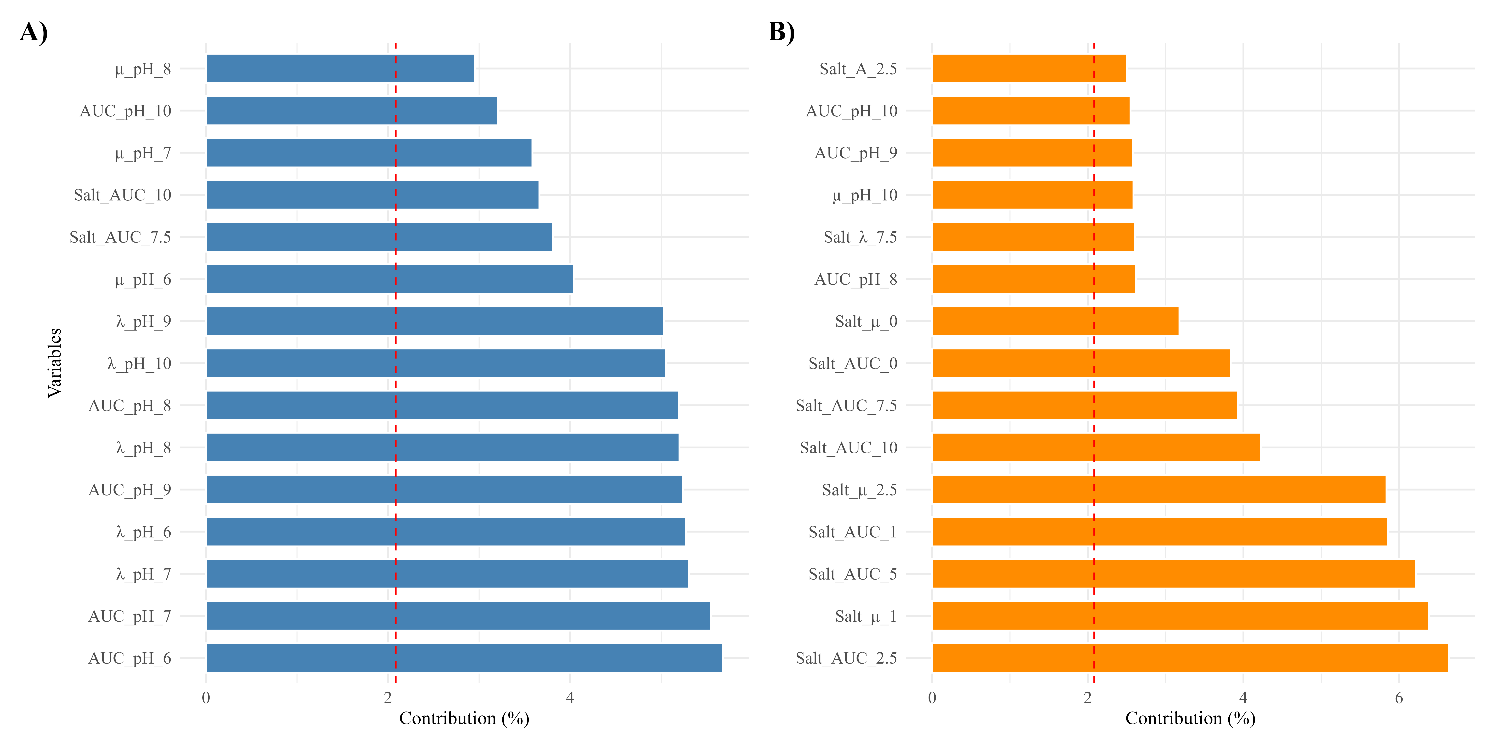


**Supplementary Figure 1.** Variable contributions to the first two principal components (PCA) for *Lachancea* characterization. (A) Contribution of variables to Dimension 1 (PC1). (B) Contribution of variables to Dimension 2 (PC2). The red dashed line indicates the expected average contribution (threshold) if the contributions were uniform. Variables with a contribution above this line are considered significant for the definition of the respective component. Bars are ordered by importance, and color codes differentiate between the two main axes of variance. Bar plots illustrate the top 10 contributions of variables for each principal component.

SUPPLEMENTARY TABLES

**Supplementary Table 1.** List of microorganisms used in the current study.

| **Strain** | **Species** | **Source** | **Geographic origin** |
| --- | --- | --- | --- |
| BMA 8R1 | *L. thermotolerans* | Vineyard soil | Andalusia, Spain |
| BMA 45 | *L. thermotolerans* | Flowers (mix) | Andalusia, Spain |
| BMA 46 | *L. thermotolerans* | Flowers (mix) | Andalusia, Spain |
| BMA 48 | *L. thermotolerans* | Insect | Extremadura, Spain |
| BMA 50 | *L. thermotolerans* | Orchard | Extremadura, Spain |
| BMA 54 | *L. thermotolerans* | Flowers (mix) | Extremadura, Spain |
| BMA 56 | *L. thermotolerans* | Orchard | Extremadura, Spain |
| BMA 57 | *L. thermotolerans* | Orchard | Extremadura, Spain |
| BMA 60 | *L. thermotolerans* | Flowers (mix) | Extremadura, Spain |
| BMA 61 | *L. thermotolerans* | Orchard | Extremadura, Spain |
| BMA 63 | *L. thermotolerans* | Orchard | Extremadura, Spain |
| BMA 64 | *L. thermotolerans* | Flowers (mix) | Extremadura, Spain |
| BMA 65 | *L. thermotolerans* | Flowers (mix) | Extremadura, Spain |
| BMA 66 | *L. thermotolerans* | Orchard | Extremadura, Spain |
| BMA 70 | *L. thermotolerans* | Orchard | Extremadura, Spain |
| BMA 71 | *L. thermotolerans* | Flowers (mix) | Extremadura, Spain |
| BMA 72 | *L. thermotolerans* | Flowers (mix) | Extremadura, Spain |
| BMA 122 | *L. thermotolerans* | Orchard | Andalusia, Spain |
| BMA 123 | *L. thermotolerans* | Orchard | Andalusia, Spain |
| BMA 124 | *L. thermotolerans* | Orchard | Andalusia, Spain |
| BMA 125 | *L. thermotolerans* | Vineyard soil | Andalusia, Spain |
| BMA 127 | *L. thermotolerans* | Orchard | Andalusia, Spain |
| BMA 147 | *L. thermotolerans* | Orchard | Andalusia, Spain |
| BMA 149 | *L. thermotolerans* | Orchard | Andalusia, Spain |
| BMA 150 | *L. thermotolerans* | Orchard | Andalusia, Spain |
| BMA 151 | *L. thermotolerans* | Orchard | Andalusia, Spain |
| BMA 152 | *L. thermotolerans* | Orchard | Andalusia, Spain |
| BMA 153 | *L. thermotolerans* | Orchard | Andalusia, Spain |
| BMA 154 | *L. thermotolerans* | Orchard | Andalusia, Spain |
| BMA 155 | *L. thermotolerans* | Orchard | Andalusia, Spain |
| BMA 156 | *L. thermotolerans* | Orchard | Andalusia, Spain |
| BMA 157 | *L. thermotolerans* | Orchard | Andalusia, Spain |
| BMA 158 | *L. thermotolerans* | Orchard | Andalusia, Spain |
| BMA 166 | *L. thermotolerans* | Insect | Andalusia, Spain |
| BMA 167 | *L. thermotolerans* | Orchard | Andalusia, Spain |
| BMA 179 | *L. thermotolerans* | Vineyard soil | Extremadura, Spain |
| BMA 180 | *L. thermotolerans* | Vineyard soil | Extremadura, Spain |
| BMA 181 | *L. thermotolerans* | Vineyard soil | Extremadura, Spain |
| BMA 182 | *L. thermotolerans* | Vineyard soil | Extremadura, Spain |
| BMA 183 | *L. thermotolerans* | Vineyard soil | Extremadura, Spain |
| BMA 184 | *L. thermotolerans* | Vineyard soil | Extremadura, Spain |
| BMA 185 | *L. thermotolerans* | Vineyard soil | Extremadura, Spain |
| BMA 186 | *L. thermotolerans* | Vineyard soil | Extremadura, Spain |
| BMA 188 | *L. thermotolerans* | Vineyard soil | Extremadura, Spain |
| BMA 189 | *L. thermotolerans* | Orchard | Andalusia, Spain |
| BMA 190 | *L. thermotolerans* | Flowers (mix) | Andalusia, Spain |
| BMA 191 | *L. thermotolerans* | Insect | Andalusia, Spain |
| BMA 192 | *L. thermotolerans* | Insect | Andalusia, Spain |
| BMA 193 | *L. thermotolerans* | Insect | Andalusia, Spain |
| BMA 194 | *L. thermotolerans* | Insect | Andalusia, Spain |
| BMA 196 | *L. thermotolerans* | Insect | Andalusia, Spain |
| BMA 205 | *L. thermotolerans* | Insect | Andalusia, Spain |
| BMA 206 | *L. thermotolerans* | Orchard | Andalusia, Spain |
| BMA 207 | *L. thermotolerans* | Orchard | Andalusia, Spain |
| BMA 208 | *L. thermotolerans* | Insect | Andalusia, Spain |
| BMA 212 | *L. thermotolerans* | Vineyard soil | Andalusia, Spain |
| BMA 214 | *L. thermotolerans* | Flowers (mix) | Andalusia, Spain |
| BMA 219 | *L. thermotolerans* | Insect | Andalusia, Spain |
| BMA 221 | *L. thermotolerans* | Orchard | Andalusia, Spain |
| BMA 222 | *L. thermotolerans* | Orchard | Andalusia, Spain |
| BMA 223 | *L. thermotolerans* | Insect | Andalusia, Spain |
| BMA 224 | *L. thermotolerans* | Flowers (mix) | Andalusia, Spain |
| BMA 225 | *L. thermotolerans* | Orchard | Andalusia, Spain |
| BMA 228 | *L. thermotolerans* | Flowers (mix) | Andalusia, Spain |
| BMA 298 | *L. thermotolerans* | Orchard | Extremadura, Spain |
| BMA 339 | *L. thermotolerans* | Orchard | Extremadura, Spain |
| BMA 17 | *C. boidinii* | Directly brined table olives fermentation | Andalusia, Spain |
| BMA 11 | *W. anomalus* | Spanish-style table olive fermentation | Andalusia, Spain |

**Supplementary Table 2.** Gompertz kinetic parameters in pH conditions. Values represent the mean of replicates ± standard deviation (σ). *A*: maximum asymptotic growth (log₁₀ CFU/g); *μ*: maximum specific growth rate (h⁻¹); *λ*: lag phase duration (h); *AUC*: area under the growth curve; R²(1/2): coefficients of determination for the first and second experimental replicates of the model fit. The effect of pH was evaluated for each strain under controlled conditions.

| Strain | pH | | A±σ | μ±σ | λ±σ | AUC±σ | R²(1/2) |
| --- | --- | --- | --- | --- | --- | --- | --- |
| BMA 122 | 6 | | 1.34±0.014 | 0.24±0.006 | 8.32±0.567 | 91.6±1.78 | 0.999/1 |
| BMA 122 | 7 | | 1.33±0.004 | 0.23±0.006 | 8.07±0.067 | 91.5±0.47 | 0.999/0.999 |
| BMA 122 | 8 | | 1.31±0 | 0.19±0.002 | 7.79±0.032 | 89.4±0.11 | 0.998/0.999 |
| BMA 122 | 9 | | 1.27±0.005 | 0.18±0.012 | 7.59±0.081 | 86.8±0.6 | 0.997/0.998 |
| BMA 122 | 10 | | 1.17±0.024 | 0.2±0.014 | 8.64±0.175 | 79.2±1.45 | 0.991/0.99 |
| BMA 123 | 6 | | 1.35±0.017 | 0.17±0 | 10.05±0.04 | 88.1±1.06 | 0.996/0.995 |
| BMA 123 | 7 | | 1.23±0.005 | 0.16±0 | 9.46±0.759 | 81.4±0.65 | 0.998/0.996 |
| BMA 123 | 8 | | 1.35±0.012 | 0.13±0.008 | 8.36±0.551 | 88.2±0.55 | 0.999/0.996 |
| BMA 123 | 9 | | 1.31±0.011 | 0.12±0.016 | 8.05±0.536 | 86.2±1.17 | 0.999/0.998 |
| BMA 123 | 10 | | 1.17±0.008 | 0.19±0.005 | 8.69±0.131 | 79.2±0.47 | 0.996/0.996 |
| BMA 124 | 6 | | 1.34±0.025 | 0.26±0.003 | 8.47±0.342 | 91.6±2.17 | 0.999/0.999 |
| BMA 124 | 7 | | 1.32±0.018 | 0.25±0.002 | 8.62±0.343 | 90.2±0.72 | 1/0.999 |
| BMA 124 | 8 | | 1.32±0.013 | 0.24±0.013 | 8.4±0.032 | 90.1±1.02 | 0.999/0.999 |
| BMA 124 | 9 | | 1.27±0.018 | 0.22±0.009 | 8.68±0.198 | 86.1±1.59 | 0.999/0.999 |
| BMA 124 | 10 | | 1.15±0.007 | 0.23±0.005 | 9.17±0.314 | 77.9±0.73 | 0.992/0.993 |
| BMA 125 | 6 | | 1.25±0.034 | 0.09±0.007 | 5.1±0.049 | 83.9±2.77 | 0.998/0.999 |
| BMA 125 | 7 | | 1.23±0.032 | 0.1±0.012 | 4.52±0.28 | 84±3.37 | 0.999/0.998 |
| BMA 125 | 8 | | 1.24±0.026 | 0.11±0.011 | 4.75±0.183 | 84.9±2.71 | 0.999/0.999 |
| BMA 125 | 9 | | 1.22±0.022 | 0.12±0.009 | 5.48±0.19 | 84±2.16 | 0.999/0.999 |
| BMA 125 | 10 | | 1.12±0.017 | 0.12±0.005 | 6.28±0.324 | 76.1±1.69 | 0.998/0.997 |
| BMA 127 | 6 | | 1.29±0.01 | 0.19±0.017 | 7.49±0.187 | 88.7±1.37 | 0.996/0.995 |
| BMA 127 | 7 | | 1.3±0.003 | 0.22±0.014 | 7.75±0.371 | 89.7±0.01 | 0.995/0.995 |
| BMA 127 | 8 | | 1.3±0.005 | 0.27±0.019 | 8.92±0.248 | 88.5±0.24 | 0.996/0.997 |
| BMA 127 | 9 | | 1.27±0.013 | 0.28±0.004 | 9.46±0.127 | 85.9±0.63 | 0.998/0.998 |
| BMA 127 | 10 | | 1.14±0.01 | 0.21±0.005 | 9.91±0.47 | 76.2±0.02 | 0.995/0.994 |
| BMA 147 | 6 | | 1.36±0.008 | 0.16±0.004 | 10.07±0.086 | 88.3±0.46 | 0.999/0.999 |
| BMA 147 | 7 | | 1.18±0.005 | 0.15±0.007 | 9.72±0.003 | 77.7±0.06 | 0.997/0.999 |
| BMA 147 | 8 | | 1.33±0.008 | 0.11±0.007 | 8.5±0.581 | 85.7±0.29 | 0.998/0.998 |
| BMA 147 | 9 | | 1.31±0.012 | 0.09±0.005 | 7.69±0.031 | 83.8±0.22 | 0.997/0.997 |
| BMA 147 | 10 | | 1.23±0.112 | 0.12±0.046 | 8.49±0.942 | 80.2±4.88 | 0.997/0.996 |
| BMA 149 | 6 | | 1.35±0 | 0.18±0.017 | 10.38±0.976 | 87.9±0.71 | 0.998/0.996 |
| BMA 149 | 7 | | 1.23±0.01 | 0.18±0.003 | 9.82±0.978 | 81.6±0.51 | 0.998/0.995 |
| BMA 149 | 8 | | 1.33±0.011 | 0.13±0.015 | 9.63±1.126 | 86±0.04 | 0.998/0.998 |
| BMA 149 | 9 | | 1.3±0.005 | 0.12±0.007 | 8.48±0.468 | 84.2±0.15 | 0.998/0.998 |
| BMA 149 | 10 | | 1.15±0 | 0.2±0.003 | 9.41±0.272 | 77.6±0.29 | 0.996/0.996 |
| BMA 150 | 6 | | 1.28±0.099 | 0.16±0.011 | 22.4±3.931 | 67.8±10.21 | 0.998/0.997 |
| BMA 150 | 7 | | 1.08±0.047 | 0.19±0.003 | 15.56±1.263 | 66±1.32 | 0.999/1 |
| BMA 150 | 8 | | 1.33±0.018 | 0.11±0.001 | 14.41±2.099 | 77.5±1.72 | 0.995/0.998 |
| BMA 150 | 9 | | 1.3±0.004 | 0.1±0.001 | 13.45±1.313 | 77±2.03 | 0.996/0.996 |
| BMA 150 | 10 | | 1.19±0.01 | 0.14±0.004 | 14.96±2.449 | 71.7±3.31 | 0.997/0.993 |
| BMA 151 | 6 | | 1.33±0.003 | 0.16±0.008 | 10.52±0.145 | 86.4±0.07 | 0.998/0.999 |
| BMA 151 | 7 | | 1.33±0 | 0.16±0 | 9.88±0 | 86.9±0 | 0.999/0.999 |
| BMA 151 | 8 | | 1.33±0.002 | 0.15±0.005 | 9.36±0.218 | 87.4±0.16 | 0.999/0.998 |
| BMA 151 | 9 | | 1.29±0.003 | 0.15±0.009 | 9.02±0.133 | 84.9±0.05 | 0.997/0.998 |
| BMA 151 | 10 | | 1.14±0.007 | 0.19±0 | 9.8±0.041 | 76.1±0.52 | 0.994/0.993 |
| BMA 152 | 6 | | 1.35±0.001 | 0.17±0.012 | 10.8±0.096 | 87±0.52 | 0.998/0.998 |
| BMA 152 | 7 | | 1.35±0 | 0.16±0 | 10.16±0 | 87.4±0 | 0.999/0.999 |
| BMA 152 | 8 | | 1.34±0.005 | 0.15±0.008 | 9.63±0.058 | 87.8±0.57 | 0.999/0.999 |
| BMA 152 | 9 | | 1.29±0.009 | 0.15±0.008 | 9.44±0.093 | 84.9±0.79 | 0.998/0.998 |
| BMA 152 | 10 | | 1.16±0.001 | 0.19±0.008 | 9.79±0.025 | 77.2±0.22 | 0.997/0.997 |
| BMA 153 | 6 | | 1.35±0.003 | 0.18±0.018 | 10.88±0.249 | 87.3±0.4 | 0.999/0.997 |
| BMA 153 | 7 | | 1.34±0 | 0.16±0 | 10.31±0 | 86.8±0 | 0.999/0.999 |
| BMA 153 | 8 | | 1.33±0.009 | 0.14±0.008 | 9.77±0.266 | 86.3±0.59 | 0.999/0.999 |
| BMA 153 | 9 | | 1.29±0.007 | 0.14±0.019 | 9.78±0.354 | 83.8±0.89 | 0.998/0.999 |
| BMA 153 | 10 | | 1.15±0.017 | 0.21±0.008 | 10.95±0.075 | 75.5±1.12 | 0.996/0.997 |
| BMA 154 | 6 | | 1.34±0.005 | 0.16±0.001 | 11.49±0.528 | 85.6±0.48 | 0.997/0.999 |
| BMA 154 | 7 | | 1.34±0 | 0.14±0 | 10.52±0 | 85.3±0 | 0.999/0.999 |
| BMA 154 | 8 | | 1.33±0.015 | 0.12±0.003 | 9.65±0.066 | 85.1±1.03 | 0.998/0.998 |
| BMA 154 | 9 | | 1.29±0.013 | 0.11±0.002 | 9.49±0.148 | 82±0.76 | 0.998/0.997 |
| BMA 154 | 10 | | 1.15±0 | 0.14±0.002 | 10.37±0.825 | 74.3±0.88 | 0.995/0.998 |
| BMA 155 | 6 | | 1.35±0.006 | 0.18±0.007 | 11.31±0.022 | 86.7±0.18 | 0.998/0.998 |
| BMA 155 | 7 | | 1.34±0 | 0.16±0 | 10.5±0 | 87±0 | 0.999/0.999 |
| BMA 155 | 8 | | 1.34±0.009 | 0.15±0 | 9.86±0.418 | 87.4±0.04 | 0.999/0.999 |
| BMA 155 | 9 | | 1.29±0.002 | 0.15±0.02 | 9.26±0.186 | 84.5±0.74 | 0.999/0.998 |
| BMA 155 | 10 | | 1.16±0.007 | 0.21±0.011 | 10.06±0.47 | 77.5±0.78 | 0.998/0.997 |
| BMA 156 | 6 | | 1.34±0.003 | 0.2±0.008 | 11.43±0.082 | 87±0.3 | 0.996/0.998 |
| BMA 156 | 7 | | 1.34±0 | 0.18±0 | 10.75±0 | 86.9±0 | 0.998/0.998 |
| BMA 156 | 8 | | 1.34±0.005 | 0.16±0.001 | 10.14±0.008 | 86.8±0.32 | 0.999/0.999 |
| BMA 156 | 9 | | 1.29±0.002 | 0.17±0.005 | 10.09±0.045 | 84.2±0.01 | 0.999/0.999 |
| BMA 156 | 10 | | 1.23±0.102 | 0.2±0.045 | 10.29±0.309 | 80.9±5.66 | 0.999/0.996 |
| BMA 157 | 6 | | 1.34±0.001 | 0.18±0.008 | 11.2±1.086 | 86.4±1.14 | 0.997/0.997 |
| BMA 157 | 7 | | 1.34±0 | 0.16±0 | 10.44±0 | 86.6±0 | 0.998/0.998 |
| BMA 157 | 8 | | 1.34±0.01 | 0.14±0.002 | 9.86±0.69 | 86.8±0.26 | 0.999/0.997 |
| BMA 157 | 9 | | 1.3±0.01 | 0.13±0.003 | 8.88±0.114 | 84.9±0.58 | 0.998/0.998 |
| BMA 157 | 10 | | 1.17±0.022 | 0.21±0.011 | 9.9±0.087 | 78.1±1.71 | 0.997/0.994 |
| BMA 158 | 6 | | 1.33±0.009 | 0.2±0.008 | 11.81±0.225 | 85.8±0.46 | 0.995/0.995 |
| BMA 158 | 7 | | 1.35±0 | 0.17±0 | 11.02±0 | 86.7±0 | 0.997/0.997 |
| BMA 158 | 8 | | 1.36±0.02 | 0.15±0.022 | 10.32±0.126 | 87.5±2.1 | 0.997/0.996 |
| BMA 158 | 9 | | 1.33±0.027 | 0.13±0.002 | 9.52±0.366 | 85.7±1.93 | 0.996/0.994 |
| BMA 158 | 10 | | 1.22±0.038 | 0.18±0.016 | 9.87±0.255 | 80.7±2.3 | 0.992/0.981 |
| BMA 166 | 6 | | 1.34±0.01 | 0.22±0.029 | 10.97±0.321 | 87.4±0.82 | 0.998/0.999 |
| BMA 166 | 7 | | 1.33±0 | 0.21±0 | 10.49±0 | 87.6±0 | 0.998/0.998 |
| BMA 166 | 8 | | 1.33±0.027 | 0.2±0.003 | 10.14±1.074 | 87.8±0.32 | 0.997/0.998 |
| BMA 166 | 9 | | 1.3±0.004 | 0.18±0.004 | 9.67±0.306 | 86.2±0.28 | 0.998/0.998 |
| BMA 166 | 10 | | 1.16±0.009 | 0.17±0.01 | 10.19±0.066 | 76.2±0.93 | 0.996/0.996 |
| BMA 167 | 6 | | 1.33±0.048 | 0.23±0.011 | 10.94±0.517 | 87.4±3.91 | 1/0.999 |
| BMA 167 | 7 | | 1.34±0 | 0.21±0 | 10.54±0 | 88.3±0 | 0.999/0.999 |
| BMA 167 | 8 | | 1.35±0.019 | 0.2±0.004 | 10.17±0.463 | 89.1±0.63 | 0.998/0.997 |
| BMA 167 | 9 | | 1.31±0.012 | 0.2±0.004 | 10.15±0.098 | 86.4±0.74 | 0.998/0.998 |
| BMA 167 | 10 | | 1.17±0.009 | 0.18±0.007 | 9.87±0.057 | 77.7±0.68 | 0.995/0.997 |
| BMA 179 | 6 | | 1.34±0.009 | 0.18±0.02 | 9.82±0.201 | 88±0.19 | 0.998/0.999 |
| BMA 179 | 7 | | 1.34±0 | 0.15±0 | 9.09±0 | 88.2±0 | 0.999/0.999 |
| BMA 179 | 8 | | 1.34±0.006 | 0.13±0.001 | 8.39±0.011 | 88.3±0.41 | 0.999/0.998 |
| BMA 179 | 9 | | 1.29±0.004 | 0.15±0.03 | 8.6±0.366 | 85.7±0.62 | 0.998/0.998 |
| BMA 179 | 10 | | 1.16±0.013 | 0.19±0.01 | 8.85±0.032 | 78.3±0.61 | 0.996/0.997 |
| BMA 180 | 6 | | 1.33±0.006 | 0.14±0.011 | 9.34±0.552 | 86.7±0.17 | 0.999/0.999 |
| BMA 180 | 7 | | 1.34±0 | 0.11±0 | 8.34±0 | 86.6±0 | 0.999/0.999 |
| BMA 180 | 8 | | 1.34±0.008 | 0.1±0.007 | 7.39±0.195 | 86.5±0.98 | 0.997/0.998 |
| BMA 180 | 9 | | 1.29±0.002 | 0.09±0.006 | 7.36±0.494 | 83.4±0.09 | 0.997/0.998 |
| BMA 180 | 10 | | 1.16±0.017 | 0.12±0.019 | 7.97±0.045 | 76.8±0.07 | 0.996/0.997 |
| BMA 181 | 6 | | 1.3±0.006 | 0.22±0.025 | 8.76±0.125 | 88.3±1.01 | 0.998/0.999 |
| BMA 181 | 7 | | 1.3±0 | 0.22±0 | 8.35±0 | 88.5±0 | 0.999/0.999 |
| BMA 181 | 8 | | 1.29±0.009 | 0.23±0.01 | 8.12±0.092 | 88.6±0.54 | 0.999/0.999 |
| BMA 181 | 9 | | 1.27±0.009 | 0.25±0.009 | 8.27±0.013 | 87±0.42 | 0.998/0.998 |
| BMA 181 | 10 | | 1.08±0.028 | 0.25±0.025 | 8.53±0.403 | 74.4±1.69 | 0.995/0.995 |
| BMA 182 | 6 | | 1.31±0.017 | 0.15±0.028 | 10.42±0.735 | 84.6±0.65 | 0.999/0.999 |
| BMA 182 | 7 | | 1.33±0 | 0.12±0 | 9.42±0 | 85.1±0 | 0.999/0.999 |
| BMA 182 | 8 | | 1.34±0.011 | 0.1±0.019 | 8.43±0.71 | 85.6±1.56 | 0.997/0.998 |
| BMA 182 | 9 | | 1.3±0.004 | 0.11±0.004 | 8.64±0.517 | 83.2±0.07 | 0.999/0.999 |
| BMA 182 | 10 | | 1.16±0.002 | 0.13±0.019 | 8.67±0.163 | 76.7±0.8 | 0.997/0.997 |
| BMA 183 | 6 | | 1.31±0.023 | 0.15±0.008 | 10.27±0.03 | 84.5±1.03 | 0.998/0.999 |
| BMA 183 | 7 | | 1.32±0 | 0.12±0 | 9.33±0 | 85.2±0 | 0.999/0.999 |
| BMA 183 | 8 | | 1.34±0.01 | 0.11±0.001 | 8.45±0.218 | 85.9±0.18 | 0.999/0.999 |
| BMA 183 | 9 | | 1.29±0.006 | 0.09±0.005 | 8.4±0.283 | 82.2±0.16 | 0.998/0.998 |
| BMA 183 | 10 | | 1.24±0.098 | 0.12±0.035 | 8.57±0.724 | 80.6±4.13 | 0.998/0.996 |
| BMA 184 | 6 | | 1.32±0.017 | 0.15±0.015 | 10.35±0.29 | 85.4±1.29 | 0.995/0.999 |
| BMA 184 | 7 | | 1.34±0 | 0.13±0 | 9.65±0 | 86.1±0 | 0.999/0.999 |
| BMA 184 | 8 | | 1.35±0 | 0.11±0.002 | 9.05±0.098 | 86.7±0.08 | 0.998/0.999 |
| BMA 184 | 9 | | 1.3±0.009 | 0.09±0.006 | 7.96±0.599 | 82.5±0.59 | 0.997/0.996 |
| BMA 184 | 10 | | 1.16±0.031 | 0.16±0.011 | 8.94±0.048 | 77.3±1.65 | 0.997/0.993 |
| BMA 185 | 6 | | 1.31±0.004 | 0.2±0.009 | 12.15±0.166 | 83.6±0.24 | 0.993/0.995 |
| BMA 185 | 7 | | 1.32±0 | 0.16±0 | 11.48±0 | 84.1±0 | 0.997/0.997 |
| BMA 185 | 8 | | 1.34±0.007 | 0.13±0.013 | 10.9±0.428 | 84.5±0.61 | 0.997/0.996 |
| BMA 185 | 9 | | 1.31±0.022 | 0.11±0.009 | 10.15±0.293 | 82.7±0.87 | 0.997/0.996 |
| BMA 185 | 10 | | 1.2±0.048 | 0.15±0 | 9.54±0.065 | 79.1±2.88 | 0.991/0.986 |
| BMA 186 | 6 | | 1.27±0.021 | 0.14±0.002 | 7.93±0.101 | 85±1.26 | 0.998/0.999 |
| BMA 186 | 7 | | 1.28±0 | 0.13±0 | 7.64±0 | 85±0 | 0.999/0.999 |
| BMA 186 | 8 | | 1.29±0.021 | 0.12±0.024 | 7.35±0.584 | 85.1±2.26 | 0.998/0.999 |
| BMA 186 | 9 | | 1.27±0.007 | 0.11±0.023 | 7.63±0.848 | 83.7±1.06 | 0.999/0.999 |
| BMA 186 | 10 | | 1.08±0.027 | 0.13±0.027 | 8.23±0.116 | 72.2±2.75 | 0.997/0.997 |
| BMA 188 | 6 | | 1.37±0.029 | 0.1±0.002 | 23.46±1.04 | 66.4±2.32 | 0.996/0.994 |
| BMA 188 | 7 | | 1.36±0 | 0.1±0 | 22.61±0 | 67±0 | 0.997/0.997 |
| BMA 188 | 8 | | 1.35±0.059 | 0.1±0 | 22.01±0.369 | 67.6±2.93 | 0.999/0.996 |
| BMA 188 | 9 | | 1.33±0.025 | 0.1±0.002 | 21.17±0.442 | 68.1±1.86 | 0.998/0.998 |
| BMA 188 | 10 | | 1.17±0.024 | 0.1±0 | 21.83±0.321 | 60.5±1.45 | 0.995/0.996 |
| BMA 189 | 6 | | 1.36±0.032 | 0.09±0 | 25.55±0.252 | 61.6±1.58 | 0.997/0.994 |
| BMA 189 | 7 | | 1.36±0 | 0.09±0 | 24.63±0 | 62.7±0 | 0.997/0.997 |
| BMA 189 | 8 | | 1.36±0.032 | 0.09±0.002 | 23.9±0.069 | 63.8±1.04 | 0.998/0.997 |
| BMA 189 | 9 | | 1.31±0.02 | 0.09±0 | 23.53±0.864 | 62.6±1.97 | 0.999/0.998 |
| BMA 189 | 10 | | 1.15±0 | 0.08±0 | 23.49±0 | 55.5±0 | 0.996/0.996 |
| BMA 190 | 6 | | 1.34±0.014 | 0.11±0.002 | 25.58±0.24 | 63.1±0.73 | 0.999/0.999 |
| BMA 190 | 7 | | 1.34±0 | 0.11±0 | 25.28±0 | 63.6±0 | 0.998/0.998 |
| BMA 190 | 8 | | 1.35±0.005 | 0.11±0.002 | 24.98±0.253 | 64.2±0.02 | 0.998/0.998 |
| BMA 190 | 9 | | 1.28±0.027 | 0.1±0.011 | 25.27±0.723 | 60.8±1.11 | 0.998/0.998 |
| BMA 190 | 10 | | 1.15±0.012 | 0.11±0.012 | 25.8±0.71 | 55.3±0.47 | 0.997/0.999 |
| BMA 191 | 6 | | 1.42±0.011 | 0.09±0.003 | 22.23±0.358 | 69.5±0.49 | 0.999/0.997 |
| BMA 191 | 7 | | 1.41±0 | 0.09±0 | 22.2±0 | 68.8±0 | 0.999/0.999 |
| BMA 191 | 8 | | 1.4±0.011 | 0.09±0.003 | 22.18±0.203 | 68.2±1.19 | 0.999/0.999 |
| BMA 191 | 9 | | 1.34±0 | 0.08±0 | 22.62±0 | 64.5±0 | 0.999/0.999 |
| BMA 191 | 10 | | 1.22±0.002 | 0.1±0.012 | 24.7±0.092 | 58.6±0.82 | 0.997/0.997 |
| BMA 192 | 6 | | 1.42±0.018 | 0.09±0.003 | 23.18±0.152 | 67.3±0.1 | 0.999/0.997 |
| BMA 192 | 7 | | 1.41±0 | 0.09±0 | 23.09±0 | 67.2±0 | 0.999/0.999 |
| BMA 192 | 8 | | 1.4±0.022 | 0.09±0.012 | 23.09±0.183 | 67.1±0.51 | 0.999/0.998 |
| BMA 192 | 9 | | 1.34±0.015 | 0.09±0.003 | 23.48±0.649 | 64.6±1.13 | 0.999/0.999 |
| BMA 192 | 10 | | 1.19±0.015 | 0.1±0.005 | 25.26±0.319 | 57.4±0.12 | 0.997/0.998 |
| BMA 193 | 6 | | 1.41±0.014 | 0.08±0.003 | 22.36±0.036 | 67.4±0.02 | 1/0.997 |
| BMA 193 | 7 | | 1.4±0 | 0.08±0 | 22.16±0 | 67.5±0 | 0.999/0.999 |
| BMA 193 | 8 | | 1.4±0.01 | 0.08±0.003 | 22±0.009 | 67.5±0.04 | 0.999/0.999 |
| BMA 193 | 9 | | 1.35±0.017 | 0.09±0.002 | 21.97±0.246 | 65.9±1.3 | 0.999/0.999 |
| BMA 193 | 10 | | 1.27±0.124 | 0.09±0.008 | 22.89±1.297 | 62±5.78 | 0.998/0.998 |
| BMA 194 | 6 | | 1.41±0.012 | 0.09±0.014 | 23.55±0.597 | 66.1±2.52 | 1/0.997 |
| BMA 194 | 7 | | 1.4±0 | 0.09±0 | 22.96±0 | 66.7±0 | 0.999/0.999 |
| BMA 194 | 8 | | 1.39±0.005 | 0.09±0.005 | 22.75±0.451 | 67.3±1.46 | 0.999/0.999 |
| BMA 194 | 9 | | 1.34±0.013 | 0.09±0 | 23.07±0.373 | 64.8±0.06 | 0.999/0.999 |
| BMA 194 | 10 | | 1.21±0.016 | 0.11±0.005 | 24.36±0.066 | 59.2±1.12 | 0.997/0.998 |
| BMA 196 | 6 | | 1.36±0.018 | 0.09±0.01 | 21.67±0.235 | 67.8±0.82 | 1/1 |
| BMA 196 | 7 | | 1.36±0 | 0.09±0 | 20.95±0 | 68.7±0 | 1/1 |
| BMA 196 | 8 | | 1.35±0.008 | 0.1±0.006 | 20.47±0.194 | 69.5±0.05 | 1/1 |
| BMA 196 | 9 | | 1.32±0.012 | 0.1±0.006 | 20.8±0.215 | 68±0.85 | 1/1 |
| BMA 196 | 10 | | 1.19±0.013 | 0.11±0.001 | 21.85±0.626 | 61.4±1.41 | 0.998/0.999 |
| BMA 205 | 6 | | 1.37±0.012 | 0.09±0.003 | 21.21±0.187 | 68.8±0.62 | 0.999/0.999 |
| BMA 205 | 7 | | 1.37±0 | 0.09±0 | 20.47±0 | 70±0 | 0.999/0.999 |
| BMA 205 | 8 | | 1.37±0 | 0.1±0.003 | 19.94±0.503 | 71±1.05 | 1/0.999 |
| BMA 205 | 9 | | 1.33±0.005 | 0.11±0.001 | 20.13±0.431 | 70±0.94 | 1/0.999 |
| BMA 205 | 10 | | 1.19±0.019 | 0.11±0.003 | 20.46±0.006 | 63.1±1.09 | 0.999/0.999 |
| BMA 206 | 6 | | 1.13±0.031 | 0.07±0.002 | 9.4±0.421 | 70±1.41 | 0.998/0.998 |
| BMA 206 | 7 | | 1.16±0 | 0.07±0 | 9.57±0 | 71.1±0 | 0.999/0.999 |
| BMA 206 | 8 | | 1.19±0.106 | 0.08±0.002 | 10.09±1.935 | 72.1±3.41 | 0.998/0.999 |
| BMA 206 | 9 | | 1.18±0.035 | 0.08±0.007 | 10.33±1.286 | 72.1±1.1 | 0.998/0.998 |
| BMA 206 | 10 | | 1±0.019 | 0.07±0 | 9.11±0.138 | 62.6±0.96 | 0.996/0.997 |
| BMA 207 | 6 | | 1.24±0.005 | 0.09±0.003 | 10.02±0.728 | 77.3±0.34 | 0.999/0.998 |
| BMA 207 | 7 | | 1.22±0 | 0.09±0 | 9.49±0 | 76.5±0 | 0.999/0.999 |
| BMA 207 | 8 | | 1.21±0.001 | 0.09±0 | 9±0.531 | 75.7±0.62 | 0.999/0.998 |
| BMA 207 | 9 | | 1.21±0.007 | 0.1±0.003 | 10.06±0.132 | 76.4±0.46 | 0.998/0.998 |
| BMA 207 | 10 | | 1.06±0.036 | 0.09±0.004 | 10.28±1.167 | 66.4±0.47 | 0.996/0.995 |
| BMA 208 | 6 | | 1.36±0.015 | 0.12±0.003 | 19.72±0.33 | 73.1±0.02 | 1/0.999 |
| BMA 208 | 7 | | 1.35±0 | 0.12±0 | 19.44±0 | 73.1±0 | 1/1 |
| BMA 208 | 8 | | 1.35±0.001 | 0.12±0.001 | 19.21±0.453 | 73.2±0.62 | 0.999/0.999 |
| BMA 208 | 9 | | 1.32±0.002 | 0.12±0.001 | 18.69±0.011 | 72.6±0.02 | 0.999/0.999 |
| BMA 208 | 10 | | 1.18±0.005 | 0.11±0.001 | 19.93±0.072 | 63.6±0.22 | 0.999/0.999 |
| BMA 212 | 6 | | 1.34±0.001 | 0.11±0.005 | 19.34±0.059 | 71.9±0.33 | 1/1 |
| BMA 212 | 7 | | 1.35±0 | 0.11±0 | 18.82±0 | 72.9±0 | 1/1 |
| BMA 212 | 8 | | 1.35±0.003 | 0.11±0 | 18.42±0.728 | 73.9±1.11 | 1/0.999 |
| BMA 212 | 9 | | 1.31±0.004 | 0.11±0.001 | 18.39±0.394 | 71.8±0.44 | 1/1 |
| BMA 212 | 10 | | 1.19±0.003 | 0.11±0.003 | 20.02±0.517 | 63.8±1 | 1/1 |
| BMA 214 | 6 | | 1.37±0.006 | 0.13±0 | 22.87±0.246 | 69.5±0.09 | 1/0.999 |
| BMA 214 | 7 | | 1.36±0 | 0.13±0 | 22.74±0 | 69.6±0 | 1/1 |
| BMA 214 | 8 | | 1.36±0.002 | 0.13±0.001 | 22.63±0.062 | 69.8±0.04 | 0.999/0.999 |
| BMA 214 | 9 | | 1.32±0.01 | 0.13±0.005 | 22.79±0.133 | 67.5±0.97 | 0.999/0.999 |
| BMA 214 | 10 | | 1.26±0.107 | 0.12±0.002 | 22.95±0.296 | 64.3±5.1 | 0.999/0.998 |
| BMA 219 | 6 | | 1.33±0.019 | 0.1±0.002 | 14.41±0.499 | 76.9±0.5 | 1/0.999 |
| BMA 219 | 7 | | 1.33±0 | 0.1±0 | 13.74±0 | 77.2±0 | 1/1 |
| BMA 219 | 8 | | 1.32±0.019 | 0.1±0 | 13.18±0.795 | 77.6±0.04 | 1/0.999 |
| BMA 219 | 9 | | 1.3±0.002 | 0.1±0.004 | 13.42±0.449 | 76.2±0.93 | 0.999/0.999 |
| BMA 219 | 10 | | 1.16±0.023 | 0.1±0.007 | 13.68±0.884 | 69.1±0.77 | 0.999/0.999 |
| BMA 221 | 6 | | 1.34±0.004 | 0.1±0.006 | 19.35±0.845 | 70.6±1.98 | 0.999/0.999 |
| BMA 221 | 7 | | 1.33±0 | 0.1±0 | 18.45±0 | 71.6±0 | 1/1 |
| BMA 221 | 8 | | 1.33±0.009 | 0.1±0.001 | 17.93±0.152 | 72.6±0.52 | 1/1 |
| BMA 221 | 9 | | 1.3±0 | 0.1±0.005 | 18.08±0.02 | 70.8±0.46 | 1/1 |
| BMA 221 | 10 | | 1.18±0 | 0.11±0.009 | 19.2±0.848 | 63.9±1.68 | 0.999/0.999 |
| BMA 222 | 6 | | 1.29±0.017 | 0.11±0.012 | 7.29±0.038 | 85.2±0.06 | 0.997/0.997 |
| BMA 222 | 7 | | 1.3±0 | 0.14±0 | 8.01±0 | 86.5±0 | 0.998/0.998 |
| BMA 222 | 8 | | 1.31±0.026 | 0.18±0.061 | 8.65±1.857 | 87.9±1.22 | 0.999/1 |
| BMA 222 | 9 | | 1.28±0.025 | 0.24±0.047 | 8±0.302 | 88.2±2.05 | 0.999/0.999 |
| BMA 222 | 10 | | 1.11±0.014 | 0.28±0.015 | 8.84±0.583 | 76.3±1.45 | 0.999/0.999 |
| BMA 223 | 6 | | 1.33±0.032 | 0.23±0.021 | 10.32±0.327 | 88.3±2.84 | 0.999/0.999 |
| BMA 223 | 7 | | 1.34±0 | 0.23±0 | 9.84±0 | 89.7±0 | 0.999/0.999 |
| BMA 223 | 8 | | 1.36±0.002 | 0.24±0.005 | 9.53±0.043 | 91.1±0.18 | 0.999/0.999 |
| BMA 223 | 9 | | 1.31±0.011 | 0.24±0.002 | 9.61±0.147 | 88.2±0.45 | 0.999/0.999 |
| BMA 223 | 10 | | 1.15±0.023 | 0.25±0.005 | 10.03±0.102 | 77.2±1.52 | 0.999/1 |
| BMA 224 | 6 | | 1.31±0.013 | 0.2±0.022 | 10.14±1.037 | 86.5±1.56 | 0.996/0.996 |
| BMA 224 | 7 | | 1.33±0 | 0.19±0 | 9.78±0 | 87.7±0 | 0.996/0.996 |
| BMA 224 | 8 | | 1.34±0.027 | 0.19±0.013 | 9.52±0.057 | 89±1.95 | 0.996/0.996 |
| BMA 224 | 9 | | 1.32±0.019 | 0.24±0.031 | 10.15±0.621 | 87.6±0.97 | 0.997/0.999 |
| BMA 224 | 10 | | 1.1±0.022 | 0.18±0.015 | 9.15±0.802 | 74.2±0.82 | 0.997/0.998 |
| BMA 225 | 6 | | 1.32±0.02 | 0.18±0.017 | 9.28±0.371 | 87.6±1.32 | 0.997/0.997 |
| BMA 225 | 7 | | 1.31±0 | 0.17±0 | 8.82±0 | 87.4±0 | 0.996/0.996 |
| BMA 225 | 8 | | 1.3±0.026 | 0.17±0.016 | 8.4±0.263 | 87.3±1.82 | 0.996/0.996 |
| BMA 225 | 9 | | 1.28±0.007 | 0.17±0.01 | 8.08±0.228 | 86.8±1.06 | 0.995/0.995 |
| BMA 225 | 10 | | 1.13±0.018 | 0.11±0.003 | 7.19±0.687 | 75.7±1.67 | 0.993/0.994 |
| BMA 228 | 6 | | 1.29±0.012 | 0.12±0.01 | 6.4±0.534 | 86.8±0.76 | 0.996/0.997 |
| BMA 228 | 7 | | 1.29±0 | 0.12±0 | 6.41±0 | 86.9±0 | 0.998/0.998 |
| BMA 228 | 8 | | 1.29±0.013 | 0.12±0.01 | 6.63±0.23 | 86.7±1.16 | 0.998/0.999 |
| BMA 228 | 9 | | 1.25±0.007 | 0.11±0 | 6.29±0.156 | 83.4±0.61 | 0.999/0.998 |
| BMA 228 | 10 | | 1.06±0.016 | 0.06±0.006 | 5.18±0.213 | 69.4±2.1 | 0.992/0.993 |
| BMA 298 | 6 | | 1.33±0 | 0.23±0.004 | 9.36±0.122 | 89.2±0.05 | 0.999/0.998 |
| BMA 298 | 7 | | 1.32±0.003 | 0.2±0.005 | 9.05±0.174 | 88.4±0.26 | 0.999/0.999 |
| BMA 298 | 8 | | 1.29±0.007 | 0.14±0.002 | 8.31±0.017 | 85.6±0.34 | 0.998/0.998 |
| BMA 298 | 9 | | 1.26±0 | 0.12±0.02 | 8.02±0.236 | 83±0.89 | 0.998/0.996 |
| BMA 298 | 10 | | 1.22±0.107 | 0.14±0.041 | 8.49±0.933 | 80.7±5.6 | 0.995/0.987 |
| BMA 339 | 6 | | 1.37±0.009 | 0.19±0.018 | 7.84±0.01 | 92.9±1.08 | 0.998/0.999 |
| BMA 339 | 7 | | 1.34±0.003 | 0.21±0.013 | 7.75±0.168 | 91.6±0.74 | 0.997/0.998 |
| BMA 339 | 8 | | 1.32±0.018 | 0.18±0.004 | 7.47±0.001 | 90.1±1.28 | 0.989/0.993 |
| BMA 339 | 9 | | 1.29±0.024 | 0.17±0.004 | 7.51±0.071 | 87.7±1.74 | 0.99/0.988 |
| BMA 339 | 10 | | 1.21±0.012 | 0.2±0.01 | 8.28±0.225 | 82.8±0.71 | 0.991/0.991 |
| BMA 45 | 6 | | 1.34±0.006 | 0.22±0.006 | 9.35±0.232 | 90±0.83 | 0.996/0.996 |
| BMA 45 | 7 | | 1.34±0.007 | 0.25±0.019 | 9.07±0.301 | 91±0.36 | 0.994/0.995 |
| BMA 45 | 8 | | 1.36±0.002 | 0.22±0.011 | 8.72±0.051 | 91.7±0.03 | 0.995/0.996 |
| BMA 45 | 9 | | 1.27±0.008 | 0.22±0.013 | 9.05±0.571 | 86.1±1.01 | 0.998/0.998 |
| BMA 45 | 10 | | 1.16±0.029 | 0.22±0.013 | 8.67±0.092 | 78.8±1.77 | 0.997/0.997 |
| BMA 46 | 6 | | 1.33±0.001 | 0.11±0.002 | 6.46±0.182 | 88.6±0.38 | 0.996/0.997 |
| BMA 46 | 7 | | 1.31±0.011 | 0.09±0 | 5.09±0.287 | 87.2±0.32 | 0.991/0.991 |
| BMA 46 | 8 | | 1.22±0.019 | 0.05±0 | 2.63±0.178 | 78.7±1.27 | 0.966/0.966 |
| BMA 46 | 9 | | 1.19±0.068 | 0.05±0.002 | 1.34±1.032 | 77.1±1.69 | 0.949/0.964 |
| BMA 46 | 10 | | 1.16±0.13 | 0.03±0.003 | 3.08±1.7 | 62.3±3.19 | 0.973/0.979 |
| BMA 48 | 6 | | 1.34±0.025 | 0.16±0.012 | 7.77±0.598 | 90.3±1.23 | 0.997/0.998 |
| BMA 48 | 7 | | 1.36±0.013 | 0.16±0.009 | 7.69±0.239 | 91.7±1.51 | 0.998/0.997 |
| BMA 48 | 8 | | 1.35±0.008 | 0.13±0.005 | 6.59±0.09 | 90.6±0.74 | 0.998/0.997 |
| BMA 48 | 9 | | 1.25±0.019 | 0.12±0.004 | 7.05±0.029 | 83.4±1.47 | 0.996/0.995 |
| BMA 48 | 10 | | 1.08±0.017 | 0.06±0.008 | 4.2±0.134 | 69.9±2.57 | 0.985/0.984 |
| BMA 50 | 6 | | 1.32±0.015 | 0.12±0.009 | 7.8±0.288 | 86.9±1.11 | 0.998/0.997 |
| BMA 50 | 7 | | 1.35±0.005 | 0.13±0.002 | 7.79±0.049 | 89.2±0.5 | 0.998/0.996 |
| BMA 50 | 8 | | 1.33±0.016 | 0.1±0.003 | 7.19±0.163 | 86.7±0.9 | 0.995/0.996 |
| BMA 50 | 9 | | 1.21±0.037 | 0.07±0.006 | 5.97±0.472 | 76.9±2.53 | 0.982/0.99 |
| BMA 50 | 10 | | 0.56±0.068 | 0.11±0.009 | 8.06±0.181 | 38.3±4.69 | 0.997/0.997 |
| BMA 54 | 6 | | 1.34±0.001 | 0.24±0.008 | 8.73±0.34 | 91.4±0.4 | 0.998/0.998 |
| BMA 54 | 7 | | 1.33±0.001 | 0.27±0.033 | 9.05±0.114 | 90.8±0.26 | 0.996/0.997 |
| BMA 54 | 8 | | 1.34±0.005 | 0.26±0.003 | 8.62±0.249 | 91.4±0.69 | 0.994/0.996 |
| BMA 54 | 9 | | 1.26±0.002 | 0.29±0.003 | 9.09±0.008 | 85.9±0.12 | 0.999/0.999 |
| BMA 54 | 10 | | 1.13±0.005 | 0.24±0.008 | 8.78±0.238 | 77.6±0.15 | 0.997/0.996 |
| BMA 56 | 6 | | 1.35±0.013 | 0.2±0.004 | 9.68±0.401 | 89.5±1.25 | 0.997/0.996 |
| BMA 56 | 7 | | 1.38±0.002 | 0.21±0.004 | 9.98±0.106 | 91.5±0.09 | 0.996/0.995 |
| BMA 56 | 8 | | 1.36±0.04 | 0.16±0.022 | 8.96±0.48 | 89.9±2.75 | 0.997/0.997 |
| BMA 56 | 9 | | 1.29±0.005 | 0.16±0.015 | 9.63±0.548 | 84.7±0.22 | 0.998/0.997 |
| BMA 56 | 10 | | 1.1±0.024 | 0.06±0.016 | 6.36±1.384 | 69.3±2.89 | 0.986/0.993 |
| BMA 57 | 6 | | 1.35±0.005 | 0.13±0.003 | 8.29±0.111 | 88.7±0.28 | 0.998/0.999 |
| BMA 57 | 7 | | 1.35±0.001 | 0.11±0.001 | 7.37±0.165 | 88.3±0.08 | 0.996/0.995 |
| BMA 57 | 8 | | 1.35±0.015 | 0.09±0.01 | 6.87±0.573 | 86.7±1.44 | 0.993/0.996 |
| BMA 57 | 9 | | 1.26±0.026 | 0.06±0.006 | 5.53±0.708 | 79.7±1.76 | 0.985/0.987 |
| BMA 57 | 10 | | 1.18±0.02 | 0.08±0.036 | 6.63±1.753 | 76.2±1.36 | 0.987/0.996 |
| BMA 60 | 6 | | 1.35±0.015 | 0.2±0.013 | 10.02±0.444 | 89±1.87 | 0.996/0.997 |
| BMA 60 | 7 | | 1.36±0.017 | 0.22±0.043 | 10.67±0.773 | 89.2±0.95 | 0.998/0.995 |
| BMA 60 | 8 | | 1.36±0.003 | 0.17±0.021 | 10.13±0.914 | 89±0.69 | 0.998/0.997 |
| BMA 60 | 9 | | 1.26±0.01 | 0.14±0.001 | 9.4±0.039 | 82±0.7 | 0.998/0.997 |
| BMA 60 | 10 | | 1.12±0.016 | 0.06±0.003 | 6.83±0.303 | 70±1.77 | 0.99/0.987 |
| BMA 61 | 6 | | 1.37±0.02 | 0.21±0.01 | 6.34±0.579 | 96±0.77 | 0.994/0.995 |
| BMA 61 | 7 | | 1.34±0.009 | 0.21±0.014 | 5.65±0.483 | 94.9±0.32 | 0.994/0.994 |
| BMA 61 | 8 | | 1.28±0.091 | 0.2±0.072 | 4.8±2.521 | 91.4±4.75 | 0.996/0.996 |
| BMA 61 | 9 | | 1.31±0.039 | 0.2±0.03 | 5.78±1.052 | 92.3±1.92 | 0.993/0.993 |
| BMA 61 | 10 | | 1.14±0.084 | 0.13±0.036 | 4.04±2.589 | 80.4±4.14 | 0.991/0.991 |
| BMA 63 | 6 | | 1.36±0.001 | 0.24±0.008 | 9.85±0.245 | 90.6±0.1 | 0.997/0.997 |
| BMA 63 | 7 | | 1.38±0.014 | 0.27±0.014 | 9.88±0.029 | 92.6±1.11 | 0.997/0.997 |
| BMA 63 | 8 | | 1.38±0.005 | 0.23±0.004 | 9.56±0.146 | 92.3±0.42 | 0.997/0.995 |
| BMA 63 | 9 | | 1.31±0.013 | 0.26±0.02 | 9.86±0.04 | 88±1.09 | 0.998/0.998 |
| BMA 63 | 10 | | 1.19±0.07 | 0.17±0.009 | 8.98±0.146 | 79.6±4.84 | 0.996/0.991 |
| BMA 64 | 6 | | 1.37±0.019 | 0.22±0.004 | 9.44±0.146 | 92±1.49 | 0.996/0.996 |
| BMA 64 | 7 | | 1.31±0 | 0.25±0.013 | 8.96±0.386 | 89.1±0.34 | 0.995/0.996 |
| BMA 64 | 8 | | 1.38±0.003 | 0.23±0.004 | 8.62±0.053 | 93.7±0.32 | 0.997/0.995 |
| BMA 64 | 9 | | 1.36±0.006 | 0.23±0.002 | 8.37±0.057 | 92.6±0.36 | 0.996/0.996 |
| BMA 64 | 10 | | 1.19±0 | 0.24±0.026 | 8.69±0.776 | 81.4±0.52 | 0.996/0.995 |
| BMA 65 | 6 | | 1.37±0.016 | 0.22±0.01 | 9.83±0.071 | 91.4±1.13 | 0.996/0.995 |
| BMA 65 | 7 | | 1.31±0.009 | 0.25±0.002 | 9.85±0.06 | 88±0.5 | 0.995/0.995 |
| BMA 65 | 8 | | 1.38±0.012 | 0.24±0.009 | 9.75±0.106 | 92.3±0.81 | 0.997/0.997 |
| BMA 65 | 9 | | 1.35±0.003 | 0.21±0.005 | 9.23±0.159 | 90.9±0.52 | 0.996/0.997 |
| BMA 65 | 10 | | 1.18±0.003 | 0.21±0.002 | 9.22±0.152 | 79.7±0.37 | 0.997/0.998 |
| BMA 66 | 6 | | 1.36±0.003 | 0.22±0.006 | 8.25±0.061 | 92.7±0.42 | 0.998/0.998 |
| BMA 66 | 7 | | 1.34±0.004 | 0.24±0 | 8.22±0.23 | 92.1±0.58 | 0.998/0.998 |
| BMA 66 | 8 | | 1.32±0.001 | 0.23±0.004 | 8.2±0.347 | 90.5±0.33 | 0.998/0.998 |
| BMA 66 | 9 | | 1.28±0.025 | 0.21±0.042 | 8.34±0.232 | 86.9±2.26 | 0.997/0.997 |
| BMA 66 | 10 | | 1.23±0.113 | 0.17±0.004 | 8.49±0.657 | 82.8±8.08 | 0.995/0.993 |
| BMA 70 | 6 | | 1.37±0.017 | 0.23±0.011 | 9.65±0.11 | 91.5±1.19 | 0.998/0.997 |
| BMA 70 | 7 | | 1.3±0.011 | 0.24±0.001 | 9.71±0.198 | 87.5±0.46 | 0.997/0.998 |
| BMA 70 | 8 | | 1.36±0.014 | 0.24±0.001 | 9.59±0.108 | 91.3±0.76 | 0.998/0.998 |
| BMA 70 | 9 | | 1.32±0.007 | 0.23±0.011 | 9.23±0.152 | 89.2±0.46 | 0.999/0.999 |
| BMA 70 | 10 | | 1.17±0.001 | 0.23±0.008 | 9.58±0.313 | 78.8±0.42 | 0.998/0.998 |
| BMA 71 | 6 | | 1.27±0.008 | 0.22±0.035 | 9.77±1.12 | 84.6±1.25 | 0.995/0.997 |
| BMA 71 | 7 | | 1.27±0 | 0.21±0 | 9.25±0 | 85.2±0 | 0.997/0.997 |
| BMA 71 | 8 | | 1.27±0.007 | 0.22±0.015 | 8.93±0.325 | 85.9±0.37 | 0.998/0.998 |
| BMA 71 | 9 | | 1.25±0.002 | 0.26±0.008 | 9.56±0.227 | 84.5±0.06 | 0.998/0.998 |
| BMA 71 | 10 | | 0.9±0.166 | 0.21±0.136 | 8.76±2.972 | 61.1±10.03 | 0.995/0.998 |
| BMA 72 | 6 | | 1.37±0.002 | 0.24±0.005 | 9.78±0.114 | 91.7±0.16 | 0.997/0.997 |
| BMA 72 | 7 | | 1.3±0.007 | 0.24±0.008 | 9.59±0.299 | 87.3±0.69 | 0.996/0.996 |
| BMA 72 | 8 | | 1.35±0.005 | 0.23±0.006 | 9.59±0.019 | 90.7±0.14 | 0.997/0.997 |
| BMA 72 | 9 | | 1.33±0.008 | 0.23±0.014 | 8.99±0.238 | 90±0.45 | 0.998/0.998 |
| BMA 72 | 10 | | 1.18±0.011 | 0.22±0.004 | 9.59±0.938 | 79.6±0.49 | 0.998/0.998 |
| BMA 8R1 | 6 | | 1.3±0.011 | 0.24±0.048 | 3.76±0.341 | 94.9±1.17 | 0.999/0.997 |
| BMA 8R1 | 7 | | 1.3±0.006 | 0.27±0.015 | 4.22±0.392 | 94.6±0.72 | 0.998/0.999 |
| BMA 8R1 | 8 | | 1.3±0.002 | 0.37±0.015 | 5.11±0.018 | 95±0.25 | 0.998/0.997 |
| BMA 8R1 | 9 | | 1.27±0.015 | 0.37±0.014 | 5.39±0.014 | 92.2±0.97 | 0.998/0.997 |
| BMA 8R1 | | 10 | 1.14±0.041 | 0.25±0.016 | 5.57±0.021 | 81.5±2.66 | 0.998/0.991 |
| *Candida boidinii* | | 6 | 2.03±0.007 | 0.19±0.014 | 14.51±0.1 | 120±0.39 | 0.994/0.992 |
| *Candida boidinii* | | 7 | 2.05±0.018 | 0.18±0.003 | 13.95±0.196 | 121.7±0.33 | 0.994/0.997 |
| *Candida boidinii* | | 8 | 2.11±0.038 | 0.16±0.009 | 14.79±0.243 | 121.4±1.58 | 0.997/0.993 |
| *Candida boidinii* | | 9 | 2.15±0.076 | 0.17±0.002 | 16.95±0.02 | 119.8±3.81 | 0.996/0.994 |
| *Candida boidinii* | | 10 | 1.99±0.022 | 0.21±0.005 | 21.4±0.765 | 105.8±2.85 | 0.998/0.998 |
| *Wickerhamomyces anomalus* | | 6 | 2.03±0.022 | 0.14±0.017 | 11.24±0.213 | 121.9±0.67 | 0.992/0.989 |
| *Wickerhamomyces anomalus* | | 7 | 1.96±0.009 | 0.13±0.023 | 8.83±0.205 | 122.6±3.12 | 0.996/0.982 |
| *Wickerhamomyces anomalus* | | 8 | 1.93±0.06 | 0.14±0.014 | 10.59±0.288 | 118±5.32 | 0.993/0.988 |
| *Wickerhamomyces anomalus* | | 9 | 1.86±0.042 | 0.13±0.014 | 9.3±0.8 | 116±2.03 | 0.988/0.986 |
| *Wickerhamomyces anomalus* | | 10 | 1.85±0.028 | 0.12±0.003 | 9.69±0.97 | 113.8±2.87 | 0.997/0.996 |

**Supplementary Table 3.** Statistical Analysis of pH conditions Gompertz non lineal regression: Due to the non-normal distribution of the kinetic parameters (Shapiro-Wilk test, p < 0.05) and heteroscedasticity, the non-parametric Kruskal-Wallis rank sum test was employed to assess global differences across salt concentrations. For pairwise comparisons, the Dunn Post-hoc test was performed with Bonferroni adjustment to control the family-wise error rate. Significance levels are indicated as follows: * (p < 0.05), ** (p < 0.01), *** (p < 0.001), and **** (p < 0.0001)."

| **GLOBAL KRUSKAL-WALLIS TEST SUMMARY** | | | | |
| --- | --- | --- | --- | --- |
| Parameter | N | Chi-sq (H) | df | p-value (raw) |
| *A* | 680 | 314.359390 | 4 | 8.65e-67 |
| *AUC* | 680 | 130.232516 | 4 | 3.47e-27 |
| *λ* | 680 | 13.610585 | 4 | 8.65e-03 |
| *µ* | 680 | 9.202276 | 4 | 5.62e-02 |

| **SIGNIFICANT POST-HOC DUNN TEST COMPARISONS** | | | | | |
| --- | --- | --- | --- | --- | --- |
| Parameter | Comparison | Z-score | p-value (unadj.) | p-value (Bonf.) | Sig. |
| *A* | 6 vs 9 | -7.1303 | 1.001e-12 | 1.001e-11 | ******** |
|  | 6 vs 10 | -14.7449 | 3.317e-49 | 3.317e-48 | ******** |
|  | 7 vs 9 | -5.5234 | 3.324e-08 | 3.324e-07 | ******** |
|  | 7 vs 10 | -13.1381 | 1.993e-39 | 1.993e-38 | ******** |
|  | 8 vs 9 | -6.7593 | 1.386e-11 | 1.386e-10 | ******** |
|  | 8 vs 10 | -14.3739 | 7.544e-47 | 7.544e-46 | ******** |
|  | 9 vs 10 | -7.6146 | 2.645e-14 | 2.645e-13 | ******** |
| *AUC* | 6 vs 9 | -3.2341 | 1.220e-03 | 1.220e-02 | ***** |
|  | 6 vs 10 | -9.3274 | 1.085e-20 | 1.085e-19 | ******** |
|  | 7 vs 9 | -2.8844 | 3.921e-03 | 3.921e-02 | ***** |
|  | 7 vs 10 | -8.9776 | 2.766e-19 | 2.766e-18 | ******** |
|  | 8 vs 9 | -3.3993 | 6.757e-04 | 6.757e-03 | ****** |
|  | 8 vs 10 | -9.4925 | 2.256e-21 | 2.256e-20 | ******** |
|  | 9 vs 10 | -6.0932 | 1.107e-09 | 1.107e-08 | ******** |
| *λ* | 6 vs 9 | -3.2579 | 1.122e-03 | 1.122e-02 | ***** |

**Supplementary Table 4:** Gompertz kinetic parameters in salt conditions. Values represent the mean of replicates ± standard deviation (σ). *A*: maximum asymptotic growth (log₁₀ CFU/g); *μ*: maximum specific growth rate (h⁻¹); *λ*: lag phase duration (h); *AUC*: area under the growth curve; R²(1/2): coefficients of determination for the first and second experimental replicates of the model fit. The effect of salt was evaluated for each strain under controlled conditions except 15 and 20 % of salt and *Candida boidinii* 10 % of salt due to the lack of growth. In these cases, *AUC* was estimated by the integration of extrapolated values.

| Strain | Salt | A±σ | μ±σ | λ±σ | AUC±σ | R²(1/2) |
| --- | --- | --- | --- | --- | --- | --- |
| BMA 122 | 0.0 | 1.34±0.005 | 0.21±0.018 | 9.24±0.087 | 63.2±0.72 | 0.995/0.995 |
| BMA 122 | 1.0 | 1.39±0.006 | 0.19±0.007 | 9.22±0.034 | 64.5±0.42 | 0.993/0.993 |
| BMA 122 | 2.5 | 1.35±0.005 | 0.16±0.006 | 10±0.463 | 61±0.19 | 0.995/0.995 |
| BMA 122 | 5.0 | 1.06±0.056 | 0.06±0.003 | 11.13±0.348 | 41.1±1.84 | 0.998/0.999 |
| BMA 122 | 7.5 | 0.63±0.029 | 0.02±0.001 | 17.75±0.448 | 17.4±0.37 | 0.987/0.987 |
| BMA 122 | 10.0 | 0.2±0.002 | 0±0 | 9.82±0.112 | 4.3±0.13 | 0.973/0.975 |
| BMA 122 | 15.0 | - | - | - | 0.6±0.52 | n/a |
| BMA 122 | 20.0 | - | - | - | 0.4±0.09 | n/a |
| BMA 123 | 0.0 | 1.24±0.106 | 0.22±0.049 | 8.29±0.872 | 59.8±4.61 | 0.997/0.993 |
| BMA 123 | 1.0 | 1.27±0.021 | 0.23±0.002 | 7.89±1.142 | 62.1±0.46 | 0.996/0.995 |
| BMA 123 | 2.5 | 1.26±0.006 | 0.23±0.017 | 9.54±0.477 | 59.6±0.65 | 0.997/0.998 |
| BMA 123 | 5.0 | 1.15±0.002 | 0.09±0.015 | 14.07±1.719 | 44.5±3.39 | 0.992/0.996 |
| BMA 123 | 7.5 | 1.16±0.001 | 0.1±0.001 | 14.34±0.657 | 45.3±0.73 | 0.994/0.993 |
| BMA 123 | 10.0 | 1.15±0.02 | 0.04±0.004 | 16.53±0.295 | 30.8±1.29 | 0.988/0.989 |
| BMA 123 | 15.0 | - | - | - | 4.3±0.83 | n/a |
| BMA 123 | 20.0 | - | - | - | 4.6±0.45 | n/a |
| BMA 124 | 0.0 | 1.3±0.001 | 0.24±0.015 | 8.67±0.405 | 62.6±0.23 | 0.994/0.995 |
| BMA 124 | 1.0 | 1.37±0.003 | 0.17±0.006 | 7.05±0.159 | 66±0.14 | 0.994/0.993 |
| BMA 124 | 2.5 | 1.29±0 | 0.11±0 | 6.89±0.08 | 59.7±0.06 | 0.997/0.995 |
| BMA 124 | 5.0 | 1.07±0.006 | 0.07±0.003 | 6.41±0.394 | 47.5±1.01 | 0.973/0.967 |
| BMA 124 | 7.5 | 0.77±0.015 | 0.04±0 | 12.71±0.315 | 27±0.65 | 0.987/0.986 |
| BMA 124 | 10.0 | 0.95±0.097 | 0.01±0.001 | 11.86±0.454 | 12.3±1.4 | 0.998/0.997 |
| BMA 124 | 15.0 | - | - | - | 2.1±0.02 | n/a |
| BMA 124 | 20.0 | - | - | - | 0.9±0.12 | n/a |
| BMA 125 | 0.0 | 1.26±0.021 | 0.25±0.01 | 5.13±0.164 | 65.2±0.99 | 0.999/0.998 |
| BMA 125 | 1.0 | 1.25±0.014 | 0.26±0.017 | 5.64±0.184 | 64.2±1.14 | 0.999/0.999 |
| BMA 125 | 2.5 | 1.22±0.009 | 0.21±0.018 | 5.93±0.031 | 61.9±0.8 | 0.999/1 |
| BMA 125 | 5.0 | 1.11±0.013 | 0.09±0.008 | 10.33±0.103 | 47.1±1.27 | 0.999/0.999 |
| BMA 125 | 7.5 | 1.17±0.013 | 0.12±0.003 | 9.99±0.62 | 51.6±1.04 | 0.999/0.998 |
| BMA 125 | 10.0 | 1.15±0.044 | 0.05±0.01 | 15.66±3.247 | 36.5±5.29 | 0.998/0.998 |
| BMA 125 | 15.0 | - | - | - | 3±0.84 | n/a |
| BMA 125 | 20.0 | - | - | - | 5.8±0.24 | n/a |
| BMA 127 | 0.0 | 1.3±0.055 | 0.21±0.064 | 7.37±0.981 | 63.7±2.67 | 0.99/0.993 |
| BMA 127 | 1.0 | 1.36±0.001 | 0.13±0.001 | 6.35±0.005 | 64.9±0.08 | 0.979/0.981 |
| BMA 127 | 2.5 | 1.34±0.033 | 0.14±0.006 | 7.68±0.045 | 62.7±1.59 | 0.986/0.986 |
| BMA 127 | 5.0 | 1.13±0.016 | 0.09±0.001 | 10.61±0.081 | 47.6±0.38 | 0.997/0.997 |
| BMA 127 | 7.5 | 0.51±0.001 | 0.03±0.001 | 14.7±0.174 | 17.6±0.17 | 0.993/0.994 |
| BMA 127 | 10.0 | 0.37±0.076 | 0.01±0.002 | 21.92±1.967 | 5.2±1.24 | 0.999/0.999 |
| BMA 127 | 15.0 | - | - | - | 0.4±0.09 | n/a |
| BMA 127 | 20.0 | - | - | - | 0.4±0.22 | n/a |
| BMA 147 | 0.0 | 1.27±0.001 | 0.11±0.012 | 5.82±0.081 | 60.1±0.8 | 0.983/0.981 |
| BMA 147 | 1.0 | 1.25±0.01 | 0.26±0 | 7.25±0.049 | 62.5±0.4 | 0.978/0.983 |
| BMA 147 | 2.5 | 1.24±0.03 | 0.23±0.021 | 7.07±0.204 | 61.6±1.31 | 0.968/0.983 |
| BMA 147 | 5.0 | 1.14±0.005 | 0.08±0.002 | 8.55±0.126 | 48.9±0.07 | 0.985/0.983 |
| BMA 147 | 7.5 | 1.16±0.002 | 0.09±0.002 | 8.24±0.511 | 51.4±0.43 | 0.982/0.983 |
| BMA 147 | 10.0 | 1.1±0.006 | 0.05±0.001 | 10.64±0.938 | 40.6±1.02 | 0.977/0.974 |
| BMA 147 | 15.0 | - | - | - | 4.7±3.11 | n/a |
| BMA 147 | 20.0 | - | - | - | 6.7±0.33 | n/a |
| BMA 149 | 0.0 | 1.32±0.005 | 0.24±0.03 | 9.49±0.21 | 62.6±0.6 | 0.996/0.995 |
| BMA 149 | 1.0 | 1.32±0.003 | 0.3±0.007 | 8.83±0.205 | 64±0.21 | 0.996/0.997 |
| BMA 149 | 2.5 | 1.28±0.007 | 0.25±0.007 | 9.43±0.44 | 60.8±0.14 | 0.997/0.997 |
| BMA 149 | 5.0 | 1.14±0.006 | 0.1±0.004 | 13.15±0.997 | 46.1±1.24 | 0.997/0.997 |
| BMA 149 | 7.5 | 1.17±0.002 | 0.11±0.002 | 13.54±0.212 | 47.4±0.22 | 0.995/0.995 |
| BMA 149 | 10.0 | 1.11±0.005 | 0.05±0.003 | 15.32±0.039 | 34.7±0.96 | 0.991/0.992 |
| BMA 149 | 15.0 | - | - | - | 5.1±0.26 | n/a |
| BMA 149 | 20.0 | - | - | - | 5.1±1.26 | n/a |
| BMA 150 | 0.0 | 1.33±0.003 | 0.23±0.006 | 9.11±0.068 | 63.1±0.08 | 0.997/0.997 |
| BMA 150 | 1.0 | 1.31±0.002 | 0.3±0.007 | 9.1±0.113 | 63.2±0.04 | 0.995/0.997 |
| BMA 150 | 2.5 | 1.29±0.002 | 0.25±0.003 | 9.62±0.349 | 60.9±0.3 | 0.997/0.997 |
| BMA 150 | 5.0 | 1.17±0.001 | 0.09±0.008 | 14.95±2.76 | 43.5±4.15 | 0.995/0.997 |
| BMA 150 | 7.5 | 1.18±0.007 | 0.11±0.001 | 12.43±0.178 | 48.8±0.5 | 0.995/0.994 |
| BMA 150 | 10.0 | 1.13±0.011 | 0.04±0.003 | 14.38±0.234 | 35.3±1.06 | 0.992/0.99 |
| BMA 150 | 15.0 | - | - | - | 4.5±0.3 | n/a |
| BMA 150 | 20.0 | - | - | - | 4.9±0.06 | n/a |
| BMA 151 | 0.0 | 1.32±0.009 | 0.22±0.003 | 9.13±0.181 | 62.5±0.2 | 0.997/0.996 |
| BMA 151 | 1.0 | 1.29±0.007 | 0.25±0.002 | 9.37±0.12 | 61.4±0.14 | 0.998/0.998 |
| BMA 151 | 2.5 | 1.29±0.014 | 0.23±0.032 | 9.78±1.298 | 60.3±1.67 | 0.998/0.998 |
| BMA 151 | 5.0 | 1.16±0.004 | 0.09±0.002 | 12.81±0.592 | 46.1±1.01 | 0.994/0.994 |
| BMA 151 | 7.5 | 1.18±0.003 | 0.11±0 | 13.17±0.127 | 47.5±0.03 | 0.995/0.994 |
| BMA 151 | 10.0 | 1.15±0.024 | 0.04±0.003 | 14.59±0.35 | 32.5±1.27 | 0.99/0.99 |
| BMA 151 | 15.0 | - | - | - | 5.2±0.3 | n/a |
| BMA 151 | 20.0 | - | - | - | 4.9±1.11 | n/a |
| BMA 152 | 0.0 | 1.25±0.008 | 0.15±0.003 | 7.58±0.433 | 59.2±1.03 | 0.995/0.998 |
| BMA 152 | 1.0 | 1.31±0.016 | 0.28±0.01 | 8.51±0.157 | 63.8±0.67 | 0.995/0.997 |
| BMA 152 | 2.5 | 1.29±0.021 | 0.25±0.007 | 8.46±0.169 | 62.6±1.06 | 0.995/0.996 |
| BMA 152 | 5.0 | 1.17±0.016 | 0.09±0.007 | 9.84±0.316 | 49.8±0.85 | 0.992/0.992 |
| BMA 152 | 7.5 | 1.18±0.003 | 0.1±0.001 | 9.72±0.13 | 51.3±0.11 | 0.993/0.992 |
| BMA 152 | 10.0 | 1.05±0.004 | 0.05±0.002 | 9.73±0.158 | 39.1±0.54 | 0.985/0.982 |
| BMA 152 | 15.0 | - | - | - | 6.8±0.21 | n/a |
| BMA 152 | 20.0 | - | - | - | 6.3±1.23 | n/a |
| BMA 153 | 0.0 | 1.24±0.015 | 0.16±0.002 | 7.65±0.051 | 59.1±0.79 | 0.997/0.998 |
| BMA 153 | 1.0 | 1.28±0.011 | 0.19±0.017 | 7.89±0.135 | 61.6±0.75 | 0.998/0.997 |
| BMA 153 | 2.5 | 1.27±0.006 | 0.18±0.002 | 7.47±0.022 | 61.6±0.23 | 0.998/0.998 |
| BMA 153 | 5.0 | 1.15±0.005 | 0.09±0.002 | 8.91±0.376 | 50±0.02 | 0.984/0.985 |
| BMA 153 | 7.5 | 1.15±0.029 | 0.1±0.003 | 10.48±3.295 | 49.2±5.04 | 0.996/0.982 |
| BMA 153 | 10.0 | 1.08±0.014 | 0.05±0.005 | 11.44±3.147 | 39.1±4.97 | 0.978/0.997 |
| BMA 153 | 15.0 | - | - | - | 6.6±1.13 | n/a |
| BMA 153 | 20.0 | - | - | - | 5.1±0.29 | n/a |
| BMA 154 | 0.0 | 1.23±0.012 | 0.17±0.007 | 8.11±0.211 | 58.6±1.01 | 0.998/0.997 |
| BMA 154 | 1.0 | 1.28±0.004 | 0.23±0.001 | 8.21±0.032 | 62.2±0.11 | 0.998/0.998 |
| BMA 154 | 2.5 | 1.28±0.001 | 0.21±0.009 | 8.01±0.221 | 62±0.49 | 0.998/0.998 |
| BMA 154 | 5.0 | 1.15±0.001 | 0.09±0.001 | 8.66±0.58 | 50.1±0.59 | 0.988/0.986 |
| BMA 154 | 7.5 | 1.16±0.021 | 0.1±0.002 | 10.69±2.494 | 49.6±3.83 | 0.996/0.988 |
| BMA 154 | 10.0 | 1.1±0.003 | 0.05±0.003 | 9.39±0.912 | 42.7±0.42 | 0.977/0.98 |
| BMA 154 | 15.0 | - | - | - | 4.8±2.4 | n/a |
| BMA 154 | 20.0 | - | - | - | 5.2±0.89 | n/a |
| BMA 155 | 0.0 | 1.29±0.01 | 0.21±0.022 | 6.99±0.209 | 63.9±0.67 | 0.999/0.999 |
| BMA 155 | 1.0 | 1.25±0.012 | 0.28±0.006 | 7.34±0.025 | 62.4±0.53 | 0.981/0.98 |
| BMA 155 | 2.5 | 1.22±0.015 | 0.24±0.001 | 7.45±0.011 | 60.5±0.69 | 0.976/0.972 |
| BMA 155 | 5.0 | 1.15±0.006 | 0.09±0 | 8.74±0.351 | 50.5±0.64 | 0.992/0.992 |
| BMA 155 | 7.5 | 1.17±0.01 | 0.1±0.001 | 8.53±0.23 | 52.5±0.71 | 0.99/0.992 |
| BMA 155 | 10.0 | 1.11±0.009 | 0.05±0 | 11.07±0.717 | 41.2±1.11 | 0.986/0.983 |
| BMA 155 | 15.0 | - | - | - | 7.8±0.15 | n/a |
| BMA 155 | 20.0 | - | - | - | 6.7±0.21 | n/a |
| BMA 156 | 0.0 | 1.24±0.007 | 0.17±0.01 | 7.88±0.158 | 59.4±0.41 | 0.999/0.998 |
| BMA 156 | 1.0 | 1.29±0.009 | 0.22±0.007 | 8.2±0.021 | 62.3±0.55 | 0.998/0.998 |
| BMA 156 | 2.5 | 1.29±0.002 | 0.2±0.001 | 7.74±0.07 | 62.3±0.03 | 0.998/0.998 |
| BMA 156 | 5.0 | 1.16±0 | 0.08±0.001 | 8.57±0.425 | 50.6±0.36 | 0.985/0.984 |
| BMA 156 | 7.5 | 1.18±0.004 | 0.1±0.003 | 8.62±0.283 | 52.8±0.03 | 0.985/0.985 |
| BMA 156 | 10.0 | 1.12±0.003 | 0.05±0 | 8.89±0.343 | 43.6±0.26 | 0.975/0.976 |
| BMA 156 | 15.0 | - | - | - | 7.4±0.6 | n/a |
| BMA 156 | 20.0 | - | - | - | 2.7±3.44 | n/a |
| BMA 157 | 0.0 | 1.19±0.008 | 0.16±0.006 | 7.93±0.229 | 56.9±0.84 | 0.999/0.998 |
| BMA 157 | 1.0 | 1.28±0.004 | 0.23±0.005 | 8.33±0.052 | 62±0.19 | 0.997/0.997 |
| BMA 157 | 2.5 | 1.29±0.003 | 0.2±0.004 | 7.97±0.016 | 62.1±0.25 | 0.997/0.997 |
| BMA 157 | 5.0 | 1.15±0.003 | 0.08±0.005 | 8.95±0.694 | 49.6±0.19 | 0.982/0.984 |
| BMA 157 | 7.5 | 1.16±0.037 | 0.1±0.003 | 8.94±0.143 | 51.5±1.82 | 0.989/0.983 |
| BMA 157 | 10.0 | 1.08±0.017 | 0.05±0.011 | 14.56±2.787 | 35.6±5.59 | 0.984/0.997 |
| BMA 157 | 15.0 | - | - | - | 7.3±1.19 | n/a |
| BMA 157 | 20.0 | - | - | - | 5.6±1.22 | n/a |
| BMA 158 | 0.0 | 1.22±0.1 | 0.43±0.34 | 8.97±3.339 | 59.5±2.89 | 0.992/0.996 |
| BMA 158 | 1.0 | 1.27±0.008 | 0.28±0.05 | 8.99±0.804 | 61.5±0.04 | 0.996/0.996 |
| BMA 158 | 2.5 | 1.26±0.029 | 0.24±0.002 | 8.61±1.618 | 60.9±0.69 | 0.995/0.994 |
| BMA 158 | 5.0 | 1.13±0.029 | 0.07±0.01 | 7.67±3.585 | 48.8±1.67 | 0.98/0.992 |
| BMA 158 | 7.5 | 1.11±0.018 | 0.07±0.005 | 2.76±0.027 | 52.5±0.1 | 0.977/0.976 |
| BMA 158 | 10.0 | 0.99±0.092 | 0.03±0.001 | 5.41±7.656 | 36±5.05 | 0.995/0.965 |
| BMA 158 | 15.0 | - | - | - | 27.8±2.57 | n/a |
| BMA 158 | 20.0 | - | - | - | 5.5±4.25 | n/a |
| BMA 166 | 0.0 | 1.29±0.028 | 0.17±0.003 | 8.23±0.221 | 60.7±0.82 | 0.996/0.998 |
| BMA 166 | 1.0 | 1.27±0.01 | 0.2±0.002 | 7.97±0.38 | 61.4±0 | 0.978/0.995 |
| BMA 166 | 2.5 | 1.23±0.016 | 0.21±0.002 | 7.21±0.097 | 60.8±0.64 | 0.996/0.966 |
| BMA 166 | 5.0 | 1.14±0.009 | 0.07±0.002 | 5.7±1.051 | 50.9±0.48 | 0.975/0.977 |
| BMA 166 | 7.5 | 1.16±0.003 | 0.07±0.005 | 6.12±0.806 | 52.1±0.29 | 0.97/0.976 |
| BMA 166 | 10.0 | 1.12±0.001 | 0.04±0.002 | 4.28±1.598 | 42.7±0.47 | 0.948/0.935 |
| BMA 166 | 15.0 | - | - | - | 8.3±1.07 | n/a |
| BMA 166 | 20.0 | - | - | - | 9.4±3.65 | n/a |
| BMA 167 | 0.0 | 1.26±0.026 | 0.2±0.018 | 13.36±0.183 | 54.2±1.2 | 0.998/0.999 |
| BMA 167 | 1.0 | 1.29±0.006 | 0.22±0.002 | 11.94±0.326 | 57.4±0.2 | 0.998/0.998 |
| BMA 167 | 2.5 | 1.26±0.006 | 0.19±0.005 | 11.62±0.104 | 55.9±0.25 | 0.998/0.998 |
| BMA 167 | 5.0 | 1.14±0.01 | 0.06±0.007 | 19.32±0.154 | 33.7±1.73 | 0.99/0.989 |
| BMA 167 | 7.5 | 1.08±0.064 | 0.05±0.008 | 17.31±0.278 | 33±3.37 | 0.992/0.99 |
| BMA 167 | 10.0 | 0.97±0.179 | 0.01±0.004 | 17.59±5.508 | 10.1±0.89 | 0.924/0.944 |
| BMA 167 | 15.0 | - | - | - | 4±1.81 | n/a |
| BMA 167 | 20.0 | - | - | - | 4.4±1.4 | n/a |
| BMA 179 | 0.0 | 1.28±0.017 | 0.28±0.005 | 10.13±0.212 | 60.3±0.4 | 0.996/0.993 |
| BMA 179 | 1.0 | 1.29±0.001 | 0.24±0.008 | 9.09±0.439 | 61.5±0.65 | 0.996/0.996 |
| BMA 179 | 2.5 | 1.28±0.002 | 0.2±0.014 | 10.12±0.236 | 59.2±0.13 | 0.997/0.998 |
| BMA 179 | 5.0 | 1.17±0.007 | 0.09±0.004 | 11.23±0.928 | 48±0.93 | 0.994/0.992 |
| BMA 179 | 7.5 | 1.16±0.007 | 0.09±0.003 | 9.93±2.178 | 49.6±1.99 | 0.987/0.995 |
| BMA 179 | 10.0 | 1.1±0.023 | 0.04±0.003 | 14.05±2.032 | 34.1±2.68 | 0.996/0.992 |
| BMA 179 | 15.0 | - | - | - | 1.9±0.39 | n/a |
| BMA 179 | 20.0 | - | - | - | 3.3±1.2 | n/a |
| BMA 180 | 0.0 | 1.06±0.001 | 0.2±0.001 | 4.82±0.009 | 55.2±0.03 | 0.997/0.998 |
| BMA 180 | 1.0 | 1.07±0.027 | 0.21±0.01 | 4.86±0.116 | 55.9±1.34 | 0.998/0.998 |
| BMA 180 | 2.5 | 0.82±0.003 | 0.11±0.002 | 4.43±0.056 | 42.1±0.25 | 0.999/0.999 |
| BMA 180 | 5.0 | 0.83±0.015 | 0.06±0.005 | 8.64±0.562 | 35.4±0.36 | 0.998/0.994 |
| BMA 180 | 7.5 | 0.87±0.021 | 0.06±0.003 | 7.53±0.027 | 37.9±1.14 | 0.998/0.997 |
| BMA 180 | 10.0 | 0.8±0.035 | 0.03±0 | 8.22±0.742 | 29.1±0.14 | 0.997/0.998 |
| BMA 180 | 15.0 | - | - | - | 4.1±1.18 | n/a |
| BMA 180 | 20.0 | - | - | - | 1.7±0 | n/a |
| BMA 181 | 0.0 | 1.3±0.005 | 0.33±0.002 | 5.58±0.164 | 67.9±0 | 0.998/0.999 |
| BMA 181 | 1.0 | 1.3±0.005 | 0.39±0 | 5.61±0.003 | 68±0.26 | 0.999/0.999 |
| BMA 181 | 2.5 | 1.27±0.012 | 0.32±0.014 | 5.75±0.135 | 66±0.3 | 0.999/0.998 |
| BMA 181 | 5.0 | 1.16±0.007 | 0.14±0.001 | 8.32±0.127 | 54.4±0.17 | 0.996/0.996 |
| BMA 181 | 7.5 | 1.17±0.009 | 0.16±0.005 | 8.19±0.051 | 55.8±0.18 | 0.996/0.996 |
| BMA 181 | 10.0 | 1.07±0.007 | 0.09±0.008 | 10.28±0.06 | 46±0.31 | 0.993/0.993 |
| BMA 181 | 15.0 | - | - | - | 6.5±0.25 | n/a |
| BMA 181 | 20.0 | - | - | - | 1±0.03 | n/a |
| BMA 182 | 0.0 | 1.24±0.009 | 0.12±0.014 | 5.56±0.697 | 60±0.36 | 0.996/0.994 |
| BMA 182 | 1.0 | 1.23±0.053 | 0.16±0.008 | 6.58±1.037 | 60.2±3.37 | 0.996/0.998 |
| BMA 182 | 2.5 | 1.26±0.013 | 0.17±0.017 | 6.12±0.116 | 62.3±0.21 | 0.999/0.998 |
| BMA 182 | 5.0 | 1.14±0.008 | 0.1±0 | 8.81±0.022 | 51±0.26 | 0.997/0.997 |
| BMA 182 | 7.5 | 1.16±0.004 | 0.12±0 | 8.29±0.136 | 53.2±0.01 | 0.996/0.996 |
| BMA 182 | 10.0 | 1.11±0.005 | 0.07±0 | 8.77±0.093 | 46.4±0.29 | 0.992/0.991 |
| BMA 182 | 15.0 | - | - | - | 10.9±1.03 | n/a |
| BMA 182 | 20.0 | - | - | - | 7.5±0.51 | n/a |
| BMA 183 | 0.0 | 1.25±0.012 | 0.14±0.014 | 9.08±0.15 | 57.4±0.33 | 1/0.99 |
| BMA 183 | 1.0 | 1.31±0.007 | 0.19±0.003 | 10.06±0.614 | 60.1±0.58 | 0.998/0.998 |
| BMA 183 | 2.5 | 1.29±0.007 | 0.18±0.015 | 10.51±0.164 | 58.5±0.04 | 0.999/0.995 |
| BMA 183 | 5.0 | 1.16±0.039 | 0.08±0.002 | 13.25±0.668 | 44±0.68 | 0.996/0.995 |
| BMA 183 | 7.5 | 1.16±0.009 | 0.1±0.012 | 11.44±0.713 | 48.7±1.43 | 0.992/0.995 |
| BMA 183 | 10.0 | 1.08±0.006 | 0.04±0.009 | 16.32±2.002 | 31.2±5.09 | 0.998/0.994 |
| BMA 183 | 15.0 | - | - | - | 3.4±1.81 | n/a |
| BMA 183 | 20.0 | - | - | - | 1.7±2.09 | n/a |
| BMA 184 | 0.0 | 1.25±0.028 | 0.12±0.005 | 5.25±0.9 | 60.8±0.37 | 0.998/0.999 |
| BMA 184 | 1.0 | 1.31±0.006 | 0.15±0.008 | 5.46±0.582 | 64.7±1.37 | 0.998/0.999 |
| BMA 184 | 2.5 | 1.08±0.237 | 0.15±0.007 | 3.3±1.685 | 56.6±9.84 | 0.994/0.997 |
| BMA 184 | 5.0 | 1.18±0.016 | 0.11±0.001 | 9.15±0.23 | 52.5±0.26 | 0.997/0.997 |
| BMA 184 | 7.5 | 1.21±0.001 | 0.13±0.005 | 9.73±0.3 | 54±0.15 | 0.996/0.996 |
| BMA 184 | 10.0 | 1.05±0.087 | 0.06±0.012 | 11.97±0.197 | 40.1±4.71 | 0.995/0.995 |
| BMA 184 | 15.0 | - | - | - | 2.8±1.11 | n/a |
| BMA 184 | 20.0 | - | - | - | 1.3±0.86 | n/a |
| BMA 185 | 0.0 | 1.31±0.016 | 0.22±0.049 | 11.84±1.358 | 58.5±1.45 | 0.991/0.995 |
| BMA 185 | 1.0 | 1.31±0.008 | 0.2±0.021 | 12.06±1.223 | 57.6±2.44 | 0.993/0.996 |
| BMA 185 | 2.5 | 1.31±0.004 | 0.15±0.006 | 11.99±0.493 | 56.3±0.24 | 0.993/0.993 |
| BMA 185 | 5.0 | 1.23±0.006 | 0.05±0.005 | 11.06±1.765 | 42.3±0.46 | 0.985/0.989 |
| BMA 185 | 7.5 | 1.23±0.023 | 0.05±0.003 | 12.75±0.694 | 41.3±1.34 | 0.991/0.984 |
| BMA 185 | 10.0 | 1.83±0.07 | 0.02±0.002 | 14.42±2.116 | 23.3±0.01 | 0.995/0.995 |
| BMA 185 | 15.0 | - | - | - | 5.3±1.15 | n/a |
| BMA 185 | 20.0 | - | - | - | 4.7±1.63 | n/a |
| BMA 186 | 0.0 | 1.27±0.027 | 0.13±0.031 | 6.08±1.051 | 61.3±1.43 | 0.996/0.998 |
| BMA 186 | 1.0 | 1.29±0.007 | 0.14±0.001 | 6.85±0.296 | 61.6±0.14 | 0.999/0.999 |
| BMA 186 | 2.5 | 1.27±0.007 | 0.13±0.001 | 5.11±0.286 | 62.5±0.03 | 0.995/0.995 |
| BMA 186 | 5.0 | 1.19±0.008 | 0.08±0.004 | 9.31±0.312 | 49.6±0.03 | 0.987/0.985 |
| BMA 186 | 7.5 | 1.18±0.025 | 0.08±0.004 | 7.56±1.552 | 51.6±3.19 | 0.988/0.982 |
| BMA 186 | 10.0 | 1.1±0.014 | 0.04±0.01 | 6.89±2.933 | 38.8±7.96 | 0.994/0.986 |
| BMA 186 | 15.0 | - | - | - | 5.2±0.75 | n/a |
| BMA 186 | 20.0 | - | - | - | 4.5±1.52 | n/a |
| BMA 188 | 0.0 | 1.28±0.013 | 0.18±0.019 | 7.55±0.955 | 61.9±1.22 | 0.998/0.995 |
| BMA 188 | 1.0 | 1.29±0.003 | 0.19±0.005 | 7.29±0.227 | 62.6±0.26 | 0.997/0.997 |
| BMA 188 | 2.5 | 1.27±0.001 | 0.12±0.002 | 5.69±0.03 | 61±0.13 | 0.997/0.997 |
| BMA 188 | 5.0 | 1.18±0.008 | 0.07±0.004 | 5.05±1.857 | 52.5±1.19 | 0.985/0.975 |
| BMA 188 | 7.5 | 1.21±0.011 | 0.08±0.007 | 7.09±2.314 | 52.7±1.46 | 0.985/0.983 |
| BMA 188 | 10.0 | 1.16±0.003 | 0.04±0.002 | 10.66±0.253 | 37.9±1.31 | 0.99/0.987 |
| BMA 188 | 15.0 | - | - | - | 6.7±1.47 | n/a |
| BMA 188 | 20.0 | - | - | - | 5.5±2.63 | n/a |
| BMA 189 | 0.0 | 1.26±0.005 | 0.18±0.008 | 6.73±0.418 | 61.8±0.08 | 0.998/0.998 |
| BMA 189 | 1.0 | 1.29±0.001 | 0.19±0.004 | 7.34±0.189 | 62.9±0.21 | 0.998/0.997 |
| BMA 189 | 2.5 | 1.25±0.011 | 0.13±0.002 | 4.34±2.293 | 62.6±2.29 | 0.998/0.998 |
| BMA 189 | 5.0 | 1.17±0.001 | 0.08±0.003 | 7.77±0.14 | 51.2±0.52 | 0.995/0.995 |
| BMA 189 | 7.5 | 1.21±0.007 | 0.09±0.007 | 9.08±0.919 | 51.7±2.12 | 0.996/0.996 |
| BMA 189 | 10.0 | 1.16±0.008 | 0.04±0 | 13.46±0.399 | 36.5±0.49 | 0.993/0.995 |
| BMA 189 | 15.0 | - | - | - | 0.8±0.82 | n/a |
| BMA 189 | 20.0 | - | - | - | 10.9±13.06 | n/a |
| BMA 190 | 0.0 | 1.32±0.002 | 0.22±0.005 | 10.63±0.257 | 60.7±0.32 | 0.995/0.994 |
| BMA 190 | 1.0 | 1.32±0.008 | 0.25±0.003 | 9.98±0.107 | 62±0.26 | 0.996/0.995 |
| BMA 190 | 2.5 | 1.3±0.005 | 0.22±0.018 | 9.62±0.092 | 61±0.73 | 0.996/0.995 |
| BMA 190 | 5.0 | 1.18±0.024 | 0.07±0.001 | 13.58±0.465 | 43.6±1.37 | 0.995/0.994 |
| BMA 190 | 7.5 | 1.26±0.002 | 0.09±0.002 | 13.38±0.294 | 48.3±0.07 | 0.994/0.994 |
| BMA 190 | 10.0 | 1.33±0.048 | 0.04±0.002 | 16.77±0.198 | 34.3±0.48 | 0.99/0.989 |
| BMA 190 | 15.0 | - | - | - | 6.9±1.2 | n/a |
| BMA 190 | 20.0 | - | - | - | 7.2±0.39 | n/a |
| BMA 191 | 0.0 | 1.28±0.017 | 0.15±0.006 | 6.06±0.089 | 62.8±0.88 | 0.999/0.999 |
| BMA 191 | 1.0 | 1.2±0.117 | 0.17±0.006 | 4.78±2.14 | 61.2±3.1 | 0.986/0.996 |
| BMA 191 | 2.5 | 1.24±0.052 | 0.18±0.002 | 5.93±2.737 | 61.9±0.94 | 0.998/0.996 |
| BMA 191 | 5.0 | 1.14±0.016 | 0.1±0.005 | 10.32±1.026 | 49.1±0.95 | 0.993/0.994 |
| BMA 191 | 7.5 | 1.16±0.028 | 0.1±0.001 | 11.96±6.481 | 48.1±6.61 | 0.996/0.995 |
| BMA 191 | 10.0 | 1.08±0.006 | 0.07±0.001 | 15.95±0.221 | 37.2±0.23 | 0.986/0.986 |
| BMA 191 | 15.0 | - | - | - | 3.8±0.61 | n/a |
| BMA 191 | 20.0 | - | - | - | 7.2±5.67 | n/a |
| BMA 192 | 0.0 | 1.14±0.06 | 0.17±0.01 | 7.04±0.991 | 55.8±1.86 | 0.994/0.995 |
| BMA 192 | 1.0 | 1.21±0.095 | 0.16±0.056 | 5.15±2.795 | 60.7±2.91 | 0.988/0.998 |
| BMA 192 | 2.5 | 1.16±0.069 | 0.14±0.022 | 3.45±3.251 | 59.7±0.43 | 0.989/0.996 |
| BMA 192 | 5.0 | 1.17±0.015 | 0.08±0.007 | 8.93±1.901 | 50.3±0.91 | 0.991/0.993 |
| BMA 192 | 7.5 | 1.18±0.028 | 0.08±0.009 | 6.03±3.69 | 53.8±2.29 | 0.994/0.991 |
| BMA 192 | 10.0 | 1.12±0.001 | 0.06±0.004 | 13.97±0.195 | 39.8±0.44 | 0.993/0.993 |
| BMA 192 | 15.0 | - | - | - | 4±0.65 | n/a |
| BMA 192 | 20.0 | - | - | - | 2.4±0.01 | n/a |
| BMA 193 | 0.0 | 1.28±0.003 | 0.16±0.002 | 7.24±0.379 | 61.6±0.41 | 0.999/0.999 |
| BMA 193 | 1.0 | 1.22±0.088 | 0.2±0.013 | 6.59±0.892 | 60.8±3.26 | 0.996/0.996 |
| BMA 193 | 2.5 | 1.29±0.003 | 0.19±0.008 | 7.43±0.335 | 62.8±0.11 | 0.997/0.998 |
| BMA 193 | 5.0 | 1.16±0.003 | 0.1±0 | 9.65±0.098 | 50.5±0.21 | 0.994/0.994 |
| BMA 193 | 7.5 | 1.17±0.01 | 0.11±0.002 | 8.87±0.154 | 52.5±0.45 | 0.994/0.995 |
| BMA 193 | 10.0 | 1.1±0.004 | 0.06±0.001 | 10.04±0.154 | 43.4±0.15 | 0.983/0.982 |
| BMA 193 | 15.0 | - | - | - | 8.3±0.44 | n/a |
| BMA 193 | 20.0 | - | - | - | 6.1±0.41 | n/a |
| BMA 194 | 0.0 | 1.28±0.028 | 0.17±0.016 | 8.93±1.573 | 59.6±0.32 | 0.999/0.997 |
| BMA 194 | 1.0 | 1.3±0.005 | 0.2±0.012 | 8.1±0.245 | 62.4±0.23 | 0.998/0.997 |
| BMA 194 | 2.5 | 1.29±0.004 | 0.19±0.008 | 9.84±0.994 | 59.8±0.91 | 0.998/0.999 |
| BMA 194 | 5.0 | 1.19±0.006 | 0.12±0.002 | 12.49±0.082 | 49.6±0.01 | 0.996/0.996 |
| BMA 194 | 7.5 | 1.18±0.021 | 0.12±0.003 | 9.03±1.888 | 53.3±1.19 | 0.994/0.991 |
| BMA 194 | 10.0 | 1.1±0.021 | 0.08±0.004 | 13.38±0.252 | 42.2±1.37 | 0.994/0.993 |
| BMA 194 | 15.0 | - | - | - | 7.8±0.93 | n/a |
| BMA 194 | 20.0 | - | - | - | 3.9±2.09 | n/a |
| BMA 196 | 0.0 | 1.29±0.009 | 0.33±0.011 | 7.27±0.103 | 65±0.21 | 0.998/0.998 |
| BMA 196 | 1.0 | 1.3±0.003 | 0.31±0.007 | 6.37±0.099 | 66.6±0.1 | 0.997/0.997 |
| BMA 196 | 2.5 | 1.27±0.004 | 0.28±0.003 | 6.45±0.097 | 64.5±0.29 | 0.997/0.997 |
| BMA 196 | 5.0 | 1.15±0.004 | 0.1±0.002 | 6.69±0.661 | 53.5±0.78 | 0.99/0.992 |
| BMA 196 | 7.5 | 1.14±0.012 | 0.11±0 | 6.53±0.262 | 54.1±0.8 | 0.991/0.992 |
| BMA 196 | 10.0 | 1.1±0.013 | 0.06±0.001 | 7.17±0.481 | 46.5±0.01 | 0.979/0.979 |
| BMA 196 | 15.0 | - | - | - | 11.5±0.37 | n/a |
| BMA 196 | 20.0 | - | - | - | 8.3±0.46 | n/a |
| BMA 205 | 0.0 | 1.29±0.015 | 0.21±0.004 | 8.02±0.227 | 62.5±1.06 | 0.998/0.998 |
| BMA 205 | 1.0 | 1.3±0.013 | 0.22±0.018 | 7.93±0.086 | 63.5±1.07 | 0.997/0.997 |
| BMA 205 | 2.5 | 1.28±0 | 0.19±0.007 | 7.45±0.095 | 62.2±0.29 | 0.997/0.997 |
| BMA 205 | 5.0 | 1.13±0.013 | 0.09±0 | 9.13±0.102 | 49.2±0.54 | 0.994/0.994 |
| BMA 205 | 7.5 | 1.16±0.008 | 0.1±0 | 8.84±0.466 | 51.1±0.84 | 0.995/0.994 |
| BMA 205 | 10.0 | 1.05±0.018 | 0.06±0.001 | 10.49±0.621 | 40.5±1.03 | 0.989/0.987 |
| BMA 205 | 15.0 | - | - | - | 6.8±0.7 | n/a |
| BMA 205 | 20.0 | - | - | - | 4.7±0.15 | n/a |
| BMA 206 | 0.0 | 1.26±0.015 | 0.16±0.01 | 6.33±0.628 | 62±0.26 | 0.998/0.999 |
| BMA 206 | 1.0 | 1.3±0.003 | 0.18±0.008 | 6.55±0.072 | 64.1±0.29 | 0.998/0.998 |
| BMA 206 | 2.5 | 1.29±0.005 | 0.15±0.004 | 7.04±0.224 | 61.7±0.08 | 0.996/0.995 |
| BMA 206 | 5.0 | 1.18±0.008 | 0.11±0 | 10±0.212 | 51.5±0.03 | 0.995/0.995 |
| BMA 206 | 7.5 | 1.21±0.004 | 0.12±0.006 | 11.02±0.297 | 52.2±0.12 | 0.994/0.994 |
| BMA 206 | 10.0 | 1.12±0.011 | 0.06±0 | 11.99±0.542 | 42.6±0.25 | 0.996/0.995 |
| BMA 206 | 15.0 | - | - | - | 5.7±0.47 | n/a |
| BMA 206 | 20.0 | - | - | - | 3.2±0.37 | n/a |
| BMA 207 | 0.0 | 1.28±0.009 | 0.13±0.001 | 5.42±0.001 | 62.6±0.42 | 0.996/0.997 |
| BMA 207 | 1.0 | 1.3±0.012 | 0.16±0.016 | 6.39±0.329 | 63.7±0.74 | 0.996/0.994 |
| BMA 207 | 2.5 | 1.29±0 | 0.15±0.007 | 6.7±0.132 | 62.2±0.12 | 0.998/0.997 |
| BMA 207 | 5.0 | 1.19±0.004 | 0.1±0.003 | 10.7±0.212 | 50.7±0.14 | 0.995/0.995 |
| BMA 207 | 7.5 | 1.21±0.003 | 0.13±0 | 10.45±0.148 | 53.1±0.08 | 0.994/0.994 |
| BMA 207 | 10.0 | 1.11±0.002 | 0.07±0.001 | 10.2±0.202 | 44.7±0.51 | 0.995/0.995 |
| BMA 207 | 15.0 | - | - | - | 6.7±0.33 | n/a |
| BMA 207 | 20.0 | - | - | - | 5±0.16 | n/a |
| BMA 208 | 0.0 | 1.2±0.018 | 0.15±0.002 | 5.78±0.369 | 59.3±0.47 | 0.999/0.999 |
| BMA 208 | 1.0 | 1.27±0.008 | 0.21±0.005 | 6.56±0.127 | 63.5±0.31 | 0.997/0.997 |
| BMA 208 | 2.5 | 1.29±0.004 | 0.23±0 | 7.57±0.06 | 63.3±0.08 | 0.997/0.997 |
| BMA 208 | 5.0 | 1.19±0.007 | 0.12±0.002 | 11.37±0.074 | 51.4±0.49 | 0.995/0.995 |
| BMA 208 | 7.5 | 1.21±0.005 | 0.14±0.009 | 11.13±0.213 | 53.2±0.85 | 0.995/0.995 |
| BMA 208 | 10.0 | 1.13±0.001 | 0.08±0.002 | 13.9±0.271 | 43.4±0.51 | 0.994/0.994 |
| BMA 208 | 15.0 | - | - | - | 8.3±0.97 | n/a |
| BMA 208 | 20.0 | - | - | - | 4.9±0.07 | n/a |
| BMA 212 | 0.0 | 1.29±0.002 | 0.22±0.002 | 8.07±0.108 | 62.8±0.01 | 0.994/0.997 |
| BMA 212 | 1.0 | 1.3±0.007 | 0.28±0.007 | 7.75±0.097 | 64.3±0.53 | 0.996/0.996 |
| BMA 212 | 2.5 | 1.28±0.006 | 0.26±0.006 | 7.62±0.136 | 63.4±0.55 | 0.998/0.997 |
| BMA 212 | 5.0 | 1.18±0.016 | 0.1±0.001 | 7.45±0.482 | 53.8±0.12 | 0.99/0.99 |
| BMA 212 | 7.5 | 1.18±0.001 | 0.12±0.003 | 8.02±0.162 | 54.2±0.04 | 0.992/0.991 |
| BMA 212 | 10.0 | 1.09±0.026 | 0.06±0.002 | 8.35±0.176 | 44.3±1.42 | 0.986/0.981 |
| BMA 212 | 15.0 | - | - | - | 8.1±0.76 | n/a |
| BMA 212 | 20.0 | - | - | - | 7.1±2.28 | n/a |
| BMA 214 | 0.0 | 1.07±0.065 | 0.09±0.002 | 9.53±0.101 | 46.8±2.16 | 0.995/0.997 |
| BMA 214 | 1.0 | 1.25±0.008 | 0.18±0.001 | 8.21±0.228 | 59.5±0.62 | 0.995/0.994 |
| BMA 214 | 2.5 | 1.26±0.011 | 0.16±0.013 | 6.97±0.582 | 60.9±0.23 | 0.986/0.981 |
| BMA 214 | 5.0 | 1.17±0.005 | 0.09±0.002 | 15.25±0.134 | 43.2±0.1 | 0.959/0.96 |
| BMA 214 | 7.5 | 1.17±0.01 | 0.08±0.006 | 12.45±0.747 | 45.6±0.19 | 0.948/0.959 |
| BMA 214 | 10.0 | 1.22±0.007 | 0.03±0.002 | 18.28±1.829 | 27.3±2.76 | 0.944/0.931 |
| BMA 214 | 15.0 | - | - | - | 9.6±1.22 | n/a |
| BMA 214 | 20.0 | - | - | - | 6±3.91 | n/a |
| BMA 219 | 0.0 | 1.22±0.016 | 0.11±0.002 | 8.67±0.338 | 54.6±1.17 | 0.998/0.999 |
| BMA 219 | 1.0 | 1.26±0.008 | 0.2±0.007 | 9.39±0.221 | 59.2±0.24 | 0.997/0.997 |
| BMA 219 | 2.5 | 1.26±0.002 | 0.19±0.011 | 10.19±1.632 | 57.8±2.24 | 0.996/0.998 |
| BMA 219 | 5.0 | 1.17±0.004 | 0.09±0.005 | 13.22±0.858 | 45.9±0.4 | 0.975/0.981 |
| BMA 219 | 7.5 | 1.17±0.005 | 0.1±0.005 | 12.04±0.031 | 47.7±0.63 | 0.977/0.982 |
| BMA 219 | 10.0 | 1.13±0.009 | 0.05±0.004 | 16.83±1.533 | 34±0.84 | 0.957/0.965 |
| BMA 219 | 15.0 | - | - | - | 7.5±0.86 | n/a |
| BMA 219 | 20.0 | - | - | - | 7.8±0.48 | n/a |
| BMA 221 | 0.0 | 1.14±0.05 | 0.1±0.001 | 10.66±0.106 | 49±1.64 | 0.998/0.995 |
| BMA 221 | 1.0 | 1.23±0.005 | 0.16±0.009 | 10.43±0.208 | 55.6±0.26 | 0.998/0.998 |
| BMA 221 | 2.5 | 1.22±0.038 | 0.24±0.001 | 11.4±0.025 | 55.6±1.58 | 0.997/0.98 |
| BMA 221 | 5.0 | 1.21±0.002 | 0.12±0.002 | 19.37±0.721 | 42.2±1.03 | 0.992/0.993 |
| BMA 221 | 7.5 | 1.22±0.007 | 0.14±0.001 | 17.59±0.168 | 45.4±0.02 | 0.994/0.993 |
| BMA 221 | 10.0 | 1.12±0.015 | 0.07±0.003 | 26.41±0.447 | 27.5±0.2 | 0.98/0.982 |
| BMA 221 | 15.0 | - | - | - | 6.5±0.02 | n/a |
| BMA 221 | 20.0 | - | - | - | 6.8±0.06 | n/a |
| BMA 222 | 0.0 | 0.85±0.03 | 0.04±0.002 | 2.62±0.052 | 38.6±1.47 | 0.994/0.994 |
| BMA 222 | 1.0 | 1.13±0.046 | 0.03±0.002 | 0±0.002 | 46.1±2.28 | 0.979/0.981 |
| BMA 222 | 2.5 | 1.08±0.05 | 0.04±0.004 | 0.17±0.122 | 45.5±3.54 | 0.977/0.982 |
| BMA 222 | 5.0 | 2.16±0.014 | 0.02±0 | 3.73±0.559 | 31.3±0.03 | 0.948/0.942 |
| BMA 222 | 7.5 | 1.84±0.595 | 0.02±0.002 | 0.01±0.015 | 37.1±1.02 | 0.972/0.952 |
| BMA 222 | 10.0 | 1.47±0.021 | 0.01±0 | 6.15±2.285 | 18.9±1.23 | 0.933/0.944 |
| BMA 222 | 15.0 | - | - | - | 11.9±0.4 | n/a |
| BMA 222 | 20.0 | - | - | - | 7.4±0.02 | n/a |
| BMA 223 | 0.0 | 1.21±0.033 | 0.12±0.008 | 9.65±0.178 | 53.6±0.62 | 0.998/0.999 |
| BMA 223 | 1.0 | 1.28±0.005 | 0.23±0.024 | 9.89±0.267 | 59.8±0.31 | 0.997/0.99 |
| BMA 223 | 2.5 | 1.23±0.006 | 0.25±0.006 | 9.57±0.076 | 58.4±0.09 | 0.971/0.983 |
| BMA 223 | 5.0 | 1.2±0.003 | 0.1±0.002 | 11.69±0.463 | 49.2±0.49 | 0.979/0.975 |
| BMA 223 | 7.5 | 1.2±0.003 | 0.11±0.004 | 11.72±0.6 | 50.3±0.56 | 0.983/0.979 |
| BMA 223 | 10.0 | 1.13±0.001 | 0.07±0 | 17.03±0.615 | 37.3±0.59 | 0.961/0.963 |
| BMA 223 | 15.0 | - | - | - | 9.1±0.09 | n/a |
| BMA 223 | 20.0 | - | - | - | 8.2±0.58 | n/a |
| BMA 224 | 0.0 | 1.27±0.016 | 0.1±0.009 | 8.76±0.108 | 55.3±1.37 | 0.999/0.999 |
| BMA 224 | 1.0 | 1.27±0.017 | 0.15±0.014 | 9±0.228 | 58.4±0.98 | 0.995/0.996 |
| BMA 224 | 2.5 | 1.25±0.007 | 0.16±0.003 | 9.12±0.22 | 58.1±0.13 | 0.996/0.996 |
| BMA 224 | 5.0 | 1.17±0.003 | 0.08±0.018 | 7.17±2.946 | 51.1±1.14 | 0.984/0.993 |
| BMA 224 | 7.5 | 1.16±0.006 | 0.1±0 | 9.76±0.628 | 50.6±0.52 | 0.994/0.993 |
| BMA 224 | 10.0 | 1.1±0.029 | 0.07±0.001 | 11.7±0.615 | 42.4±1.28 | 0.99/0.985 |
| BMA 224 | 15.0 | - | - | - | 7.7±0.26 | n/a |
| BMA 224 | 20.0 | - | - | - | 4.8±0.1 | n/a |
| BMA 225 | 0.0 | 1.33±0.001 | 0.55±0.032 | 5.32±0.024 | 70.7±0.21 | 0.997/0.998 |
| BMA 225 | 1.0 | 1.31±0.005 | 0.51±0.02 | 5.03±0.001 | 70.1±0.35 | 0.998/0.998 |
| BMA 225 | 2.5 | 1.27±0.002 | 0.33±0.002 | 4.82±0.183 | 67±0.13 | 0.999/0.999 |
| BMA 225 | 5.0 | 1.14±0.004 | 0.05±0.006 | 10.45±0.762 | 41.9±0.8 | 0.992/0.992 |
| BMA 225 | 7.5 | 1.14±0 | 0.07±0.002 | 9.82±0.057 | 46.6±0.3 | 0.995/0.995 |
| BMA 225 | 10.0 | 1.77±0.155 | 0.03±0 | 20.58±0.337 | 23±0.25 | 0.972/0.97 |
| BMA 225 | 15.0 | - | - | - | 3.8±0.02 | n/a |
| BMA 225 | 20.0 | - | - | - | 2.1±0.71 | n/a |
| BMA 228 | 0.0 | 1.35±0.01 | 0.14±0.007 | 8.83±0.382 | 61.9±0.23 | 0.997/0.999 |
| BMA 228 | 1.0 | 1.33±0.004 | 0.17±0.002 | 8.86±0.096 | 61.9±0.4 | 0.998/0.998 |
| BMA 228 | 2.5 | 1.31±0.015 | 0.17±0.019 | 9.15±0.156 | 61±1.07 | 0.998/0.998 |
| BMA 228 | 5.0 | 1.17±0.003 | 0.06±0 | 9.57±0.688 | 46.6±0.97 | 0.994/0.994 |
| BMA 228 | 7.5 | 1.23±0.015 | 0.07±0.007 | 9.8±0.455 | 49.6±2.2 | 0.994/0.994 |
| BMA 228 | 10.0 | 1.24±0 | 0.04±0 | 9.39±0.731 | 39.2±0.65 | 0.987/0.988 |
| BMA 228 | 15.0 | - | - | - | 9±0.19 | n/a |
| BMA 228 | 20.0 | - | - | - | 10±0.64 | n/a |
| BMA 298 | 0.0 | 1.35±0.009 | 0.2±0.005 | 10.41±0.1 | 61.5±0.38 | 0.994/0.995 |
| BMA 298 | 1.0 | 1.4±0.01 | 0.18±0.011 | 10.56±0.042 | 62.9±0.76 | 0.993/0.993 |
| BMA 298 | 2.5 | 1.36±0.015 | 0.14±0.001 | 11.26±0.028 | 58.9±0.67 | 0.994/0.994 |
| BMA 298 | 5.0 | 1.09±0.064 | 0.06±0.005 | 15.62±0.716 | 35.9±1.71 | 0.997/0.997 |
| BMA 298 | 7.5 | 1.07±0.017 | 0.02±0 | 26.91±1.314 | 10.2±0.58 | 0.995/0.994 |
| BMA 298 | 10.0 | 0.18±0.02 | 0±0 | 19.15±0.326 | 1.9±0.2 | 0.951/0.939 |
| BMA 298 | 15.0 | - | - | - | 0.3±0 | n/a |
| BMA 298 | 20.0 | - | - | - | 0.6±0.17 | n/a |
| BMA 339 | 0.0 | 1.28±0.007 | 0.13±0.003 | 11.69±0.518 | 54.8±0.75 | 0.996/0.996 |
| BMA 339 | 1.0 | 1.34±0.001 | 0.1±0.003 | 10.95±0.123 | 55±0.26 | 0.994/0.993 |
| BMA 339 | 2.5 | 1.16±0.018 | 0.06±0.001 | 9.84±0.237 | 45.4±0.12 | 0.991/0.989 |
| BMA 339 | 5.0 | 0.78±0.014 | 0.06±0.001 | 14.75±0.445 | 29.2±0.85 | 0.992/0.992 |
| BMA 339 | 7.5 | 1±0.021 | 0.02±0 | 27.6±0.01 | 9±0.24 | 0.993/0.991 |
| BMA 339 | 10.0 | 0.1±0.018 | 0±0 | 0±0 | 1.6±0.33 | 0.961/0.906 |
| BMA 339 | 15.0 | - | - | - | 0.3±0.02 | n/a |
| BMA 339 | 20.0 | - | - | - | 1.1±0.35 | n/a |
| BMA 45 | 0.0 | 1.33±0.002 | 0.24±0.013 | 6.99±0.099 | 66.2±0.17 | 0.998/0.998 |
| BMA 45 | 1.0 | 1.32±0.002 | 0.29±0.009 | 7.11±0.044 | 66.1±0.15 | 0.998/0.998 |
| BMA 45 | 2.5 | 1.29±0.02 | 0.23±0.002 | 6.38±0.215 | 64.9±1.25 | 0.998/0.998 |
| BMA 45 | 5.0 | 1.18±0.003 | 0.09±0.006 | 8.31±0.65 | 52.2±0.29 | 0.985/0.985 |
| BMA 45 | 7.5 | 1.16±0.005 | 0.09±0.001 | 7.44±0.565 | 52.2±0.74 | 0.99/0.986 |
| BMA 45 | 10.0 | 1.15±0.008 | 0.05±0.003 | 9.94±0.765 | 43.2±0.19 | 0.974/0.976 |
| BMA 45 | 15.0 | - | - | - | 9.3±0.4 | n/a |
| BMA 45 | 20.0 | - | - | - | 9.4±0.28 | n/a |
| BMA 46 | 0.0 | 1.24±0.076 | 0.13±0.017 | 8.48±0.923 | 56.9±2.85 | 0.995/0.997 |
| BMA 46 | 1.0 | 1.21±0.003 | 0.07±0.003 | 6.18±0.088 | 53±0.26 | 0.98/0.977 |
| BMA 46 | 2.5 | 0.92±0.008 | 0.09±0.003 | 8.77±0.091 | 41.9±0.6 | 0.991/0.991 |
| BMA 46 | 5.0 | 0.75±0.001 | 0.08±0 | 11.55±0.423 | 32.3±0.37 | 0.987/0.987 |
| BMA 46 | 7.5 | 0.56±0.001 | 0.03±0 | 16.9±0.156 | 18.6±0.13 | 0.988/0.987 |
| BMA 46 | 10.0 | 0.51±0.015 | 0.01±0 | 24.09±0.737 | 5.1±0.03 | 0.993/0.99 |
| BMA 46 | 15.0 | - | - | - | 0.8±0.36 | n/a |
| BMA 46 | 20.0 | - | - | - | 0.3±0.05 | n/a |
| BMA 48 | 0.0 | 1.31±0.006 | 0.18±0.007 | 8.09±0.679 | 62.3±1.39 | 0.999/0.998 |
| BMA 48 | 1.0 | 1.29±0.021 | 0.19±0.007 | 7.44±0.151 | 62.5±0.92 | 0.998/0.997 |
| BMA 48 | 2.5 | 1.28±0.001 | 0.18±0.003 | 7.41±0.072 | 62.1±0.24 | 0.998/0.998 |
| BMA 48 | 5.0 | 1.15±0.004 | 0.07±0.008 | 2.17±1.773 | 54.6±0.39 | 0.985/0.992 |
| BMA 48 | 7.5 | 1.19±0.021 | 0.09±0.002 | 14.17±2.721 | 45.7±2.38 | 0.996/0.995 |
| BMA 48 | 10.0 | 0.92±0.276 | 0.05±0.004 | 19.24±7.442 | 28.1±12.8 | 0.975/0.985 |
| BMA 48 | 15.0 | - | - | - | 5.1±0.26 | n/a |
| BMA 48 | 20.0 | - | - | - | 4.9±0.12 | n/a |
| BMA 50 | 0.0 | 1.07±0.001 | 0.06±0.014 | 5.64±0.148 | 47.4±2.14 | 0.954/0.967 |
| BMA 50 | 1.0 | 1.49±0.293 | 0.04±0.01 | 0.48±0.673 | 56.6±0.41 | 0.908/0.908 |
| BMA 50 | 2.5 | 1.25±0.135 | 0.07±0.03 | 3±1.966 | 55.9±0.28 | 0.955/0.924 |
| BMA 50 | 5.0 | 1.04±0.015 | 0.1±0.001 | 12.09±0.307 | 43.6±0.17 | 0.992/0.991 |
| BMA 50 | 7.5 | 1.05±0.018 | 0.11±0.003 | 11.49±0.589 | 45.2±1.15 | 0.993/0.992 |
| BMA 50 | 10.0 | 0.94±0.029 | 0.06±0.002 | 15.34±0.456 | 33.7±0.56 | 0.976/0.979 |
| BMA 50 | 15.0 | - | - | - | 4.6±2.26 | n/a |
| BMA 50 | 20.0 | - | - | - | 4.7±0.02 | n/a |
| BMA 54 | 0.0 | 1.31±0.001 | 0.3±0.008 | 6.65±0.074 | 66.6±0.06 | 0.998/0.998 |
| BMA 54 | 1.0 | 1.3±0.001 | 0.29±0.007 | 6.34±0.036 | 66.6±0.1 | 0.999/0.999 |
| BMA 54 | 2.5 | 1.28±0.009 | 0.22±0.001 | 5.45±0.101 | 65.3±0.56 | 0.998/0.998 |
| BMA 54 | 5.0 | 1.17±0.001 | 0.08±0.003 | 8.42±3.285 | 50.7±3.5 | 0.996/0.987 |
| BMA 54 | 7.5 | 1.18±0.005 | 0.09±0.003 | 9.02±0.012 | 50.8±0.54 | 0.996/0.995 |
| BMA 54 | 10.0 | 1.14±0.005 | 0.05±0.001 | 9.45±1.601 | 43.5±1.54 | 0.985/0.989 |
| BMA 54 | 15.0 | - | - | - | 8.3±0.12 | n/a |
| BMA 54 | 20.0 | - | - | - | 8.1±1.55 | n/a |
| BMA 56 | 0.0 | 1.19±0.076 | 0.15±0.02 | 9.76±1.943 | 54.3±0.14 | 0.996/0.998 |
| BMA 56 | 1.0 | 1.18±0.126 | 0.17±0.03 | 8.01±2.814 | 56.4±3.06 | 0.995/0.997 |
| BMA 56 | 2.5 | 1.28±0.013 | 0.22±0.022 | 12.14±0.068 | 56.9±0.88 | 0.998/0.998 |
| BMA 56 | 5.0 | 1.17±0.021 | 0.12±0.006 | 21.28±2.549 | 38.8±2.71 | 0.998/0.998 |
| BMA 56 | 7.5 | 1.19±0.002 | 0.13±0.002 | 20.93±0.231 | 39.9±0.31 | 0.998/0.998 |
| BMA 56 | 10.0 | 1.14±0.007 | 0.06±0.008 | 31.95±0.489 | 20.2±1.78 | 0.996/0.996 |
| BMA 56 | 15.0 | - | - | - | 2.2±0.27 | n/a |
| BMA 56 | 20.0 | - | - | - | 2.1±0.32 | n/a |
| BMA 57 | 0.0 | 1.32±0.002 | 0.12±0.006 | 5.37±0.1 | 63.6±0.65 | 0.996/0.995 |
| BMA 57 | 1.0 | 1.31±0.006 | 0.18±0.013 | 6.39±0.111 | 64.6±0.54 | 0.999/0.999 |
| BMA 57 | 2.5 | 1.29±0.004 | 0.19±0.003 | 6.3±0.162 | 64.2±0.44 | 0.999/0.999 |
| BMA 57 | 5.0 | 1.18±0.003 | 0.08±0.003 | 7.11±0.678 | 52.7±0.39 | 0.992/0.993 |
| BMA 57 | 7.5 | 1.21±0.003 | 0.09±0.001 | 7.31±0.088 | 54.6±0.33 | 0.993/0.993 |
| BMA 57 | 10.0 | 1.16±0.004 | 0.05±0 | 8.15±0.114 | 45.2±0.16 | 0.986/0.984 |
| BMA 57 | 15.0 | - | - | - | 8.9±0.31 | n/a |
| BMA 57 | 20.0 | - | - | - | 7.7±0.07 | n/a |
| BMA 60 | 0.0 | 1.32±0.002 | 0.13±0.006 | 6.86±0.529 | 62.3±0.28 | 0.998/0.998 |
| BMA 60 | 1.0 | 1.32±0.003 | 0.19±0.008 | 7.38±0.053 | 63.9±0.05 | 0.997/0.999 |
| BMA 60 | 2.5 | 1.29±0 | 0.19±0.011 | 7.95±1.089 | 62.2±1.11 | 0.998/0.999 |
| BMA 60 | 5.0 | 1.18±0.006 | 0.08±0.001 | 7.57±0.077 | 51.6±0.2 | 0.989/0.988 |
| BMA 60 | 7.5 | 1.2±0.01 | 0.09±0.004 | 7.38±0.824 | 53.7±0.94 | 0.992/0.989 |
| BMA 60 | 10.0 | 1.16±0.002 | 0.05±0.001 | 10.38±0.528 | 40.5±0.14 | 0.974/0.975 |
| BMA 60 | 15.0 | - | - | - | 7.4±1.36 | n/a |
| BMA 60 | 20.0 | - | - | - | 8.4±1.14 | n/a |
| BMA 61 | 0.0 | 0.94±0 | 0.06±0.001 | 3.91±0.079 | 43.4±0.22 | 0.958/0.962 |
| BMA 61 | 1.0 | 1.2±0.023 | 0.12±0.008 | 7.44±0.39 | 56±0.99 | 0.992/0.993 |
| BMA 61 | 2.5 | 1.2±0.019 | 0.13±0.003 | 9.02±0.318 | 54.6±0.54 | 0.992/0.991 |
| BMA 61 | 5.0 | 0.98±0.043 | 0.05±0.001 | 10.47±0.767 | 37.9±0.57 | 0.995/0.994 |
| BMA 61 | 7.5 | 0.68±0.019 | 0.02±0.001 | 13.64±0.588 | 19.7±0.24 | 0.994/0.992 |
| BMA 61 | 10.0 | 0.89±0.029 | 0.01±0.001 | 19.17±0.455 | 10±0.36 | 0.999/0.999 |
| BMA 61 | 15.0 | - | - | - | 0.3±0.03 | n/a |
| BMA 61 | 20.0 | - | - | - | 0.4±0.04 | n/a |
| BMA 63 | 0.0 | 1.14±0.021 | 0.14±0.002 | 9.27±0.495 | 52.1±1.5 | 0.985/0.986 |
| BMA 63 | 1.0 | 1.23±0.001 | 0.28±0.03 | 8.63±0.178 | 59.8±0.08 | 0.981/0.981 |
| BMA 63 | 2.5 | 1.16±0.015 | 0.39±0.04 | 8.9±0.017 | 57.4±0.47 | 0.956/0.95 |
| BMA 63 | 5.0 | 1.14±0.004 | 0.12±0.002 | 13.48±0.165 | 46.7±0.43 | 0.994/0.993 |
| BMA 63 | 7.5 | 1.15±0.001 | 0.13±0.003 | 12.95±0.244 | 48.3±0.11 | 0.994/0.993 |
| BMA 63 | 10.0 | 1±0.013 | 0.06±0.007 | 19.68±1.879 | 31.3±2.51 | 0.987/0.99 |
| BMA 63 | 15.0 | - | - | - | 4±2.46 | n/a |
| BMA 63 | 20.0 | - | - | - | 4±0.99 | n/a |
| BMA 64 | 0.0 | 1.3±0.008 | 0.13±0.002 | 8.56±0.313 | 59.5±0.82 | 0.998/0.999 |
| BMA 64 | 1.0 | 1.31±0.015 | 0.16±0.022 | 6.79±0.23 | 63.4±0.1 | 0.983/0.996 |
| BMA 64 | 2.5 | 1.24±0.034 | 0.18±0.002 | 7.33±0.015 | 60.6±1.56 | 0.997/0.982 |
| BMA 64 | 5.0 | 1.14±0.003 | 0.07±0 | 8.47±0.532 | 48.3±0.7 | 0.984/0.986 |
| BMA 64 | 7.5 | 1.14±0.003 | 0.08±0 | 7.25±0.24 | 50.7±0.11 | 0.985/0.983 |
| BMA 64 | 10.0 | 1.08±0.028 | 0.04±0.004 | 9.66±3.21 | 38.8±2.46 | 0.971/0.957 |
| BMA 64 | 15.0 | - | - | - | 6.9±0.16 | n/a |
| BMA 64 | 20.0 | - | - | - | 6.9±0.86 | n/a |
| BMA 65 | 0.0 | 1.27±0.017 | 0.13±0.009 | 11.22±0.523 | 54.7±0.48 | 0.998/0.999 |
| BMA 65 | 1.0 | 1.24±0.005 | 0.17±0 | 10.69±0.006 | 55.9±0.21 | 0.99/0.991 |
| BMA 65 | 2.5 | 1.22±0.018 | 0.19±0.003 | 10.23±0.115 | 56.5±0.84 | 0.985/0.994 |
| BMA 65 | 5.0 | 1.15±0.004 | 0.1±0.001 | 12.09±0.503 | 47.5±0.67 | 0.996/0.995 |
| BMA 65 | 7.5 | 1.17±0.002 | 0.12±0.002 | 11.87±0.107 | 49.6±0.06 | 0.995/0.995 |
| BMA 65 | 10.0 | 1.06±0.022 | 0.06±0.001 | 16.26±0.657 | 36.3±1.38 | 0.991/0.99 |
| BMA 65 | 15.0 | - | - | - | 5.1±0.64 | n/a |
| BMA 65 | 20.0 | - | - | - | 5±0.05 | n/a |
| BMA 66 | 0.0 | 1.24±0.026 | 0.12±0.007 | 7.48±0.198 | 57.7±1.25 | 0.993/0.994 |
| BMA 66 | 1.0 | 1.29±0.05 | 0.1±0.012 | 7.11±0.471 | 58.8±2.38 | 0.985/0.988 |
| BMA 66 | 2.5 | 1.18±0.044 | 0.08±0.007 | 7.19±0.72 | 51.9±1.46 | 0.991/0.964 |
| BMA 66 | 5.0 | 0.67±0.017 | 0.05±0.004 | 11.91±0.018 | 27.2±0.93 | 0.996/0.995 |
| BMA 66 | 7.5 | 0.47±0.021 | 0.02±0 | 20.5±0.569 | 12.4±0.5 | 0.992/0.993 |
| BMA 66 | 10.0 | 0.2±0.01 | 0±0 | 26±2.298 | 1.8±0.21 | 0.983/0.989 |
| BMA 66 | 15.0 | - | - | - | 0.3±0.02 | n/a |
| BMA 66 | 20.0 | - | - | - | 0.5±0.09 | n/a |
| BMA 70 | 0.0 | 1.3±0.008 | 0.23±0.018 | 8.14±0.47 | 62.9±0.11 | 0.999/0.998 |
| BMA 70 | 1.0 | 1.29±0.033 | 0.29±0.017 | 7.41±0.219 | 64.7±1.64 | 0.986/0.98 |
| BMA 70 | 2.5 | 1.26±0.045 | 0.25±0.015 | 6.98±0.226 | 63±2.18 | 0.994/0.968 |
| BMA 70 | 5.0 | 1.16±0.005 | 0.1±0.008 | 10.66±0.382 | 49.3±0.38 | 0.979/0.977 |
| BMA 70 | 7.5 | 1.14±0.029 | 0.09±0.019 | 12.41±3.765 | 45.4±7.2 | 0.981/0.998 |
| BMA 70 | 10.0 | 1.1±0.002 | 0.07±0.003 | 16.03±0.913 | 38.3±0.57 | 0.966/0.972 |
| BMA 70 | 15.0 | - | - | - | 6.7±0.97 | n/a |
| BMA 70 | 20.0 | - | - | - | 6.9±0.06 | n/a |
| BMA 71 | 0.0 | 0.68±0.056 | 0.1±0.002 | 9.28±0.053 | 31.9±2.41 | 0.996/0.968 |
| BMA 71 | 1.0 | 1.08±0.032 | 0.14±0.009 | 8.07±0.259 | 51.4±1.41 | 0.997/0.997 |
| BMA 71 | 2.5 | 1.2±0.003 | 0.27±0.001 | 8.99±0.081 | 58.1±0.04 | 0.991/0.983 |
| BMA 71 | 5.0 | 1.01±0.009 | 0.1±0.001 | 14.86±0.033 | 39.5±0.38 | 0.997/0.997 |
| BMA 71 | 7.5 | 1.11±0.002 | 0.12±0.001 | 14.37±0.279 | 44.9±0.29 | 0.996/0.996 |
| BMA 71 | 10.0 | 0.73±0.006 | 0.07±0 | 21.54±0.261 | 23.7±0.03 | 0.986/0.985 |
| BMA 71 | 15.0 | - | - | - | 2.7±0.24 | n/a |
| BMA 71 | 20.0 | - | - | - | 2.5±0.47 | n/a |
| BMA 72 | 0.0 | 1.29±0.041 | 0.21±0.018 | 9.77±0.31 | 60.4±0.96 | 0.998/0.978 |
| BMA 72 | 1.0 | 1.28±0.046 | 0.28±0.021 | 8.44±0.15 | 62.6±1.67 | 0.97/0.909 |
| BMA 72 | 2.5 | 1.23±0.015 | 0.28±0 | 7.89±0.182 | 60.9±0.92 | 0.988/0.969 |
| BMA 72 | 5.0 | 1.18±0.001 | 0.1±0.001 | 13.41±2.965 | 46.9±3.64 | 0.997/0.986 |
| BMA 72 | 7.5 | 1.19±0.008 | 0.11±0.001 | 10.47±0.496 | 51.7±0.85 | 0.991/0.987 |
| BMA 72 | 10.0 | 1.12±0.009 | 0.07±0.001 | 16.04±0.121 | 38.5±0.56 | 0.972/0.972 |
| BMA 72 | 15.0 | - | - | - | 5.9±0.03 | n/a |
| BMA 72 | 20.0 | - | - | - | 5.9±0.31 | n/a |
| BMA 8R1 | 0.0 | 1.34±0 | 0.23±0.021 | 9.67±0.391 | 62.9±0.13 | 0.995/0.996 |
| BMA 8R1 | 1.0 | 1.39±0.007 | 0.2±0.004 | 10.1±0.053 | 64.1±0.13 | 0.992/0.991 |
| BMA 8R1 | 2.5 | 1.34±0.011 | 0.19±0.004 | 10.88±0.044 | 60.4±0.63 | 0.993/0.994 |
| BMA 8R1 | 5.0 | 1.25±0.008 | 0.11±0.005 | 14.1±0.34 | 48.9±0.28 | 0.996/0.997 |
| BMA 8R1 | 7.5 | 0.64±0.023 | 0.03±0.001 | 19.3±0.293 | 17.5±0.36 | 0.996/0.996 |
| BMA 8R1 | 10.0 | 0.59±0.095 | 0.01±0.002 | 24.4±0.622 | 5.6±1.08 | 0.988/0.996 |
| BMA 8R1 | 15.0 | - | - | - | 0.3±0.01 | n/a |
| BMA 8R1 | 20.0 | - | - | - | 0.7±0.07 | n/a |
| *Candida boidinii* | 0.0 | 2.22±0.1 | 0.18±0.013 | 11.87±0.975 | 90.6±2.43 | 0.998/0.997 |
| *Candida boidinii* | 1.0 | 2.28±0.051 | 0.17±0.003 | 12.56±0.201 | 90.4±2.38 | 0.998/0.998 |
| *Candida boidinii* | 2.5 | 2.18±0.04 | 0.17±0.026 | 15.43±3.41 | 80.3±6.01 | 0.998/0.998 |
| *Candida boidinii* | 5.0 | 1.95±0.102 | 0.09±0.006 | 25.24±0.316 | 43.2±1.92 | 0.998/0.998 |
| *Candida boidinii* | 7.5 | 0.72±0.046 | 0.02±0.014 | 42.24±9.881 | 3.2±1.61 | 0.925/0.944 |
| *Candida boidinii* | 10.0 | - | - | - | 0.6±0.31 | n/a |
| *Candida boidinii* | 15.0 | - | - | - | 0.6±0.38 | n/a |
| *Candida boidinii* | 20.0 | - | - | - | 0.5±0.12 | n/a |
| *Wickerhamomyces anomalus* | 0.0 | 1.84±0.016 | 0.19±0.071 | 9.81±1.675 | 81±7.85 | 0.965/0.954 |
| *Wickerhamomyces anomalus* | 1.0 | 2.15±0.037 | 0.18±0.004 | 8.91±0.137 | 95.1±2 | 0.993/0.998 |
| *Wickerhamomyces anomalus* | 2.5 | 2.1±0.091 | 0.2±0.015 | 10.7±0.439 | 90.5±3.29 | 0.998/0.997 |
| *Wickerhamomyces anomalus* | 5.0 | 1.94±0.103 | 0.2±0.002 | 13.41±1.159 | 79.3±5.98 | 0.998/0.989 |
| *Wickerhamomyces anomalus* | 7.5 | 2.47±0.145 | 0.1±0.002 | 22.66±0.78 | 56.1±1.04 | 0.996/0.995 |
| *Wickerhamomyces anomalus* | 10.0 | 1.34±0.052 | 0.02±0 | 30.56±1.745 | 10.7±1.08 | 0.998/0.998 |
| *Wickerhamomyces anomalus* | 15.0 | - | - | - | 0.3±0.01 | n/a |
| *Wickerhamomyces anomalus* | 20.0 | - | - | - | 0.3±0.02 | n/a |
| **Because of the lack of growth in 15 and 20% of salt content, AUCs was estimated by using integration of point interpolation instead of Gompertz integration.** | | | | | | |

**Supplementary Table 5:** Statistical Analysis of NaCl conditions Gompertz non lineal regression: Due to the non-normal distribution of the kinetic parameters (Shapiro-Wilk test, p < 0.05) and heteroscedasticity, the non-parametric Kruskal-Wallis rank sum test was employed to assess global differences across salt concentrations. For pairwise comparisons, the Dunn Post-hoc test was performed with Bonferroni adjustment to control the family-wise error rate. Significance levels are indicated as follows: * (p < 0.05), ** (p < 0.01), *** (p < 0.001), and **** (p < 0.0001)."

| **TABLE 1: GLOBAL KRUSKAL-WALLIS TEST SUMMARY (SALT)** | | | | |
| --- | --- | --- | --- | --- |
| Parameter | N | Chi-sq (H) | df | p-value (raw) |
| *A* | 792 | 383.1256 | 5 | 1.28e-80 |
| *AUC* | 1,056 | 897.8147 | 7 | 1.42e-189 |
| *λ* | 792 | 230.5766 | 5 | 8.05e-48 |
| *µ* | 792 | 529.6663 | 5 | 3.15e-112 |

| **TABLE 2: SIGNIFICANT POST-HOC DUNN TEST COMPARISONS (SALT)** | | | | | |
| --- | --- | --- | --- | --- | --- |
| Parameter | Comparison | Z-score | p-value (unadj.) | p-value (Bonf.) | Sig. |
| *A* | 0 vs 5 | -9.4263 | 4.248e-21 | 6.372e-20 | ******** |
|  | 0 vs 7.5 | -8.8315 | 1.033e-18 | 1.549e-17 | ******** |
|  | 0 vs 10 | -12.6207 | 1.624e-36 | 2.436e-35 | ******** |
|  | 1 vs 5 | -11.5744 | 5.553e-31 | 8.329e-30 | ******** |
|  | 1 vs 7.5 | -10.9796 | 4.788e-28 | 7.183e-27 | ******** |
|  | 1 vs 10 | -14.7688 | 2.327e-49 | 3.491e-48 | ******** |
|  | 2.5 vs 5 | -9.2555 | 2.133e-20 | 3.199e-19 | ******** |
|  | 2.5 vs 7.5 | -8.6607 | 4.690e-18 | 7.036e-17 | ******** |
|  | 2.5 vs 10 | -12.4498 | 1.401e-35 | 2.102e-34 | ******** |
|  | 5 vs 10 | -3.1944 | 1.401e-03 | 2.102e-02 | ***** |
|  | 7.5 vs 10 | -3.7892 | 1.511e-04 | 2.267e-03 | ****** |
| *AUC* | 0 vs 5 | -7.8617 | 3.790e-15 | 1.061e-13 | ******** |
|  | 0 vs 7.5 | -7.4920 | 6.784e-14 | 1.899e-12 | ******** |
|  | 0 vs 10 | -12.4060 | 2.425e-35 | 6.789e-34 | ******** |
|  | 0 vs 15 | -17.6902 | 4.989e-70 | 1.397e-68 | ******** |
|  | 0 vs 20 | -18.2355 | 2.699e-74 | 7.557e-73 | ******** |
|  | 1 vs 5 | -9.7729 | 1.471e-22 | 4.120e-21 | ******** |
|  | 1 vs 7.5 | -9.4032 | 5.291e-21 | 1.482e-19 | ******** |
|  | 1 vs 10 | -14.3172 | 1.708e-46 | 4.781e-45 | ******** |
|  | 1 vs 15 | -19.6015 | 1.503e-85 | 4.208e-84 | ******** |
|  | 1 vs 20 | -20.1467 | 2.876e-90 | 8.052e-89 | ******** |
|  | 2.5 vs 5 | -8.1759 | 2.937e-16 | 8.223e-15 | ******** |
|  | 2.5 vs 7.5 | -7.8062 | 5.894e-15 | 1.650e-13 | ******** |
|  | 2.5 vs 10 | -12.7202 | 4.567e-37 | 1.279e-35 | ******** |
|  | 2.5 vs 15 | -18.0044 | 1.799e-72 | 5.037e-71 | ******** |
|  | 2.5 vs 20 | -18.5497 | 8.205e-77 | 2.297e-75 | ******** |
|  | 5 vs 10 | -4.5443 | 5.511e-06 | 1.543e-04 | ******* |
|  | 5 vs 15 | -9.8285 | 8.485e-23 | 2.376e-21 | ******** |
|  | 5 vs 20 | -10.3738 | 3.263e-25 | 9.137e-24 | ******** |
|  | 7.5 vs 10 | -4.9140 | 8.923e-07 | 2.499e-05 | ******** |
|  | 7.5 vs 15 | -10.1982 | 2.019e-24 | 5.654e-23 | ******** |
|  | 7.5 vs 20 | -10.7435 | 6.360e-27 | 1.781e-25 | ******** |
|  | 10 vs 15 | -5.2842 | 1.262e-07 | 3.535e-06 | ******** |
|  | 10 vs 20 | -5.8295 | 5.560e-09 | 1.557e-07 | ******** |
| *λ* | 0 vs 5 | 6.6457 | 3.018e-11 | 4.527e-10 | ******** |
|  | 0 vs 7.5 | 7.2351 | 4.652e-13 | 6.977e-12 | ******** |
|  | 0 vs 10 | 10.6078 | 2.740e-26 | 4.110e-25 | ******** |
|  | 1 vs 5 | 7.2983 | 2.914e-13 | 4.371e-12 | ******** |
|  | 1 vs 7.5 | 7.8878 | 3.077e-15 | 4.615e-14 | ******** |
|  | 1 vs 10 | 11.2605 | 2.056e-29 | 3.084e-28 | ******** |
|  | 2.5 vs 5 | 6.7102 | 1.943e-11 | 2.915e-10 | ******** |
|  | 2.5 vs 7.5 | 7.2997 | 2.885e-13 | 4.327e-12 | ******** |
|  | 2.5 vs 10 | 10.6724 | 1.370e-26 | 2.055e-25 | ******** |
|  | 5 vs 10 | 3.9622 | 7.427e-05 | 1.114e-03 | ****** |
|  | 7.5 vs 10 | 3.3727 | 7.442e-04 | 1.116e-02 | ***** |
| *µ* | 0 vs 5 | -9.7559 | 1.741e-22 | 2.612e-21 | ******** |
|  | 0 vs 7.5 | -9.0362 | 1.622e-19 | 2.433e-18 | ******** |
|  | 0 vs 10 | -15.3733 | 2.472e-53 | 3.707e-52 | ******** |
|  | 1 vs 5 | -11.9971 | 3.680e-33 | 5.521e-32 | ******** |
|  | 1 vs 7.5 | -11.2774 | 1.696e-29 | 2.544e-28 | ******** |
|  | 1 vs 10 | -17.6146 | 1.905e-69 | 2.857e-68 | ******** |
|  | 2.5 vs 5 | -11.1534 | 6.890e-29 | 1.034e-27 | ******** |
|  | 2.5 vs 7.5 | -10.4338 | 1.738e-25 | 2.607e-24 | ******** |
|  | 2.5 vs 10 | -16.7709 | 3.985e-63 | 5.977e-62 | ******** |
|  | 5 vs 10 | -5.6175 | 1.938e-08 | 2.907e-07 | ******** |
|  | 7.5 vs 10 | -6.3371 | 2.341e-10 | 3.512e-09 | ******** |

**Supplementary Table 6:** Kinetic parameters (A, B, C and M) were estimated using the inverse Gompertz decay model. *fa* represents the fractional area. NIC and MIC represent the critical salt thresholds for growth onset inhibition and total suppression, respectively. Values are expressed as mean ± standard deviation (n = 2). The goodness of fit is reported as *R^2^* for each biological weight.

| **Strain** | **A** | **B** | **C** | **M** | **NIC (%)** | **MIC (%)** | **R² (r1/r2)** |
| --- | --- | --- | --- | --- | --- | --- | --- |
| BMA 122 | 0.021±0.0059 | 0.422±0.0368 | 1.08±0.029 | 6.62±0.153 | 2.54±0.51 | 9±0.054 | 0.996 / 0.997 |
| BMA 123 | 0.055±0.0162 | 0.256±0.0735 | 1.04±0.033 | 10.54±0.268 | 3.54±2.276 | 14.61±0.9 | 0.972 / 0.983 |
| BMA 124 | 0.028±0.0026 | 0.308±0.0062 | 1.12±0.004 | 7.68±0.049 | 2.11±0.062 | 10.92±0.115 | 0.994 / 0.996 |
| BMA 125 | 0.055±0.0039 | 0.309±0.0287 | 0.94±0.009 | 11.24±0.513 | 5.66±1.031 | 14.49±0.211 | 0.968 / 0.97 |
| BMA 127 | 0.029±0.0018 | 0.57±0.0734 | 1.02±0.017 | 6.9±0.063 | 3.86±0.455 | 8.67±0.165 | 0.996 / 0.997 |
| BMA 147 | 0.08±0.0191 | 0.375±0.0469 | 0.93±0.018 | 11.84±0.007 | 7.23±0.585 | 14.53±0.329 | 0.98 / 0.975 |
| BMA 149 | 0.057±0.0199 | 0.251±0.0125 | 1±0.021 | 11±0.165 | 4.13±0.177 | 14.99±0.364 | 0.974 / 0.982 |
| BMA 150 | 0.05±0.0031 | 0.254±0.0305 | 0.99±0.021 | 11.12±0.03 | 4.31±0.848 | 15.09±0.447 | 0.978 / 0.947 |
| BMA 151 | 0.058±0.0139 | 0.257±0.011 | 0.99±0.036 | 10.9±0.119 | 4.22±0.404 | 14.79±0.047 | 0.98 / 0.984 |
| BMA 152 | 0.089±0.0142 | 0.335±0.0021 | 0.96±0.014 | 11.75±0.267 | 6.63±0.235 | 14.73±0.286 | 0.982 / 0.98 |
| BMA 153 | 0.074±0.0085 | 0.339±0.0614 | 0.96±0.013 | 11.8±0.452 | 6.64±0.481 | 14.8±0.995 | 0.978 / 0.985 |
| BMA 154 | 0.064±0.005 | 0.376±0.0042 | 0.96±0.011 | 12.16±0.24 | 7.59±0.291 | 14.82±0.21 | 0.976 / 0.98 |
| BMA 155 | 0.088±0.002 | 0.33±0.0033 | 0.89±0.006 | 11.98±0.14 | 6.78±0.191 | 15±0.11 | 0.982 / 0.985 |
| BMA 156 | 0.037±0.052 | 0.359±0.0362 | 0.99±0.069 | 12.68±0.38 | 7.87±0.106 | 15.48±0.663 | 0.983 / 0.986 |
| BMA 157 | 0.1±0.042 | 0.43±0.1271 | 0.95±0.047 | 11.3±0.985 | 7.12±0.252 | 13.74±1.706 | 0.983 / 0.976 |
| BMA 158 | 0±0 | 0.133±0.0211 | 1.21±0.007 | 14.18±0.59 | 1.14±1.473 | 21.78±1.791 | 0.95 / 0.965 |
| BMA 166 | 0.131±0.0461 | 0.387±0.0139 | 0.86±0.06 | 12±0.226 | 7.56±0.066 | 14.59±0.319 | 0.985 / 0.983 |
| BMA 167 | 0.073±0.0316 | 0.291±0.0026 | 1.09±0.011 | 7.73±0.602 | 1.83±0.654 | 11.17±0.572 | 0.962 / 0.965 |
| BMA 179 | 0.03±0.0153 | 0.343±0.0203 | 0.98±0.019 | 11.37±0.364 | 6.35±0.066 | 14.29±0.537 | 0.98 / 0.989 |
| BMA 180 | 0±0 | 0.149±0.0372 | 1.24±0.185 | 9.87±1.146 | 0.44±0.618 | 16.8±0.583 | 0.949 / 0.946 |
| BMA 181 | 0.004±7e-04 | 0.317±0.0051 | 0.99±0.001 | 12.55±0.002 | 7.13±0.089 | 15.7±0.049 | 0.986 / 0.986 |
| BMA 182 | 0.116±0.0073 | 0.397±0.0401 | 0.88±0.035 | 12.77±0.093 | 8.41±0.347 | 15.3±0.349 | 0.982 / 0.982 |
| BMA 183 | 0.02±0.0288 | 0.315±0.0557 | 1.02±0.036 | 11.35±0.835 | 5.82±0.142 | 14.57±1.404 | 0.968 / 0.98 |
| BMA 184 | 0.014±0.0049 | 0.403±0.0286 | 0.98±0.038 | 12.1±0.288 | 7.83±0.015 | 14.59±0.464 | 0.972 / 0.986 |
| BMA 185 | 0.072±0.0236 | 0.239±0.0042 | 1.03±0.013 | 9.62±0.261 | 2.43±0.388 | 13.81±0.187 | 0.984 / 0.987 |
| BMA 186 | 0.058±0.0232 | 0.357±0.0412 | 0.95±0.022 | 11.78±1.065 | 6.94±1.623 | 14.59±0.74 | 0.987 / 0.979 |
| BMA 188 | 0.077±0.0314 | 0.351±0.0761 | 0.94±0.047 | 11.68±0.149 | 6.67±0.936 | 14.59±0.781 | 0.996 / 0.988 |
| BMA 189 | 0.09±0.1095 | 0.382±0.0085 | 0.92±0.121 | 11.16±0.608 | 6.66±0.508 | 13.78±0.666 | 0.988 / 0.931 |
| BMA 190 | 0.094±0.0111 | 0.256±0.003 | 0.96±0.013 | 10.98±0.048 | 4.27±0.128 | 14.89±0.001 | 0.959 / 0.972 |
| BMA 191 | 0.07±0.0608 | 0.328±0.12 | 0.95±0.137 | 11.23±0.002 | 5.62±2.056 | 14.5±1.193 | 0.984 / 0.971 |
| BMA 192 | 0.047±0.0234 | 0.751±0.508 | 0.99±0.031 | 11.52±0.914 | 8.56±1.094 | 13.25±2.083 | 0.988 / 0.984 |
| BMA 193 | 0.087±0.0063 | 0.358±0.0338 | 0.91±0.036 | 12.34±0.027 | 7.51±0.482 | 15.15±0.239 | 0.981 / 0.983 |
| BMA 194 | 0.057±0.0342 | 0.356±0.0318 | 0.95±0.052 | 12.53±0.019 | 7.69±0.45 | 15.35±0.232 | 0.985 / 0.982 |
| BMA 196 | 0.114±0.0087 | 0.318±0.0091 | 0.89±0.006 | 12.36±0.006 | 6.96±0.147 | 15.5±0.096 | 0.984 / 0.981 |
| BMA 205 | 0.058±0.007 | 0.304±0.0232 | 0.96±0.015 | 11.97±0.242 | 6.3±0.673 | 15.26±0.009 | 0.98 / 0.983 |
| BMA 206 | 0.037±0.006 | 0.344±0.0075 | 0.97±0.005 | 12.29±0.038 | 7.3±0.147 | 15.19±0.025 | 0.987 / 0.987 |
| BMA 207 | 0.064±0.0011 | 0.368±0.0011 | 0.93±0.008 | 12.4±0.082 | 7.73±0.068 | 15.11±0.091 | 0.982 / 0.98 |
| BMA 208 | 0.074±0.0018 | 0.356±0.0105 | 0.96±0.004 | 12.44±0.042 | 7.61±0.1 | 15.26±0.125 | 0.981 / 0.985 |
| BMA 212 | 0.097±0.0279 | 0.375±0.0152 | 0.91±0.023 | 12.18±0.429 | 7.6±0.243 | 14.85±0.538 | 0.987 / 0.99 |
| BMA 214 | 0.136±0.0802 | 0.37±0.1785 | 1.07±0.106 | 10.79±1.041 | 5.53±1.495 | 13.85±2.517 | 0.923 / 0.941 |
| BMA 219 | 0.126±0.0018 | 0.332±0.0161 | 0.93±0.01 | 11.34±0.04 | 6.16±0.211 | 14.35±0.186 | 0.978 / 0.979 |
| BMA 221 | 0.133±0.0037 | 0.417±0.0091 | 0.95±0.009 | 10.65±0.103 | 6.53±0.193 | 13.05±0.051 | 0.97 / 0.962 |
| BMA 222 | 0.227±0.032 | 0.437±0.3253 | 0.94±0.121 | 9.88±0.263 | 4.45±3.774 | 13.04±2.613 | 0.899 / 0.922 |
| BMA 223 | 0.149±0.0093 | 0.388±0.0185 | 0.92±0.016 | 11.71±0.221 | 7.28±0.01 | 14.29±0.344 | 0.983 / 0.984 |
| BMA 224 | 0.08±7e-04 | 0.408±0.0396 | 0.95±0.001 | 12.61±0.192 | 8.38±0.603 | 15.07±0.047 | 0.995 / 0.991 |
| BMA 225 | 0.016±0.0096 | 0.168±0.0081 | 1.29±0.043 | 8.2±0.054 | 0±0 | 14.17±0.235 | 0.969 / 0.964 |
| BMA 228 | 0.134±0.0112 | 0.289±0.027 | 0.88±0.023 | 11.45±0.016 | 5.47±0.543 | 14.93±0.342 | 0.972 / 0.968 |
| BMA 298 | 0.017±4e-04 | 0.506±0.0227 | 1.07±0.014 | 6.05±0.171 | 2.66±0.323 | 8.03±0.083 | 0.997 / 0.997 |
| BMA 339 | 0.016±0.0015 | 0.376±0.0067 | 1.13±0.006 | 5.62±0.051 | 1.05±0.031 | 8.28±0.098 | 0.999 / 0.998 |
| BMA 45 | 0.118±0.0054 | 0.297±0.0082 | 0.89±0.015 | 11.68±0.092 | 5.9±0.252 | 15.05±0.001 | 0.981 / 0.98 |
| BMA 46 | 0.004±0.0054 | 0.191±0.0423 | 1.48±0.241 | 4.98±1.284 | 0±0 | 10.33±0.1 | 0.996 / 0.995 |
| BMA 48 | 0.07±0.0165 | 0.425±0.2077 | 0.96±0.044 | 10.3±1.197 | 5.71±1.05 | 12.98±2.505 | 0.993 / 0.997 |
| BMA 50 | 0.082±0.0067 | 0.387±0.0747 | 1.03±0.028 | 11.72±0.04 | 7.2±0.833 | 14.36±0.548 | 0.974 / 0.96 |
| BMA 54 | 0.096±0.0146 | 0.275±0.0057 | 0.92±0.019 | 11.66±0.032 | 5.41±0.163 | 15.29±0.043 | 0.957 / 0.986 |
| BMA 56 | 0.031±0.004 | 0.267±0.0066 | 1.09±0.018 | 9.66±0.093 | 3.23±0.068 | 13.41±0.186 | 0.963 / 0.981 |
| BMA 57 | 0.106±0.0031 | 0.365±1e-04 | 0.89±0.007 | 12.23±0.033 | 7.52±0.034 | 14.97±0.033 | 0.985 / 0.982 |
| BMA 60 | 0.115±0.0213 | 0.366±0.0021 | 0.89±0.014 | 11.7±0.031 | 7±0.004 | 14.43±0.047 | 0.986 / 0.983 |
| BMA 61 | 0.028±0.0019 | 0.382±0.0018 | 1.24±0.008 | 7.76±0.122 | 3.26±0.101 | 10.38±0.134 | 0.953 / 0.956 |
| BMA 63 | 0.068±0.0201 | 0.413±0.0917 | 1.02±0.03 | 11.17±0.438 | 6.9±0.51 | 13.65±0.989 | 0.98 / 0.977 |
| BMA 64 | 0.095±0.0116 | 0.329±0.0086 | 0.94±0.003 | 11.71±0.431 | 6.5±0.567 | 14.75±0.352 | 0.981 / 0.976 |
| BMA 65 | 0.082±0.0035 | 0.416±0.0037 | 0.93±0.008 | 11.83±0.184 | 7.7±0.147 | 14.23±0.206 | 0.989 / 0.99 |
| BMA 66 | 0.014±0.005 | 0.332±0.0379 | 1.22±0.016 | 5.43±0.046 | 0.34±0.479 | 8.46±0.299 | 0.996 / 0.988 |
| BMA 70 | 0.084±0.0061 | 0.249±0.0441 | 1±0.039 | 11±0.567 | 3.99±1.809 | 15.08±0.156 | 0.979 / 0.977 |
| BMA 71 | 0.082±0.0049 | 3.538±3.4889 | 1.34±0.083 | 10.18±0.167 | 9.24±0.765 | 10.73±0.709 | 0.855 / 0.875 |
| BMA 72 | 0.078±0.0061 | 0.333±0.0136 | 0.94±0.005 | 11.76±0.161 | 6.6±0.05 | 14.76±0.283 | 0.962 / 0.982 |
| BMA 8R1 | 0.034±0.0053 | 0.625±0.0013 | 0.99±0.006 | 6.97±0.018 | 4.22±0.013 | 8.57±0.022 | 0.998 / 0.996 |
| *C. boidinii* | 0.009±0.0112 | 0.797±0.3562 | 1.03±0.05 | 5.29±0.079 | 2.9±0.991 | 6.69±0.702 | 0.999 / 0.998 |
| *W. anomalus* | 0.005±0.0065 | 0.603±0.0766 | 1.11±0.076 | 8.73±0.072 | 5.85±0.437 | 10.4±0.141 | 0.98 / 0.992 |
